# SoyCSN: Soybean context‐specific network analysis and prediction based on tissue‐specific transcriptome data

**DOI:** 10.1002/pld3.167

**Published:** 2019-09-17

**Authors:** Juexin Wang, Md Shakhawat Hossain, Zhen Lyu, Jeremy Schmutz, Gary Stacey, Dong Xu, Trupti Joshi

**Affiliations:** ^1^ Department of Electrical Engineering and Computer Science University of Missouri St. Louis MO USA; ^2^ Christopher S. Bond Life Sciences Center University of Missouri St. Louis MO USA; ^3^ Divisions of Plant Science and Biochemistry University of Missouri St. Louis MO USA; ^4^ HudsonAlpha Institute for Biotechnology Huntsville AL USA; ^5^ DOE Joint Genome Institute Walnut Creek CA USA; ^6^ Informatics Institute University of Missouri St. Louis MO USA; ^7^ Department of Health Management and Informatics and Office of Research School of Medicine University of Missouri St. Louis MO USA

**Keywords:** context‐specific network, database, interactome, RNA‐seq, soybean

## Abstract

The Soybean Gene Atlas project provides a comprehensive map for understanding gene expression patterns in major soybean tissues from flower, root, leaf, nodule, seed, and shoot and stem. The RNA‐Seq data generated in the project serve as a valuable resource for discovering tissue‐specific transcriptome behavior of soybean genes in different tissues. We developed a computational pipeline for Soybean context‐specific network (SoyCSN) inference with a suite of prediction tools to analyze, annotate, retrieve, and visualize soybean context‐specific networks at both transcriptome and interactome levels. BicMix and Cross‐Conditions Cluster Detection algorithms were applied to detect modules based on co‐expression relationships across all the tissues. Soybean context‐specific interactomes were predicted by combining soybean tissue gene expression and protein–protein interaction data. Functional analyses of these predicted networks provide insights into soybean tissue specificities. For example, under symbiotic, nitrogen‐fixing conditions, the constructed soybean leaf network highlights the connection between the photosynthesis function and rhizobium–legume symbiosis. SoyCSN data and all its results are publicly available via an interactive web service within the Soybean Knowledge Base (SoyKB) at http://soykb.org/SoyCSN. SoyCSN provides a useful web‐based access for exploring context specificities systematically in gene regulatory mechanisms and gene relationships for soybean researchers and molecular breeders.

## 1 INTRODUCTION

Soybean is one of the most important agricultural crops in the United States, South America, and East Asia (Schmutz et al., 2010). Exploring the mRNA abundance of soybean genes can help understand underlying mechanisms affecting nutritional components (Patil et al., 2015), controlling abiotic signaling (Ghosh & Islam, 2016), organ development (Kim et al., 2016), and physiological metabolic flux (Zhang, Misra, Nargund, Coleman, & Sriram, 2018) in soybean. In many studies, different expression patterns are identified among the same set of genes among different tissues. These tissue specificities can be observed exclusively for closely correlated mechanisms that function in a tissue‐specific manner, such as photosynthesis in the leaf (Bate, Rothstein, & Thompson, 1991) and salinity resistance in the root (McLean, Eubanks, & Meagher, 1990). Following the idea of the Human Genotype‐Tissue Expression Project GTEx (Stranger et al., 2017) and Human Protein Atlas project (Uhlen et al., 2015), the JGI Plant Gene Atlas (https://phytozome.jgi.doe.gov/phytomine/aspect.do?name=Expression) collected the largest number of RNA‐sequencing (RNA‐Seq) data for soybean tissues (Sreedasyam et al., unpublished). This Gene Atlas data include samples from different experimental treatments and stages of 6 major tissues of the soybean, that is, flower, root, leaf, nodule, seed, and shoot and stem. This transcriptome gene atlas dataset provides a great opportunity to investigate and understand tissue‐specific expression patterns and regulation in soybean. In this computational study, we mainly use the term “context specific” instead of “tissue specific” to define gene behavior specificities we aim to capture in the context of sample collections either from individual tissue or from the tissue exclusively under a specific treatment.

Systematically comparing and discovering context specificities by gene expression patterns between tissues are computationally challenging, and hence, a number of different methods have been applied. An ad hoc practice in human studies is to define genes with context specificity as those having mRNA levels in a particular tissue at least five times its average levels (Jain & Tuteja, 2018; Uhlen et al., 2015). This strategy, though intuitive and successful in many cases, is ad hoc by manually defining the threshold and lacks solid statistical support. A top‐down approach provides a unified mathematical model on all the datasets across all the tissues (Bruggeman & Westerhoff, 2007; Shahzad & Loor, 2012), as a “one‐from‐all” strategy. An alternative strategy is bottom‐up, which is focused on exploring the differentially expressed genes (DEGs) between each tissue and all the others in a “one‐against‐all” fashion. The main drawback of bottom‐up studies is that global significant genes are not confidently filtered from aggregation of local pairwise significant genes. Even with application of multiple testing correction, these bottom‐up methods may still suffer from information loss and redundancy. Figure S38 demonstrates a schematic of logical differences between top‐down and bottom‐up analysis approaches.

In this study, we mainly use a top‐down strategy to build context‐specific networks (CSNs). PCA is the most widely used top‐down approach, but classical PCA has its own limitations of its linear assumption (Zhang & Pan, 2015). Recently, Gao et al. developed BicMix, a Bayesian biclustering method, to construct differential gene co‐expression networks (Gao, McDowell, Zhao, Brown, & Engelhardt, 2016). Saha et al. (2017) used this method to construct co‐expression networks on GTEx data across human tissues to study tissue‐specific regulation of transcription and splicing. Xiao, Moreno‐Moral, Rotival, Bottolo, and Petretto (2014) developed a higher‐order generalized singular value decomposition method on multi‐tissue analysis of co‐expression networks. Mohammadi and Grama (2016) identified human tissue‐specific interactomes via convex optimization using transcriptome RNA expression and protein–protein interaction (PPI). These top‐down methods could provide elegant mathematical solutions, but they usually require large datasets as the input, which limits their application in most studies with fewer data points, especially in plant research. The early application in soybean was an EST analysis of soybean roots and shoots (Maguire et al., 2002). There are also some isolated tissue‐specific analysis studies in plant studies, such as analysis of soybean hypersensitive‐induced response protein gene promoter in different tissues (Koellhoffer, Xing, Moon, & Li, 2015). However, due to both data and method limitations, there was no large‐scale context‐specific analysis adopted on all major soybean tissues.

**Figure 1:**
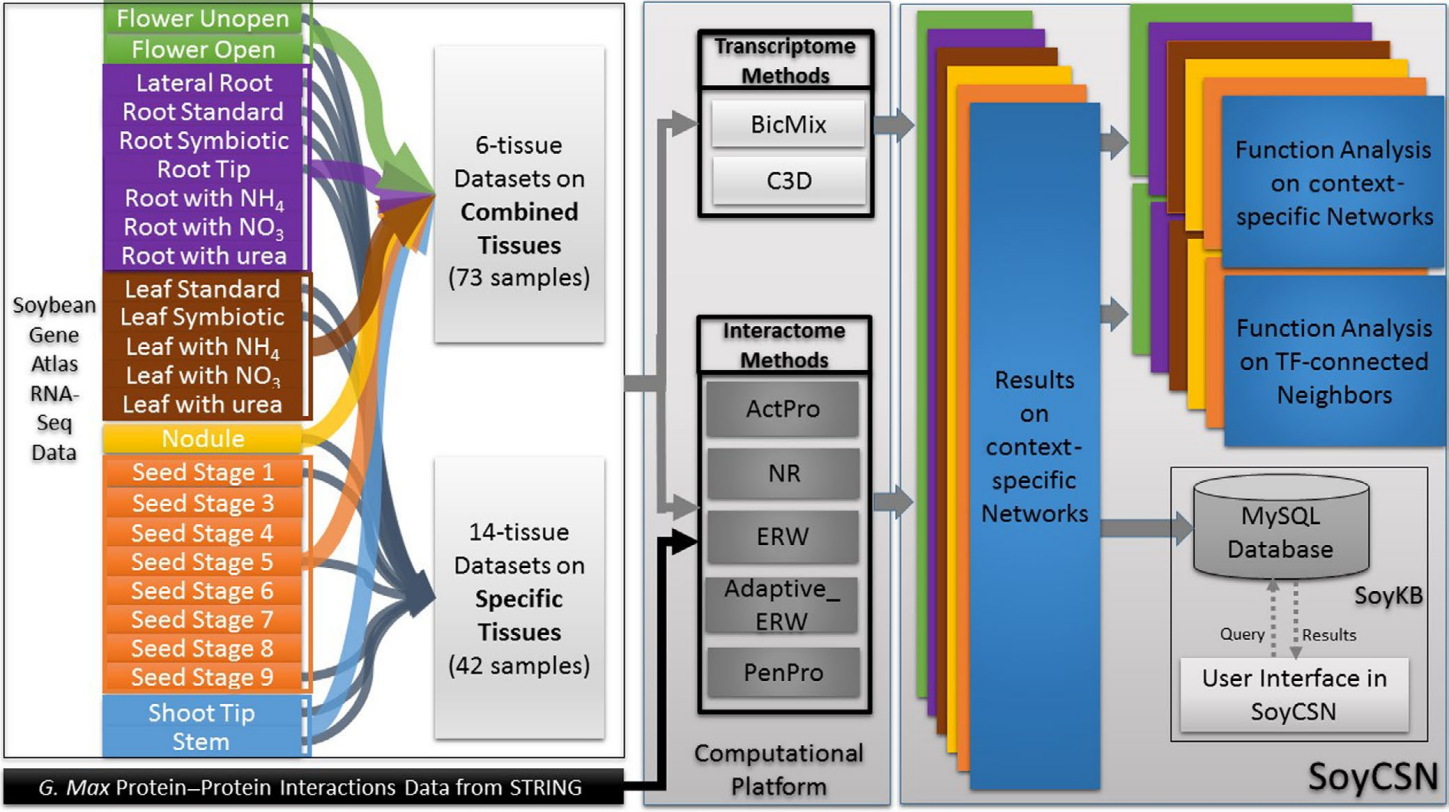
Analysis workflow in SoyCSN. RNA‐Seq data in soybean tissues as leaf (green), root (purple), leaf (brown), nodule (yellow), seed (orange) and shoot‐stem (blue) were collected and organized using both 6 combine tissues and 14 special tissues. Transcriptome analysis methods BicMix and C3D were used to predict context‐specific networks only on RNA‐Seq data. Using RNA‐Seq data with PPI data from the STRING database, context‐specific networks on Interactome were predicted by ActPro, NR, ERW, Adapitve_ERW and PenPro. Function analysis was implemented on both TF connected neighbors and all nodes in context‐specific networks. All the results are stored in MySQL databases and users can access the results in SoyCSN

To our knowledge, this paper is the first effort conducting a systematic exploration of expression specificities and regulatory specificities on all major soybean tissues using large‐scale RNA‐Seq data. After processing Gene Atlas RNA‐Seq data and PPI information from the STRING database (Szklarczyk et al., 2015), soybean CSNs on either six combined tissues or 14 specific tissues were reconstructed using a set of computational approaches such as BicMix (Gao et al., 2016), C3D (Xiao et al., 2014), ActPro, adaptive_ERW, PenPro (Mohammadi & Grama, 2016), NR (Bossi & Lehner, 2009), and ERW (Magger, Waldman, Ruppin, & Sharan, 2012). These CSNs were systematically analyzed and functionally annotated with GO enrichment analysis, pathway analysis, and regulatory motif analysis. All the results can be effectively retrieved and visualized via a web‐based tool in SoyKB, a comprehensive all‐inclusive web resource for soybean (Joshi et al., 2014, 2012, 2017). In this paper, we mainly focused on analyzing and discussing context‐specific network results from transcriptome data, while the interactome data‐based results are available for access and analysis on the website. The whole workflow of this study is shown in Figure 1. This study provides a useful resource for researchers to study tissue specificities in soybean.

## 2 METHODS

### 2.1 Data collection and preprocessing

All the raw RNA‐Seq data were collected from the Joint Genome Institute in Gene Atlas project (phytozome.jgi.doe.gov). In this project, soybean seeds (*G. max* William 82) were surface‐sterilized and transferred to pots containing 3:1 vermiculite:perlite. 2/3 seedlings were planted in each pot and grown until plants were 4 weeks in a growth chamber under 16‐hr light/8‐hr dark conditions, 26–23°C temperature maintained at 250 μmol m^−2^s^−1^. Plants for nitrogen experiment were watered with nutrient solution containing either 10 mM KNO_3_ (NO_3−_ plants) or 10 mM (NH_4_)_3_PO_4_ (NH_4+_ plants) or 5 mM urea (urea plants). Urea was selected as a control condition for the counterions, potassium and phosphate, as the best compromise. The nutrient solutions were renewed every 3 days. After 4 weeks, different tissues (leaf, stem, root, shoot, shoot tip, root tip, lateral roots, etc.) for N regimes and the standard condition were harvested. Plants under the symbiotic conditions were watered with nutrient solution containing 0.5 mM NH_4_NO_3_ every other week. Subsequently, root nodules, roots, and trifoliate leaves under the symbiotic conditions were collected and tissues from flower open and unopen were harvested from field‐grown plants. The RNA‐Seq data that support the findings of this study are available from the NCBI Sequence Read Archive (SRA; https://ncbi.nlm.nih.gov/sar) under accessions provided as in Table S1. More details of the experimental design, plants growth, treatments, library construction, and sequencing are described in a separate paper of Gene Atlas project (Sreedasyam et al., unpublished).

Aligned and annotated using G. max Gene Model Wm82.a2.v1 (Schmutz et al., 2010), the FPKM values of 56,044 genes were obtained through Phytozome interface PhytoMine (https://phytozome.jgi.doe.gov/phytomine/template.do?name=Gene_Expression). To exclude housekeeping genes and genes with small variances across tissues, 15,000 top differential expressed genes across all the samples were selected by median absolute deviation (MAD). For gene expression matrix *Y_i,j_* of *p* genes and *n* samples, i∈[1,p], j∈[1,n] MAD of *i*th gene is defined as Equation (1).

(1) $$\text{MAD}=\text{median}(|y_{i}-\text{median}\left(y_{i}\right)|)$$

### 2.2 Context‐specific network construction using BicMix

BicMix is a Bayesian biclustering method using Bayesian sparse factor analysis (Gao et al., 2016). For gene expression matrix $Y\in {ℜ}^{p\times n}$, BicMix decomposes its value into a sparse loading matrix $\Lambda \in {ℜ}^{p\times K}$, a sparse factor matrix $X\in {ℜ}^{K\times n}$, and the residual error matrix $\varepsilon \in {ℜ}^{p\times n}$, as in Equation (2).

(2) Y=ΛX+ε

*K* is the fixed priori as the number of the biclusters. Sparsity of factors (samples) and loadings (genes) matrices demonstrate the subset of genes for which co‐variation is observed in a subset of samples. Three‐parameter beta (TPB) distribution (Armagan, Dunson, & Clyde, 2011) was used as the prior to induce sparsity in the matrices. The parameters in the model were estimated using both Markov chain Monte Carlo (MCMC) and a variational expectation–maximization (VEM) approach. Based on the components estimated, a Gaussian graphical model (GGM) (Schafer & Strimmer, 2005) was used to infer gene‐by‐gene covariance matrix. Then, GeneNet (Schafer & Strimmer, 2005) was used to test this matrix for significant edges. GeneNet assumes the presence of each edge is drawn from the null (no edge) and alternative (edge) hypothesis.

In this study, we ran BicMix software 1,000 times on our high‐performance computation system. We set parameter 1,000 as the number of components in BicMix. Then, 1,000 results were collected and summarized for datasets both organized in all 6 combined tissues and 14 specific tissues. Finally, edges with a probability > 0.8 were selected as the threshold in GeneNet.

### 2.3 Context‐specific network construction using C3D

Similar to the results obtained from BicMix, all the preselected 15,000 genes with largest MAD were also used as the initial input for the C3D (Cross‐Conditions Cluster Detection) method (Xiao et al., 2014). The high‐order generalized singular value decomposition in C3D method was applied on all these RNA‐Seq data in 73 soybean samples of 6 combined tissues. Ten conservative modules across all 6 tissues were identified by C3D. Some of these modules are specific to several tissues, while others are common in all tissues. From Table S12, we can see large overlaps among these modules identified by C3D, in contrast to the no‐overlap property in the results from BicMix. Different from identifying a subset of specific genes exclusively in the specific tissue from BicMix, C3D infers context‐specific relationships among all the genes. All the results are stored and available via the SoyCSN web service.

C3D can detect both similarity and dissimilarity clustering patterns in large weighted (and unweighted) networks across several conditions. Gene expression matrix $Y\in {ℜ}^{p\times n}$ has *H* conditions, and the raw gene expression under the *h‐*th condition is defined as $Y_{h}\in {ℜ}^{p_{h}\times n}$ (*h* = 1,2, …, *H*, with *H* ≥ 2). In C3D, co‐expression data matrices $E_{h}=Y_{h}^{T}Y_{h}$ for each condition *h* were set from their quadratic form as the initial step by scaling their variance to 1. By high‐order generalized singular value decomposition (Equation 3), matrix *W* (Equation 4) was built on arithmetic mean of all pairwise quotients $E_{h}E_{r}^{+}$, where *E* ^+^ denotes the Moore–Penrose inverse of the co‐expression matrix *E*. The first eigenvectors *V* of *W* were used to identify an approximate decomposition of the input co‐expression matrices. Each selected column vector of *V* was used to reorder the input data matrices that candidate common/differential clusters can be identified.

(3) $$Y_{h}\approx U_{h}\sum_{h}V^{T}$$

(4) $$W=\frac{1}{H(H-1)}\sum_{h=1}^{H}\sum_{r>h}^{H}\left(E_{h}E_{r}^{+}+E_{r}E_{h}^{+}\right)$$

Similar with GeneNet, a mixture model was employed to classify genes to each cluster based on a misclassification error rate (MER). In this study, we used the default threshold 0.05. Finally, an empirical cluster validation procedure was used to identify the contexts where clusters are present and assessed the level of significance within each context.

### 2.4 Context‐specific network on interactome

Different from gene expression context‐specific Network, most previous methods reconstructed tissue‐specific interactomes based on a set of differentially expressed genes in each tissue as the baseline of transcriptional activity and then performed different analysis methods, such as Nodes Removal (NR) (Bossi & Lehner, 2009) and Edge ReWeighting (ERW) (Magger et al., 2012). Along this line, interactome context‐specific networks are built on PPI data. Adopting the topological context of an interaction to infer its specificity, Activity Propagation (ActPro) (Mohammadi & Grama, 2016) is used to construct CSNs on interactome. Similar as BicMix and C3D, preselected 15,000 genes in all samples with highest MAD value were used as the gene expression input. The interactome data were downloaded from the STRING database (Szklarczyk et al., 2015) for organism *Glycine Max*, which contained 56,713,363 predicted soybean PPIs. We also constructed interactome CSNs with NR, ERW, adaptive_ERW, and PenPro. Similar to the results from C3D, each gene has six specific relationships among all tissues, and all the results are stored in SoyCSN website.

The ActPro (Activity Propagation) method formulates the context‐specific interactome inference problem as a suitably regularized convex optimization problem. The objective function for the optimization problem corresponds to a diffusion kernel that propagates activity of genes through interaction, and regularizer α to penalize the difference between transcriptional and functional activity scores, as in Equation (5). The input gene expression data are *Y*, the adjacency matrix of the global interactome is defined as *A*, the element *a_ij_* is the weight (confidence level) of the edge connecting vertices *v_i_* and *v_j_*, and *L* is the Laplacian matrix. These functional activity scores *x* are used to compute context specificity for each edge in the global interactome. ActPro uses CVX (Grant & Boyd, 2008) to solve this convex optimization problem.

(5) $$\begin{array}{c} x^{*}=\arg\min\left\{\frac{a}{\left|E\right|}xLx+\frac{\left(1-a\right)}{\left|V\right|}{\left|x-y\right|}_{1}\right\}\\ s.t.:\left\{\begin{array}{c} 1^{T}x=1\\ 0\leq x \end{array}\right. \end{array}$$

### 2.5 Network and functional analyses

Cytoscape (Shannon et al., 2003) was used to visualize all the networks. Gene Ontology enrichment (Tian et al., 2017), KEGG pathway (Kanehisa & Goto, 2000), and motif enrichment analysis (Bailey et al., 2009) were employed in the functional analysis. agriGO (Tian et al., 2017) was used in the Gene Ontology enrichment, chi‐square was applied as the statistical test method, and Hochberg is employed as the FDR multi‐test adjustment. The analysis was built on complete GO annotation, and the significance level of the results was set as default 0.05. A total of 134 soybean pathways were downloaded from KEGG under the “*Glycine Max*” category. In each of the pathway analyses, we mapped all the input genes to each of the 134 soybean pathways. Top three pathways with most genes mapped were included in the analysis. The MEME suite (Bailey et al., 2009) was used in motif enrichment analysis on 500 bp of the upstream region of each gene downloaded from SoyKB. TomTom (Gupta, Stamatoyannopoulos, Bailey, & Noble, 2007) was used to compare the enriched motifs against the 872 Arabidopsis motifs in Arabidopsis DAPv1 of the DAP motif database (O'Malley et al., 2016) with the threshold *q*‐value of 0.05. The function of the enriched motif was evaluated by Arabidopsis Gene Ontology with GOMo (Boden & Bailey, 2008).

## 3 RESULTS

### 3.1 Incorporating soybean tissue gene expression data in SoyKB

The Soybean Gene Atlas provides soybean gene expression data from RNA‐Seq experiments covering 6 major tissues in soybean, including flower, root, leaf, nodule, seed, and shoot and stem. For most tissues in the analysis, samples are divided into various conditions and collected or treated in different environments. This study used 73 samples under 25 specific tissues in 6 major tissues, including (a) 6 flower samples collected at either flower open or unopen stages; (b) 21 root samples including lateral root, root tip, root standard condition without rhizobium infection, roots in symbiosis with rhizobium inoculation, and root samples treated with NH_4_, NO_3_, or urea; (c) 15 leaf samples including the leaf standard condition without rhizobium infection, leaf symbiosis with rhizobium infection, and leaf samples treated with NH_4_, NO_3_, or urea; (d) 3 nodule samples with symbiotic infection; (e) 22 seed samples in 8 seed developing stages, from early stage 1 to mature stage 9; and (f) 6 shoot and stem samples combining standard shoot tip and stem samples. All the specific tissues under each major tissue have 3 replications, except that seed stages 7 and 8 only have two replications. All the conditions, tissues, and methods were described in JGI website (phytozome.jgi.doe.gov). Note, after the data analysis in this study was completed, additional datasets were generated by JGI, specifically leaf trifoliate developing stages and circadian rhythm in leaf, nodule, and shoot tip. Although these data are not part of the analyses presented, they will be made part of the SoyKB resource.

Aiming to capture different levels of specificities in tissues, analyses were carried out on two selected datasets, that is, 6 combined tissues and refined 14 specific tissues. Table 1 and Table S1 summarize the data used in the study. In the 6‐combined tissue datasets, the context specificity is defined as the specificity of sample collection in each tissue based on the background of all 73 samples. Considering biological significance and physiological functional diversity, 14 specific tissue datasets selected only 14 specific tissues instead of 25, as flower open; flower unopen; lateral roots; root standard; root in symbiosis; root tip; leaf standard; leaf in symbiosis; nodule; seed at stages 1, 5, and 9; shoot tip; and stem were selected to present the diversity across all soybean tissues. In each of the 14 specific tissues, the context specificity is defined based on the background of 42 out of 73 samples.

**Table 1:** Description of data sources and summary annotation of context‐specific networks reconstructed by BicMix. Each combined tissue (left) consists of several specific tissues (right) in different treatments and developing stages

6‐combined tissue datasets			14 specific tissue datasets		
Combined tissue	Sample size	Main annotation	Specific tissues	Sample size	Main annotation
Flower	6	Proteolysis/lipid biosynthesis*	**Flower open**	3	Transferase activity, transferring acyl/hexosyl groups
			**Flower unopen**	3	NA
	21	Carbohydrate biosynthetic process*	**Lateral root**	3	NA
			**Root standard**	3	RNA biosynthetic process/transcription factor activity
			**Root symbiotic**	3	Signaling process
			Root treated with NH_4_	3	
			Root treated with NO_3_	3	
			Root treated with urea	3	
			**Root tip**	3	Translation in ribosome
Leaf	15	Protein folding process	**Leaf standard**	3	Translation in intracellular organelle
			**Leaf symbiotic**	3	Photosynthesis**
			Leaf treated with NH4	3	
			Leaf treated with NO3	3	
			Leaf treated with Urea	3	
Nodule	3	Active transmembrane transporter activity*	**Nodule symbiotic**	3	Cofactor binding/active transmembrane transporter activity
Seed	22	Ribonucleoprotein complex biogenesis/NTP‐dependent helicase activity*	**Seed developing stage 1**	3	Carbohydrate metabolic process/serine‐type peptidase activity*
			Seed developing stage 3	3	
			Seed developing stage 4	3	
			**Seed developing stage 5**	3	NA
			Seed developing stage 6	3	
			Seed developing stage 7	2	
			Seed developing stage 8	2	
			**Seed developing stage 9**	3	Translational initiation/nucleic acid binding in nucleus
Shoot–Stem	6	NA	**Shoot tip**	3	Macromolecule metabolic process/transferase activity, transferring phosphorus‐containing groups
			**Stem**	3	GTP catabolic process/copper ion binding

Note

The bold font in specific tissues indicates 14 representing specific tissues in the 14 specific tissue datasets. The annotations were based on context‐specific networks reconstructed using either overall 6 combined tissues (left) or refined 14 representative specific tissues (right). The main annotations were from GO enrichment analysis using agriGO. NA means no significant results returned from the analysis. Significances of the annotation are tagged with “*,” and “**” means most significant annotations.

**Figure 2:**
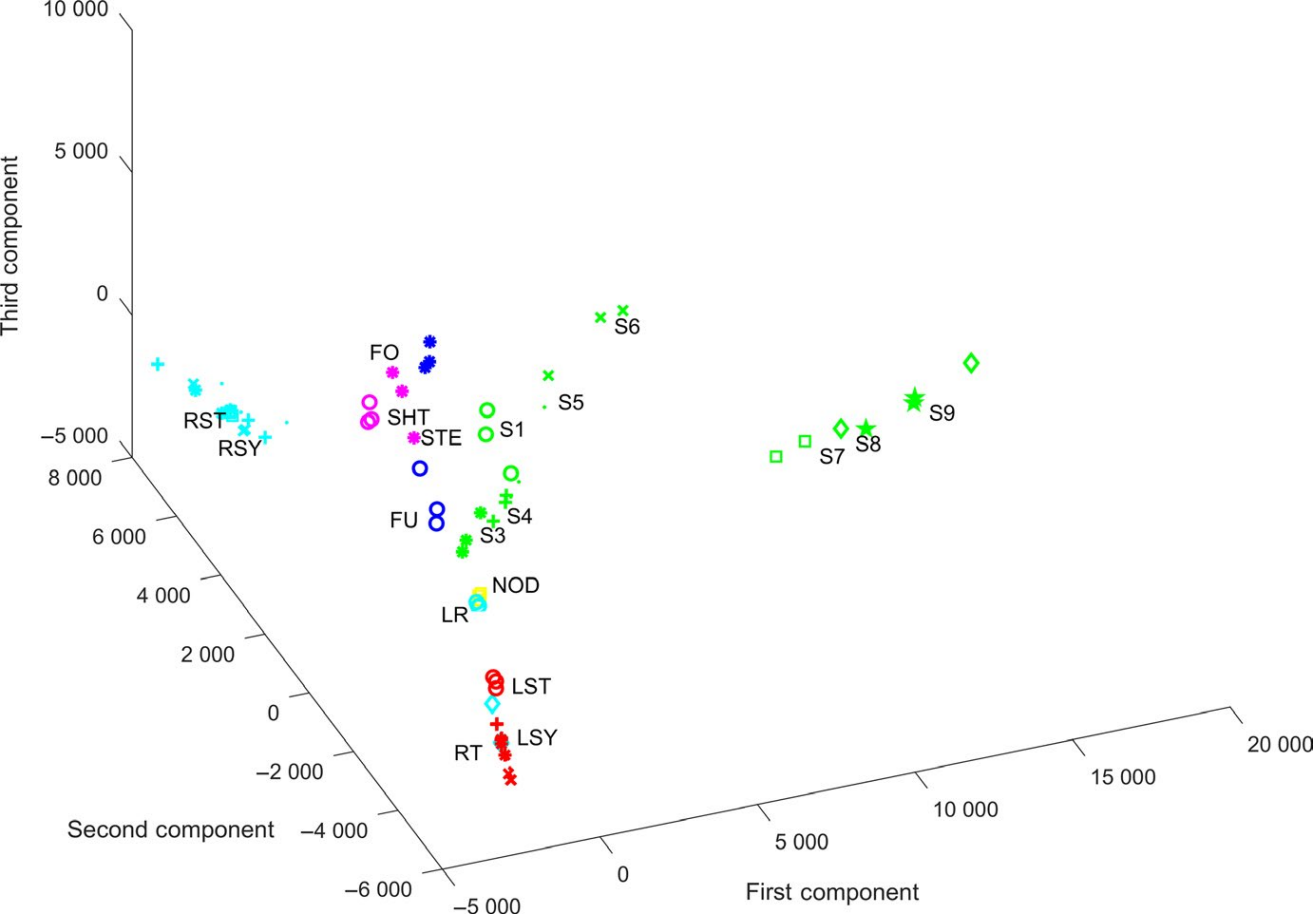
PCA plot of all the 73 soybean samples. Flower unopen (FU) and flower open (FO) samples are drawn as o and * in blue color. Lateral root (LR), root standard (RST), root symbiotic (RSY), root treated with NH_4_, root treated with NO_3_, root treated with urea, roottip (RT) are drawn as o, *, +, dot, cross, square and diamond in cyan. Leaf standard (LST), leaf symbiotic (LSY), leaf treated with NH_4_, leaf treated with NO_3_, leaf treated with Urea are drawn as o, *, +, dot, cross in red. Nodule (NOD) are drawn in yellow. Seed stages 1–9 (S1‐9) are drawn as o, *, +, dot, cross, square, diamond, pentagram in green. Shoottip (SHT) and stem (STE) are drawn as o, * in magenta

To present variations across all these samples, the top 15,000 genes among 56,044 soybean genes in *Glycine Max* Gene Model V9.0 (Schmutz et al., 2010) were selected based on largest median absolute deviation (MAD) with FPKM values in data preprocessing. As the data originate from homogeneous sources, we temporally ignore the batch effect between different samples and treatments in this study. Then, principal component analysis (PCA) was performed on these 15,000 preselected genes. The PCA demonstrates the underlying tissue specialties across all the soybean samples on the gene expression level, which makes this study computationally feasible. Figure 2 shows the PCA plot, in which 73 samples are grouped together in different tissues within top 3 principal components, which explained 39.76%, 20.65%, and 18.23% of the variance. In the plot, spreading samples of seed reveals the largest diversities comparing with other tissues. From stage 1 to stage 9, we can see a clear trajectory of seed development as the expression pattern changes gradually. Seeds in advanced stages are progressively deviating from the original stage, and the developing progress makes seed samples far away from other tissues. The lateral root samples are close to but do not overlap with nodule, and most other root samples are far away from them. Even samples from flower open and unopen conditions are grouped separately; all the flower tissues are still distant from other tissues. Shoot tissue samples and stem tissue samples are close to each other, which justifies combining shoot and stem together in the analysis.

Although the PCA can directly explain most of the relationships among the tissues, it has its own limitations. For example, in the PCA plot, a special root, root tip (RT, cyan diamond in the plot), is far away from another root sample but surprisingly very close to leaf symbiotic samples (LSY, red star in the plot). This may not be biologically meaningful. One potential reason is that PCA performs spectral analysis across all the data, but the difference between two specific tissues may only exist in a specific subset of genes, and this specificity may vanish within the background of all the genes. To overcome this limitation, we performed individual context‐specific analysis on both 6‐combined tissue datasets and refined 14‐specified tissue datasets with refined presentative specific tissues to capture the global and detailed specialties. Table 1 summarizes the data sources, division of the datasets, and the main annotation on each of the CSNs.

### 3.2 Context‐specific network on the 6 combined tissues reconstructed by BicMix

**Figure 3:**
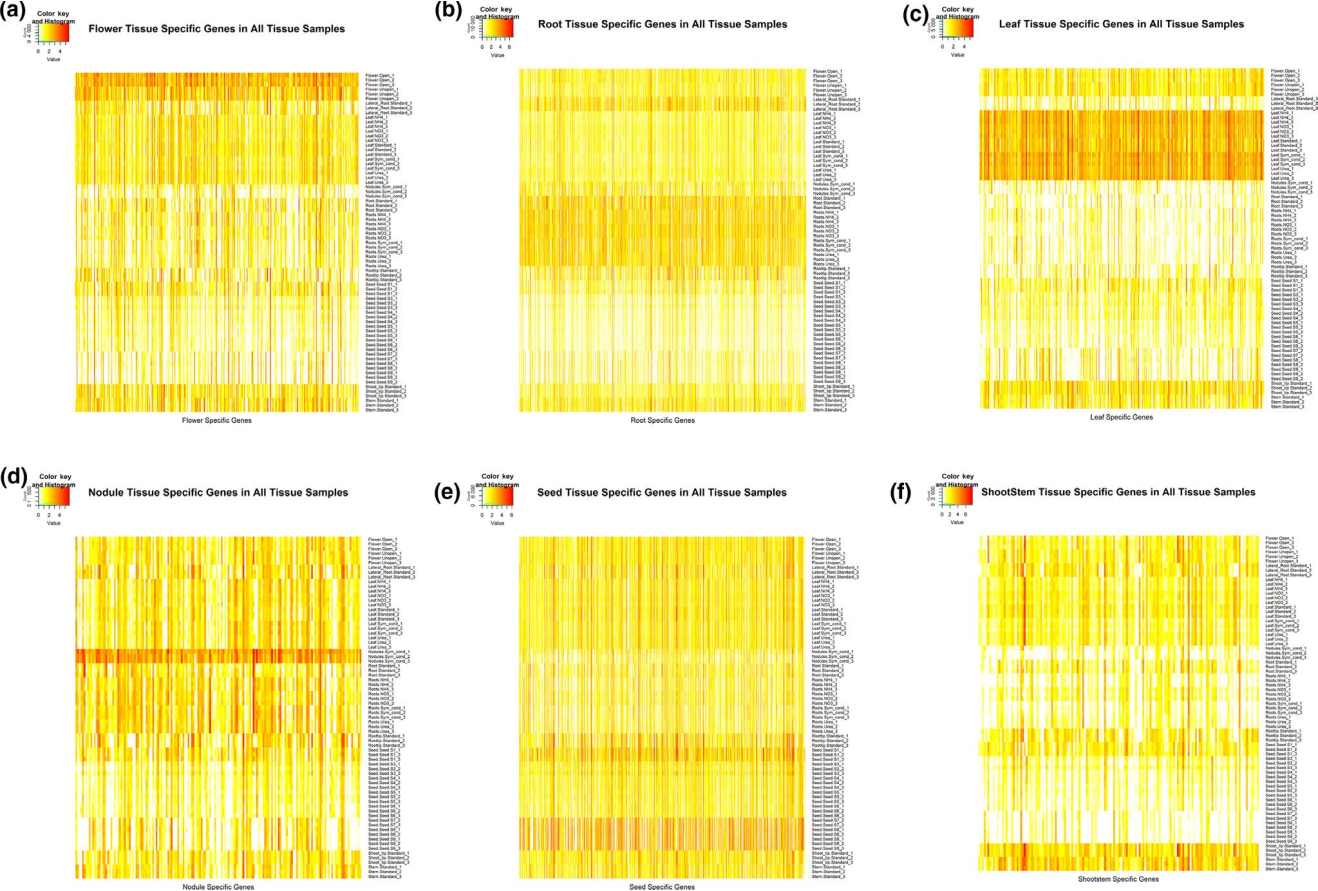
Gene expression levels of genes in six soybean context‐specific networks in 6‐combined tissue datasets. (a) flower context‐specific network, (b) root context‐specific network, (c) leaf context‐specific network, (d) nodule context‐specific network, (e) seed context‐specific network, and (f) shoot and stem context‐specific network. In each of these networks, the horizontal axis indicates genes specific to the tissue, the vertical axis represents all soybean samples. The color scale indicates the expression level. Red box highlights the specific genes in the related tissue.

Six soybean context‐specific networks representing flower, root, leaf, nodule, seed, and shoot and stem combined tissues were reconstructed using BicMix (Gao et al., 2016) on the selected 15,000 variant genes, as described in Figures S1–S6 and Tables S2–S3. In each of these networks, the expression heatmap of identified context‐specific genes and samples demonstrates significant co‐expressed patterns in each of the tissues, as shown in Figure 3. Comparing with the PCA results, root‐ and leaf‐specific genes identified by BicMix are not meshed with root tip samples in the gene expression pattern, which overcomes the issue shown in Figure 1 when using all genes in PCA.

In each of the six CSNs, nodes are context‐specific genes exclusively in this combined tissue, and the edges are context‐specific connections corresponding to co‐expression levels between these genes. Degree of the node is defined as the number of the connections between the node and others. Nodes with large degrees are defined as hubs. These hubs usually have important biological functions and generally play vital roles in the network. Based on the size of the network and analogous work in human studies (Saha et al., 2017), we define three types of hubs by their degrees, small hubs with degrees between 30 and 50, medium hubs with degrees between 50 and 80, and large hubs of degrees more than 80. A summary of nodes, edges, average degree per node, and the number of the hubs is shown in Table 2. The hub genes and their annotations are highlighted in Table S2. It is noted that among these six networks, there is no overlap between any nodes or edges comparing with each other, by definition of context specificity.

**Table 2:** Summary of six context‐specific networks by BicMix (1,000 runs) on 6‐combined tissue datasets

Tissue	Nodes	Edges	Mean degree	#Small Hubs Degree ≥ 30	#Med Hubs Degree ≥ 50	#Large hubs Degree ≥ 80
Flower	359	3,653	20.35	39	51	1
Root	845	9,283	21.97	125	76	8
Leaf	332	4,109	24.75	76	24	4
Nodule	171	1939	22.68	30	11	0
Seed	737	10,124	27.47	33	119	47
Shoot–Stem	154	723	9.39	0	0	0

Although the node degree (number of connections of a node) varies significantly within a context‐specific network, the average node degree is similar among the networks except for the shoot–stem tissue. This observation is also shown in the histogram of node degree in each of these six networks. The average Pearson correlation of all the pairs between the flower, root, leaf, nodule, and seed tissues in the histogram is 0.652. As shown in Figures S7–S8, both the absolute number and the percentage value in the node degree histogram demonstrate the similarities among these networks. The shoot–stem network is different from others, as it has far fewer genes and connections comparing with all the other tissues. Even grouped by similar expression pattern in PCA, combining shoot and stem tissues reveals limited specificities from the CSN model.

Functional analyses were performed on these networks mainly through Gene Ontology (GO) enrichment analysis by agriGO (Tian et al., 2017) and KEGG pathway (Kanehisa & Goto, 2000) analysis. Table S4 shows the GO and KEGG enrichment result for each network. Among these results, the most significant biological process in flower‐specific genes is proteolysis (FDR 1.8E‐04). It is well known that protein degradation largely contributes to produce the visible signs of petal wilting and in‐rolling that typify senescence (Wagstaff et al., 2002), while the functional life of the flower is terminated by senescence and/or abscission. Another possible explanation of proteolysis is the pollen grain (microgametophyte) and the ovule (macrogametophyte) are also included in the analysis, in which proteases play an important role (Radlowski, 2005). In addition, more flower‐specific genes can be mapped to the plant hormone signal transduction pathway than other tissues, which suggests that signaling plays a more important role in the flower tissue than in other tissues. In root tissue, the most significant biological process is the carbohydrate biosynthetic process (FDR 6.1E‐03), which is consistent with the observation that more root‐specific genes are mapped to the carbon metabolism pathway than other tissues. In the leaf tissue combing all leaf samples, only the protein folding process (FDR 0.006) is enriched.

Active transmembrane transporter activity (FDR 0.022) is enriched in the nodule context‐specific network. The roles of transmembrane transporters in soybean nodule development, function, and nitrogen export were reported in the literature (Collier & Tegeder, 2012), and similar results could also be found in Medicago (Yendrek et al., 2010), Lotus (Krusell et al., 2005), and Alder (Jeong et al., 2004). The top three enriched KEGG pathways in nodule tissue are purine metabolism, oxidative phosphorylation, and plant hormone signal transduction. In soybean, the major nitrogen transport products from the nodule are ureides, which are derived from de novo purine biosynthesis (Collier & Tegeder, 2012). Nitrogen fixation is a very energy and reductant intensive process, which is consistent with a strong upregulation of genes involved in respiration and ATP synthesis. Similarly, virtually all plant hormones have been implicated as either positive or negative regulators of nodulation, of which cytokinin is perhaps the most crucial (Ferguson & Mathesius, 2014; Gamas, Brault, Jardinaud, & Frugier, 2017).

The gene enrichment analysis of seed tissue has some interesting results in biological processes, molecular functions, and cellular components. The most significant term in biological process is ribonucleoprotein complex biogenesis (FDR 8.1E‐06). The importance of this pathway in seed is also observed in other studies (Lu et al., 2016). In molecular function enrichments, the energy generation and consumption are highlighted, including purine NTP‐dependent helicase activity (FDR 2.2E‐09), ATP‐dependent helicase activity (FDR 2.2E‐09), and ATPase activity (FDR 4.6E‐08). The cellular component annotation in gene enrichment analysis shows these seed‐specific genes are highly active in ribosome (FDR 5.1E‐07) and cytoplasm (FDR 7.7E‐07), which is consistent with previous studies (Datta, Parker, Averyhart‐Fullard, Schmidt, & Marcus, 1987). In the seed network, more seed‐specific genes are mapped to the ribosome, spliceosome, and protein processing in the endoplasmic reticulum pathway than any other tissue‐specific genes.

Due to small size of the network, the shoot–stem CSN does not have any significant GO enrichment annotations.

Exploring soybean transcriptional factors (TFs) in context‐specific networks may help discover specific regulatory relationships between tissues. After mapping 1697 TFs to these six CSNs, the flower network has 5 TFs, the root network has 27 TFs, the leaf network has 7 TFs, the nodule network has 3 TFs, the seed network has 13 TFs, and the shoot–stem network has 6 TFs. Detailed analyses on TF‐connected genes in 6 CSNs can be found in Table S5. Each TF is assumed to regulate its neighboring genes in the network. In the functional analysis, we focused on those TFs with at least 10 neighboring genes in a network for GO enrichment analysis and pathway analysis. We also conducted the motif enrichment analysis by MEME (Bailey et al., 2009) on 500bp upstream regions of these genes and compared the enriched motifs with known Arabidopsis motifs in the DAP motifs database (O'Malley et al., 2016). Annotated by the Arabidopsis GO terms (Boden & Bailey, 2008), all the enriched motifs have TF activities with *q*‐value less than 0.05 at the 83% specificity.

In the flower network, TF Glyma.17G144700 (HD‐ZIP family) has 51 neighbors. Their enriched annotations are proteolysis (FDR 0.019) and lipid metabolic process (FDR 0.021), which are consistent with the enriched annotations of the whole flower tissue. One motif “CWSYYYYYYTYCYYYTCCCWB” is found among the upstream regions in 40 of 51 genes with *E*‐value of 7.9E‐029, and it is similar (*q*‐value of 2.62E‐03) with motif BBRBPC_tnt.BPC5_colamp_a_m1 associated with TF BPC5, a transcriptional regulator that specifically binds to GA‐rich elements (GAGA‐repeats) present in regulatory sequences of genes involved in developmental processes (Meister et al., 2004). Another TF Glyma.02G196500 (TALE family) regulates 37 neighbors with enriched annotations of oxidation–reduction biological process (FDR 0.0006) and oxidoreductase activity (FDR 0.00017). Motif “AKAWMAMAAAHAARARWGARAAAMRAAAD” with *E*‐value of 2.3E‐07 has been identified in the upstream regions of 34 out of 37 genes. This motif is similar (*q*‐value of 3.01E‐06) to the motif ABI3VP1_tnt.VRN1_col_a_m1 associated with TF VRN1, an Arabidopsis gene involved in regulation of flower development and vernalization response (Levy, Mesnage, Mylne, Gendall, & Dean, 2002). These flower developing regulatory relationships provide support for functional co‐regulatory relationships between the identified TF and its neighbors.

In the root network, TF Glyma.11G056200 (heat shock family) has 128 neighbors with enriched annotations of carbohydrate biosynthetic process (FDR 3.1E‐04), signal transduction (FDR 0.042), and calcium ion binding (FDR 0.0056). Other TFs in the root network and enriched functions of their neighbors include Glyma.09G109700 (protein modification process), Glyma.08G142400 (WRKY transcription factor; signal transduction), Glyma.18G091600 (ERF transcription factor; transcription regulatory activity), and Glyma.20G216600 (carboxylic acid, oxoacid, and organic acid metabolic process). A list of motifs was found among 58 of 59 neighbors of TF Glyma.08G142400, such as motif “CTTYTYTTTTTTTWTTTTTTY,” with *E*‐value of 4.3E‐022. This motif is similar to motif C2C2dof_tnt.AT1G69570_col_a_m1 (*q*‐value of 2.55E‐07) in TF AT1G69570 (CDF5), whose functionality is known to be associated with flower tissue, that is, CDF5 accumulation delays flowering, and links circadian oscillation and photoperiodism (Henriques et al., 2017).

In the leaf network, only TF Glyma.13G325200 (auxin response factor family) has significant GO annotation of molecular ADP binding (FDR 3.6E‐0.5) in its 70 neighbors. The motif “TTTTTWTTYTYTTTH” with *E*‐value of 1.4E‐028 is enriched in 63 out of 70 neighbors. This motif is similar to Dof TF OBP3‐associated motif C2C2dof_tnt.OBP3_col_a_m1 (*q*‐value of 5.53E‐05). In Arabidopsis, OBP3 modulates phytochrome and cryptochrome signaling to perceive subtle changes in light quality and quantity (Ward, Cufr, Denzel, & Neff, 2005). Even though OBP3 is not an orthologue of Glyma.13G325200 in Arabidopsis, the similar motif pattern supports potential functional co‐regulatory relationships in leaves.

In the nodule network, TF Glyma.11G022200 (TALE family) has 42 neighbors with biological process transport (FDR 0.046) annotation enriched. The motif “CTCTCWCYYTCTSTYTCTCY” is enriched with *E*‐value of 1.60E‐014 in part of its neighbors (16 of 42). This motif is similar to motif BBRBPC_tnt.BPC5_colamp_a_m1 (*q*‐value of 1.18E‐09) in TF BPC5 of Arabidopsis.

In the seed network, TF Glyma.14G189300 (NAC family) has 39 neighbors with hydrolase activity; acting on acid anhydrides, phosphorus‐containing anhydride (FDR 0.037) annotations enriched. Motif “YYCWCNBTWWCCYYHHCYTTYTCYCWYYY” with *E*‐value of 6.0E‐027 is identified in 30 out of 39 neighbors. Similar to TF Glyma.17G144700 (HD‐ZIP family) in flower network, this motif is similar to the TF BPC5 associated motif BBRBPC_tnt.BPC5_colamp_a_m1 with *q*‐value of 1.37E‐04. As BPC family regulates a variety of developmental processes that support normal growth and development, mutations in multiple BPC genes lead to pleiotropic effects on vegetative and reproductive development (Meister et al., 2004; Monfared et al., 2011). These similar motifs suggest a similar regulatory function in the seed development.

Considering the importance of hub genes, we also performed Gene Ontology (GO) enrichment analysis only on the hub genes with degree equal or more than 30 on each of the CSNs. The results are detailed in Table S6. The flower tissue shows significant results as catalytic activity (FDR 0.016) and peptidase activity (FDR 0.016). The seed tissue has significant annotations of function structural molecule activity (FDR 3.30E‐06) on ribonucleoprotein complex (FDR 1.00E‐06) with translation process (FDR 0.00026).

### 3.3 Context‐specific networks for refined 14 representative specific tissues reconstructed by BicMix

Refined 14 specific tissue datasets consisting of 42 samples in 14 representative specific tissues were selected for exploring detailed context‐specific functions. Following the same analysis protocol of 73 samples in 6 combined tissues, we built the context‐specific networks for the 14 specific tissues on 15,000 preselected genes (Figures S9–S22, Tables S7–S8). Table 3 provides a summary of nodes, edges, average node degrees, and hub information in these 14 representative specific tissues. The functional analyses of GO enrichment analysis, pathway analysis, and motif enrichment analysis are detailed in Table S9.

**Table 3:** Summary of 14 context‐specific networks by BicMix (1,000 runs) on 14 specific tissue datasets

Specific tissue	Nodes	Edges	Mean degree	#Small Hubs ≥30	#Med Hubs ≥50	#Large hubs ≥80
Flower open	144	3,047	42.32	97	62	2
Flower unopen	15	49	6.53	0	0	0
Lateral root	4	6	3	0	0	0
Leaf standard	22	74	6.73	0	0	0
Leaf symbiotic	113	884	16.65	7	0	0
Nodule	109	2016	36.99	68	28	1
Root standard	138	1937	28.07	77	9	1
Root symbiotic	73	981	26.88	40	0	0
Root tip	12	21	3.5	0	0	0
Seed stage 1	46	587	25.52	33	0	0
Seed stage 5	5	10	4	0	0	0
Seed stage 9	118	973	16.49	11	0	0
Shoot tip	21	86	8.19	0	0	0
Stem	165	3,552	43.05	114	67	17

Different patterns could be observed when comparing constructed CSNs between 6 combined tissues in Table 2 and 14 specific tissues in Table 3. It is much more diverse in specificities from 14 specific tissues than the 6 combined tissues. The differences in mean degree and hub distribution may demonstrate the context‐specific network's capability for capturing diverse specificities in one uniform background.

**Figure 4:**
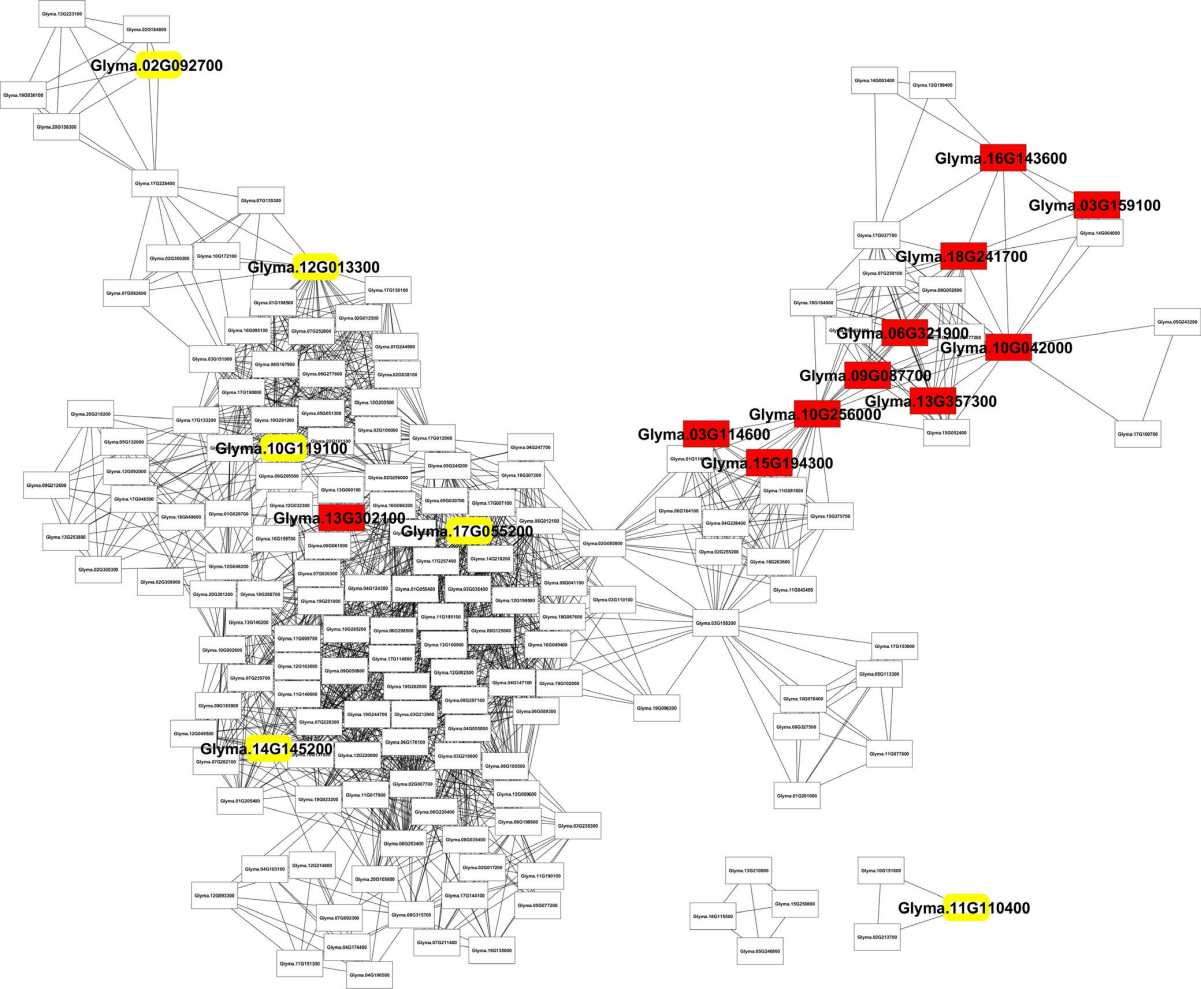
The network specific to the leaf symbiosis with rhizobium infection constructed by BicMix. Nodes are genes specific to leaf with rhizobium‐legume symbiosis, and edges are tissue‐specific connections corresponding to co‐expression levels between these genes. All the transcriptional factors are drawn with yellow border. Genes with photosynthesis annotation are drawn in red

One of the most interesting results was found with the context‐specific network in the leaf symbiotic condition, as shown in Figure 4 and Figure S13. Photosynthesis (FDR 8.9E‐42) is the top enriched biological process in the GO enrichment. Photosystem (FDR 3.1E‐62) and photosynthetic membrane (FDR 7.9E‐62) are top enriched cellular components. Among all the 134 KEGG soybean pathways, the photosynthesis pathway had the most genes mapped. From the figure, most genes with the photosynthesis annotation are grouped together as a functional module. This photosynthesis function in the leaf symbiotic samples is consistent with the basic knowledge on the leaf, where photosynthesis takes place. In contrast, photosynthesis is not enriched in the leaf standard condition, which indicates the rhizobium–legume symbiosis may play an important role in activating soybean photosynthesis, which is consistent with the previous suggestion that fixed nitrogen helps soybean leaves function in photosynthesis normally (Zahran, 1999). Indeed, early whole‐plant studies showed that, at least under conditions of limiting nitrogen, symbiotic nitrogen fixation can significantly enhance soybean leaf photosynthesis (Bethlenfalvay, Abu‐Shakra, & Phillips, 1978). Access to the fixed nitrogen by rhizobium allows soybean to produce leaves fortified with nitrogen that can be recycled throughout the whole plant. This mechanism allows soybean to increase photosynthetic capacity, which in turn yields nitrogen‐rich seeds (Wagner, 2011). Given the large energy requirements of nitrogen fixation, photosynthesis is a crucial component of the integrated process and, indeed, a variety of experiments has documented this (e.g., Giraud et al., 2002; Giraud & Fleischman, 2004).

Comparing all annotations among different content‐specific networks at different scales could provide insights into context specificity. As in Table 1, networks of leaf combined tissues with all leaf samples and leaf standard tissue enriched universal protein folding process and translation in intracellular organelle, which could happen in all biological processes. The photosynthesis annotation is only enriched in the leaf symbiotic samples, not in leaf standard samples without rhizobium–legume infection, and even vanishes in the combined all leaf samples within the background of all tissues. This phenomenon is captured by context‐specific networks reconstructed in this study, and our in silico computational results illustrate the mutually prosperous relationship between rhizobium–legume symbiosis and photosynthesis in detail.

In addition, significant enriched photosynthesis annotation from CSN on effected leaves does not mean photosynthesis is not taken place elsewhere. The heatmap of all genes belonging to the photosynthesis pathway shows nearly equivalent expression among all leaf samples, as in Figure S23. It is usually difficult to infer context‐specific genes adopting an ad hoc approach based on filtering threshold. However, BicMix extracted CSN based on top‐down strategy identified a subset of genes which exactly mapped to photosynthesis pathway.

Another promising result is context network of seed developing stage 1. Carbohydrate metabolic process (FDR 8.3E‐06) is the top enriched biological process. This result is also consistent with the known sugar metabolic activities at the first stage of seed development (Kuo, Doehlert, & Crawford, 1990). Although lipid metabolism pathways should play roles in seed development, they are not significantly enriched in our results. We checked the expression heatmap of all pathways related to lipid metabolism, as in Figures S24–S37.

TFs were also mapped to these 14 CSNs of specific tissues. There are 8 TFs in the flower unopen network; 6 in leaf symbiotic network, nodule network, and shoot tip; 24 in the root standard; 7 in the root symbiotic network, seed at stage 9, and stem; and 2 in the seed network at stage 1. Functional analysis was conducted on the neighbors of each TF in each network, as shown in Table S10. Most annotations on the TF neighbors are closely related to the regulation processes, catalytic activity, and post‐translational protein modification function. In the leaf symbiotic condition, biosynthesis process (FDR 0.018) is enriched in TF Glyma.10G119100 (ethylene response factor family), TF Glyma.12G013300 (Sigma‐70 family), and their 22 neighbors.

Gene enrichment analysis results only on the hub genes with equal or more than 30 degrees in each CSN are detailed in Table S11. Due to the limited number of genes, very few CSNs show significant GO annotations, as root standard CSN with RNA biosynthetic process (FDR 0.0055) and seed stage 9 with nucleic acid binding (FDR 0.0006).

### 3.4 SoyCSN web service

**Figure 5:**
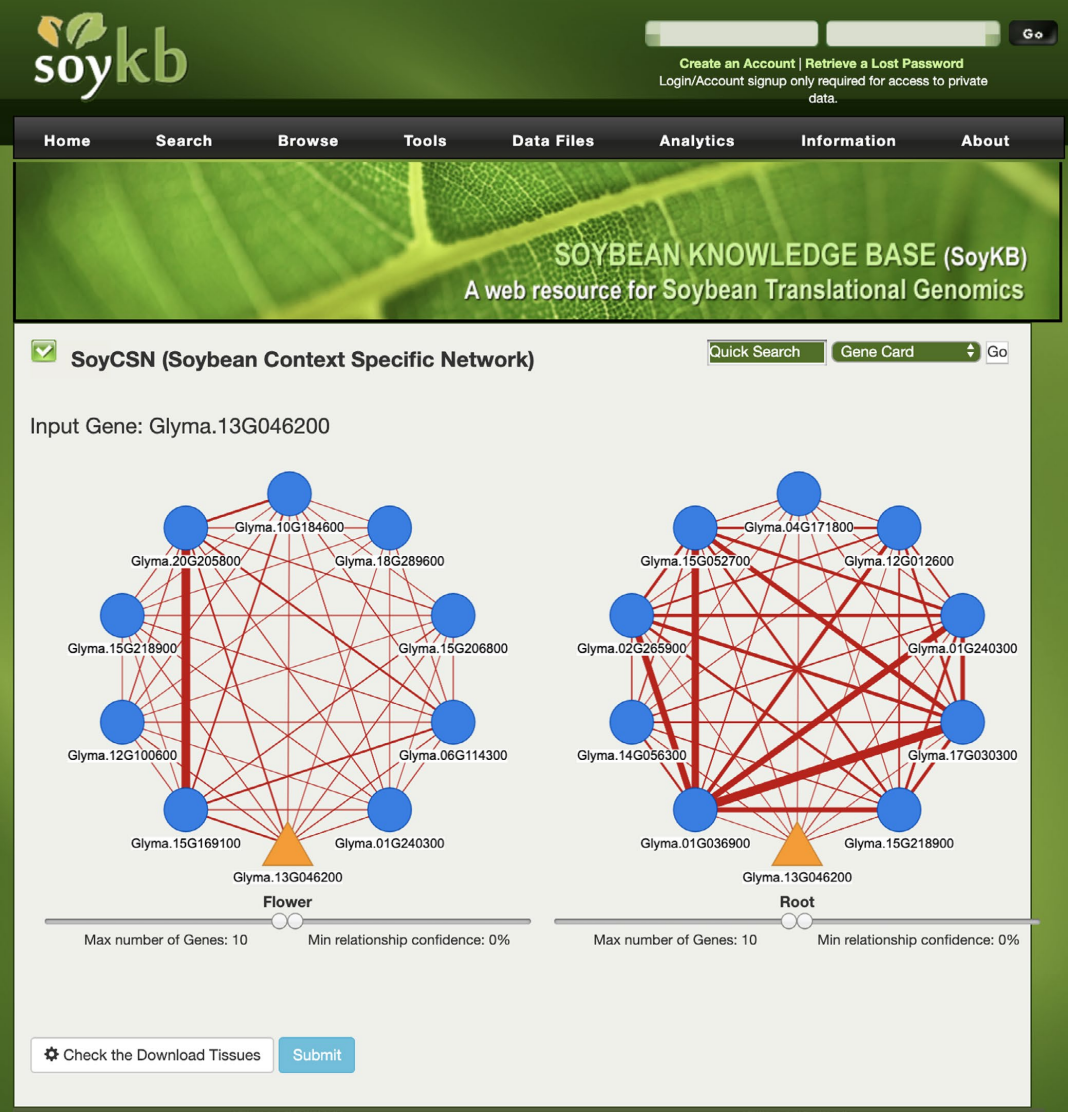
Screen shot of querying Glyma.13G046200 (Ribulose bisphosphate carboxylase) in SoyCSN web service within SoyKB

The web service SoyCSN was established to store, analyze, and visualize all the context‐specific network results generated in this study. It is built as a suite of informatics tools using the SoyKB framework (Joshi et al., 2014, 2012, 2017), where all the results from network constructing methods mentioned above are stored in a MySQL database. These results include transcriptome‐based methods BicMix and C3D, and interactome‐based methods ActPro, NR, ERW, adaptive_ERW, and PenPro. In SoyCSN, users can easily access these precomputed results via different querying parameters by gene names. In the front end, D3.js (Bao & Chen, 2014) is employed to visualize the results. Users can also alter the network visualization parameters, such as the number of the queried genes and filtering based on relationship confidences. Gene functional annotations from SoyKB can be conveniently obtained by clicking the gene nodes in the network and navigating the gene card pages. All these CSN networks can be downloaded locally for further analysis or accessed directly at http://soykb.org/SoyCSN . As an example, Figure 5 is the screenshot of querying Glyma.13G046200 (ribulose bisphosphate carboxylase) from the SoyCSN website. This gene encodes ribulose‐1,5‐bisphosphate carboxylase, which catalyzes the carboxylation and hydrolytic cleavage of ribulose‐1,5‐bisphosphate to form two molecules of 3‐phosphoglycerate (Grandbastien, Berry‐Lowe, Shirley, & Meagher, 1986). With the input gene name Glyma.13G046200, a user can select the gene of interest either in 6‐combined tissue or 14 specific tissue datasets and then retrieve the raw gene expression value and inferred interaction confidences of the context‐specific gene relationships in each of the tissues.

## 4 DISCUSSION

Building context‐specific networks has been a widely studied topic in bioinformatics for decades. In this study, a top‐down strategy was used to build context‐specific networks both on transcriptome and interactome. The analysis in this paper is mainly focused on transcriptome, for the interactome data may include more genes with more noise, as the input PPI data are mostly predicted. In practice, the most widely used top‐down approach is PCA, but when using all the genes it cannot distinguish root tip from leaf tissues in the analysis. The advanced methods we adopted in the study overcome this limitation by identifying subsets of genes specific to each tissue. In this study, we reconstructed context‐specific networks using BicMix, a Bayesian biclustering method, C3D, a high‐order generalized singular value decomposition method on transcriptome level, and ActPro, an optimization method on interactome. All these methods have rigorous mathematical or statistical background, while BicMix and C3D can also provide statistical significance in results. From the computational perspective, BicMix needs huge computational resources for parameter estimation running long time MCMC in Bayesian inference, while C3D and ActPro are relatively faster in matrix manipulation. While BicMix and C3D have the common objective, BicMix aims to find only differences between tissues and C3D claims to find both differences and common modules. There is no guarantee for finding unique modules for specific tissues in the C3D model, as in our analysis. But users could still find useful tissue differences in C3D by ranking the confidences of the context‐specific interactions.

One observation is the difference between networks reconstructed from 6 combined tissues using all samples and 14 special tissues using sample subsets. Basically, one combined tissue reveals overall tissue specificity among all treatments and developments, and one specific tissue presents some specific properties standing out from the background. All these overall and detailed specificities are generally relevant only in the context of the datasets used for prediction. One example could be the analysis on the leaf. The leaf network based on all 15 leaf samples resulted in a very universal annotation enriched as protein folding that may exist in each tissue. However, focusing on only 3 leaf samples from symbiosis with rhizobium infection, the context‐specific network successfully captured genes enriched in photosynthesis. This latter result may reflect the relatively stronger effect on photosynthesis in plants that are limited for nitrogen, compared to control plants grown with various combined nitrogen sources. Comparing with the networks based on different contexts, it is encouraging that the analysis could reveal the interesting relationship between rhizobium–legume symbiosis and photosynthesis. In other words, the functional specificity arises from the context with the background and appropriate division of datasets, although, in practice, it may not be trivial to select the most appropriate inputs for different research purposes.

Exploring the network functionalities in the systems biology perspective may provide reasonable in silico results for plant researchers and molecular breading (Wang et al., 2013, 2015). There are still several limitations in our study. Due to inefficient annotation of Gene Ontology, pathway, and motifs in soybean, many results cannot be explained directly in functional analysis. Because of limitations in GO enrichment analysis, networks which are smaller in size do not have sufficient statistical power to deduce results for input with fewer than 10 genes with GO annotations. With the help of Arabidopsis GO enrichment analysis, all the significant motifs found in the upstream regions of TFs and their neighbors are highlighted in the transcription factor analysis, and most enriched motifs could get support from Arabidopsis motifs in the DAP motif database. Even with these enriched motifs, it may still be difficult to validate the occurrences and their functions directly in soybean. Besides, all the RNA‐Seq data in this study originate from the same data source and it is easy to handle these homogeneous data. However, the batch effect may exist if the data come from different sources, and suitable statistical models are still needed to address these batch effects in heterogeneous data. Additionally, as only context‐specific relationships vary among the same shared genes in C3D, it is hard to use any classical gene‐based annotation methods to analyze their results. Only results from BicMix are included and described in this manuscript, while other methods’ results are available on the website.

Considering the computing complexities, computing resources, and running time, it is not feasible to provide real‐time computing results using the methods mentioned above on the web server. Hence, SoyCSN adopted the approach to precompute results using high‐performance computing resources and store them in the database for easy access. Users can directly access these results from the website by querying the database efficiently. As more experiments in JGI are generated after this analysis, SoyCSN will continue to collect data, update methods, and provide in‐depth analyses.

## CONFLICT OF INTEREST

The authors declare no conflict of interest associated with the work described in this manuscript.
