# Supplementary material for: SoyCSN: Soybean context‐specific network analysis and prediction based on tissue‐specific transcriptome data
Source: Plant Direct. 2019 Sep 17;3(9):e00167. doi: 10.1002/pld3.167 (PMC6747016; doi:10.1002/pld3.167)
Supplement: Supplementary file 1 [file PLD3-3-e00167-s001.pdf]

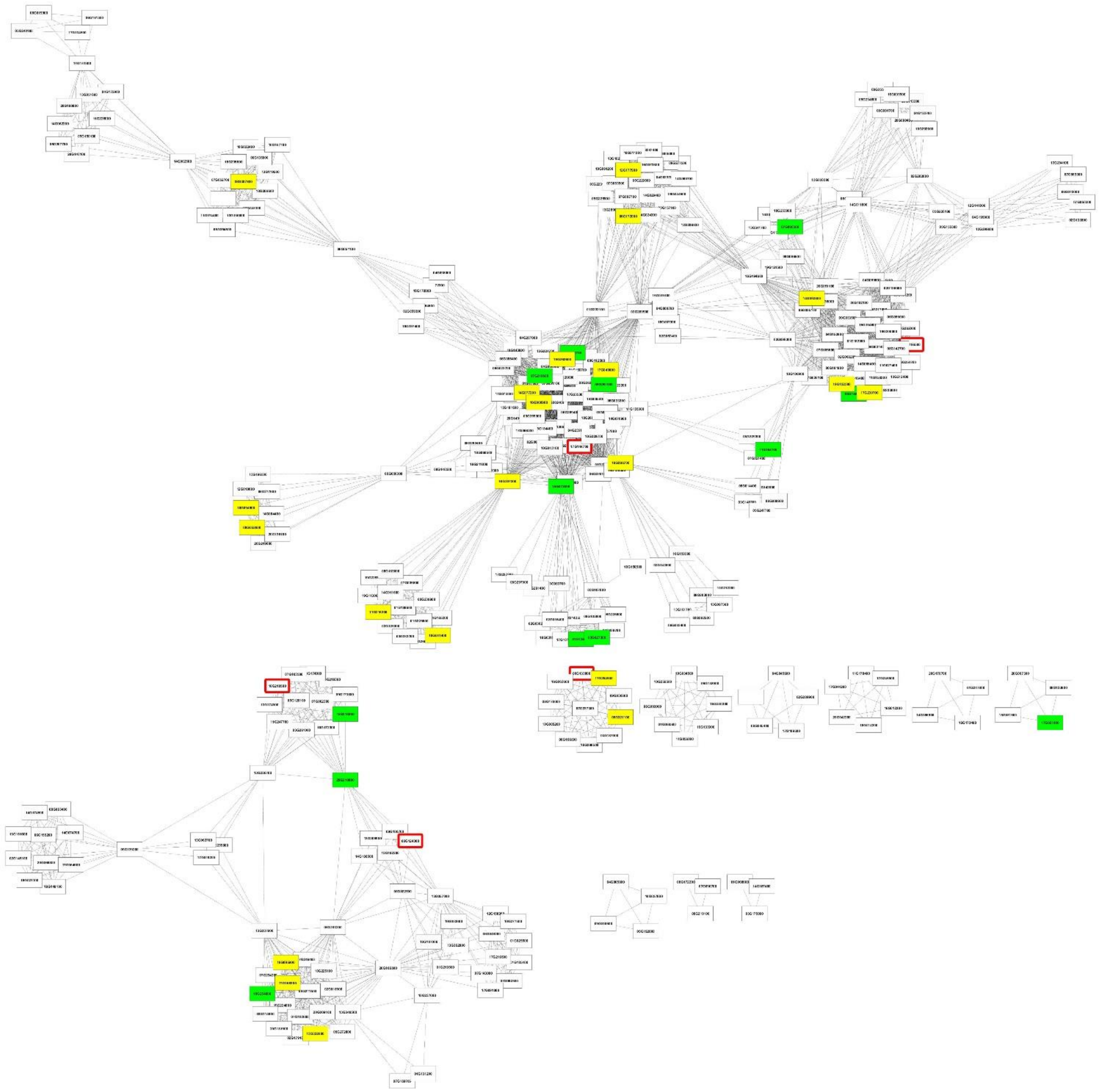

Figure.S1

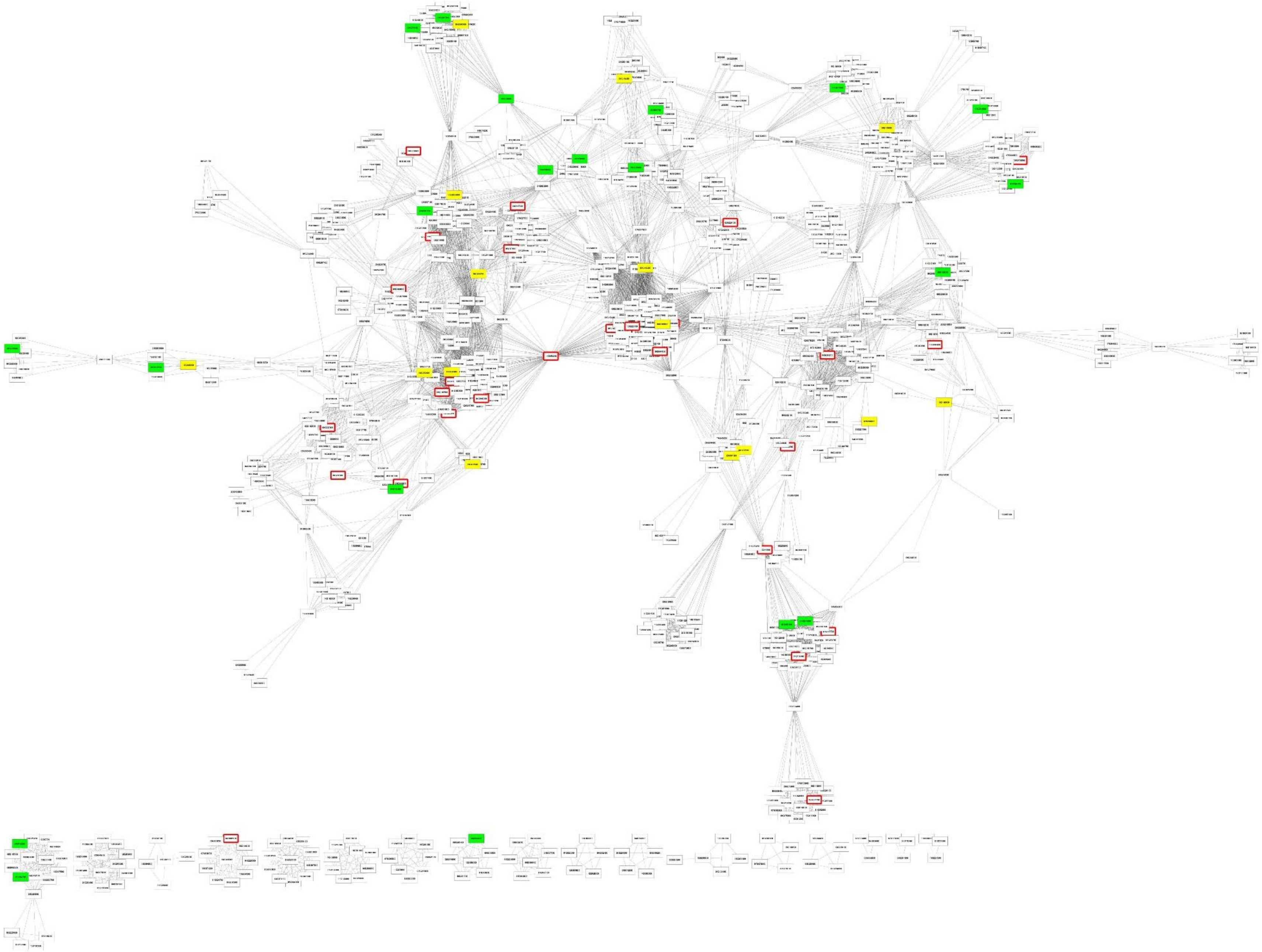

Figure.S2

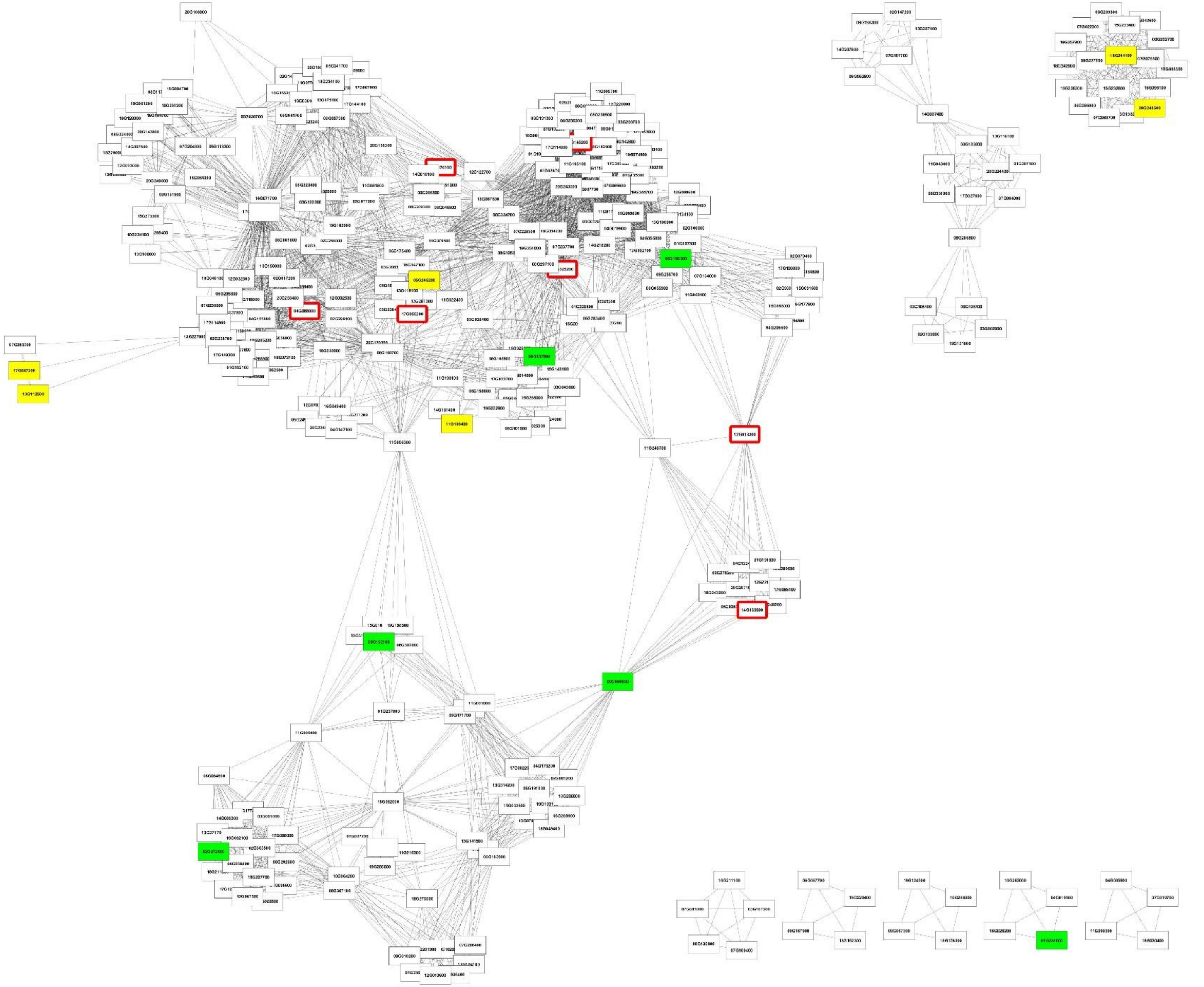

Figure.S3

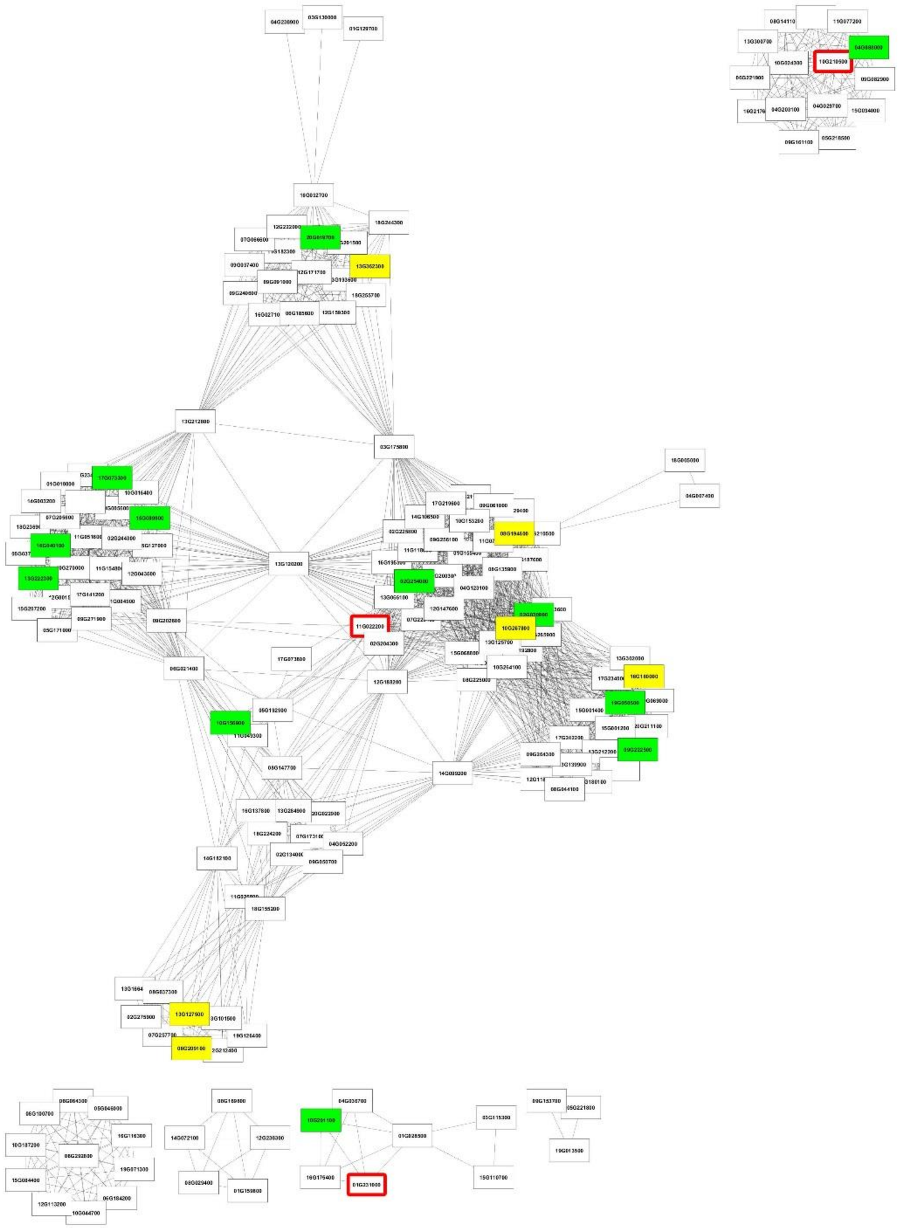

Figure.S4

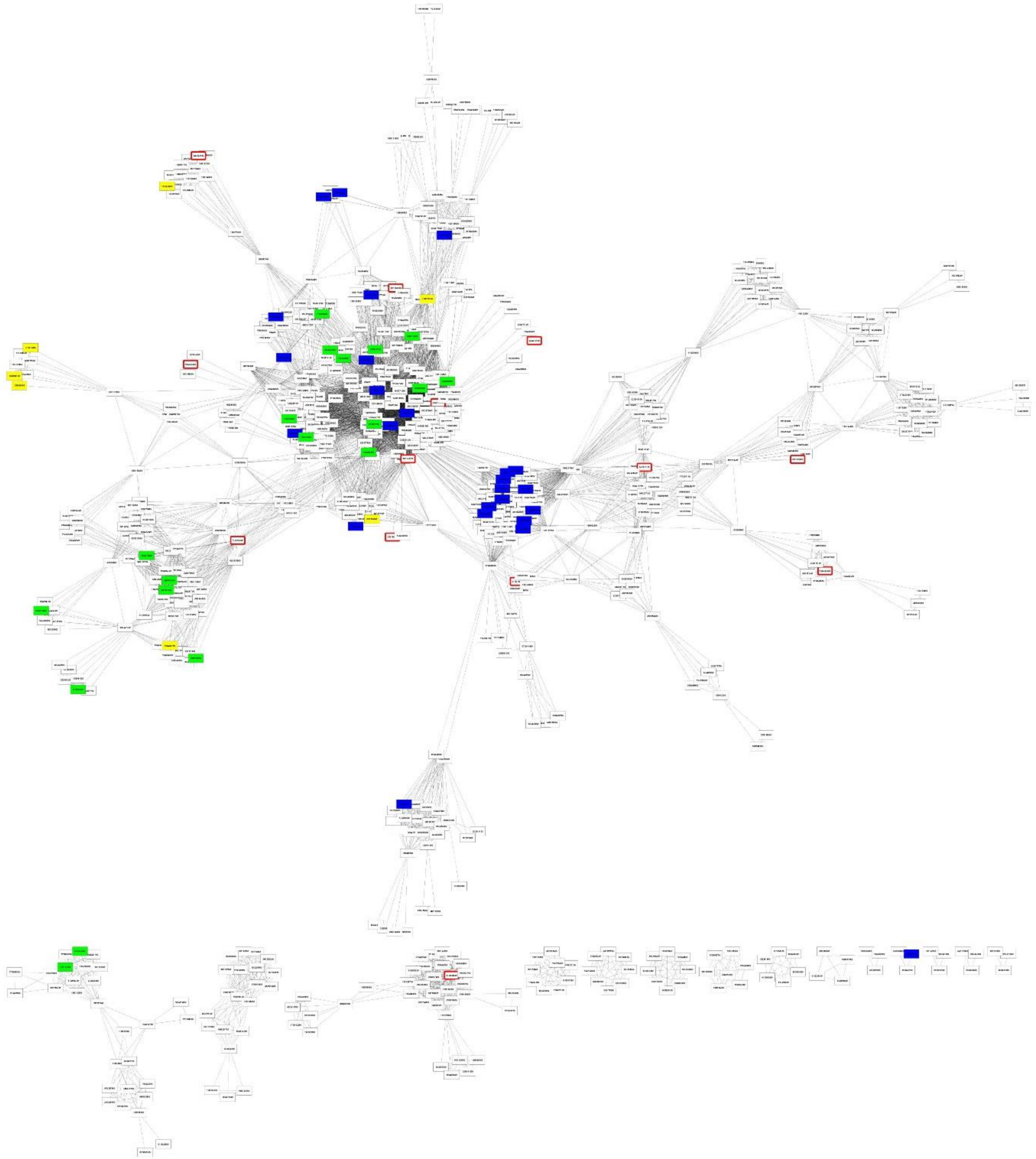

Figure.S5

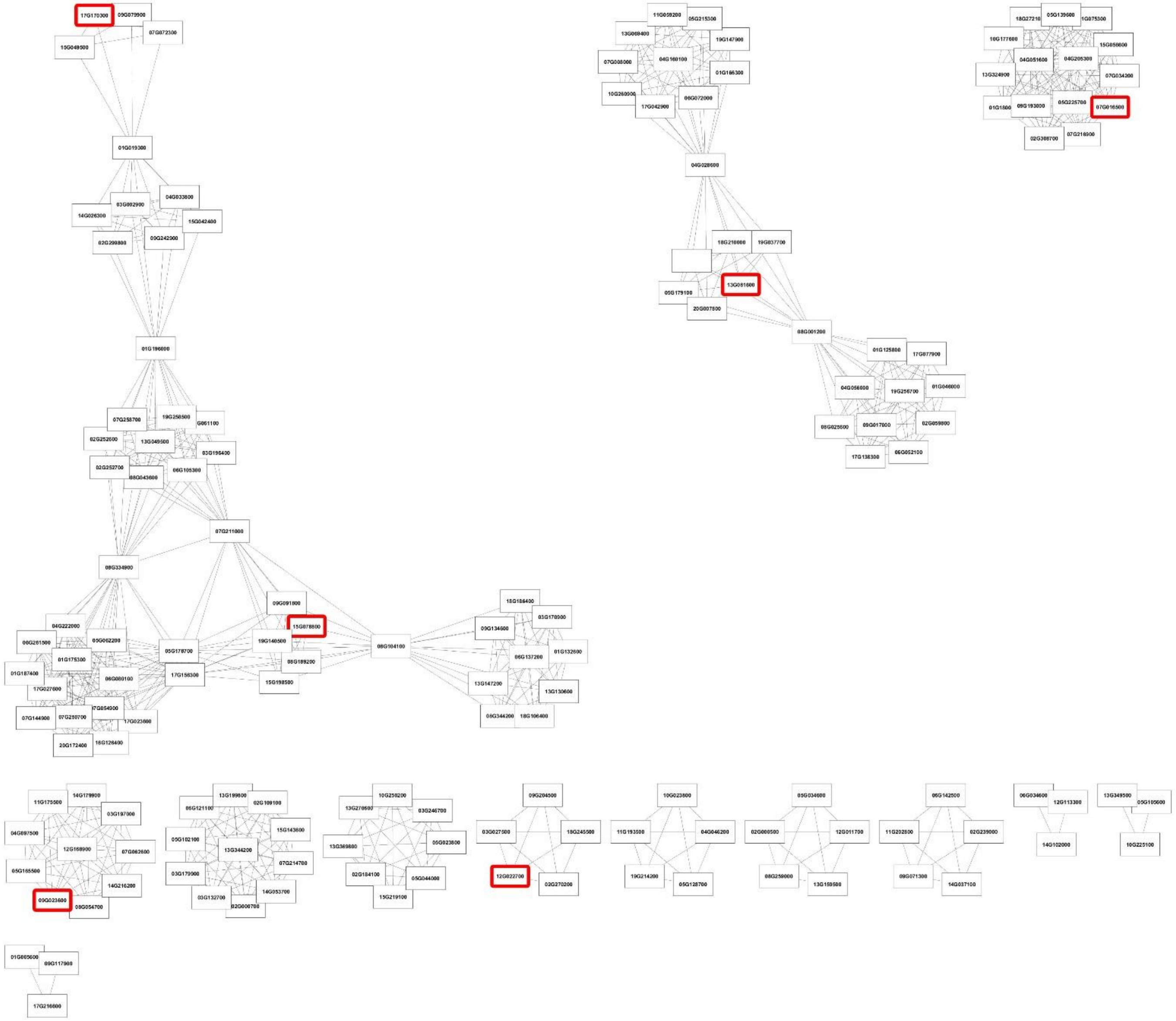

Figure.S6

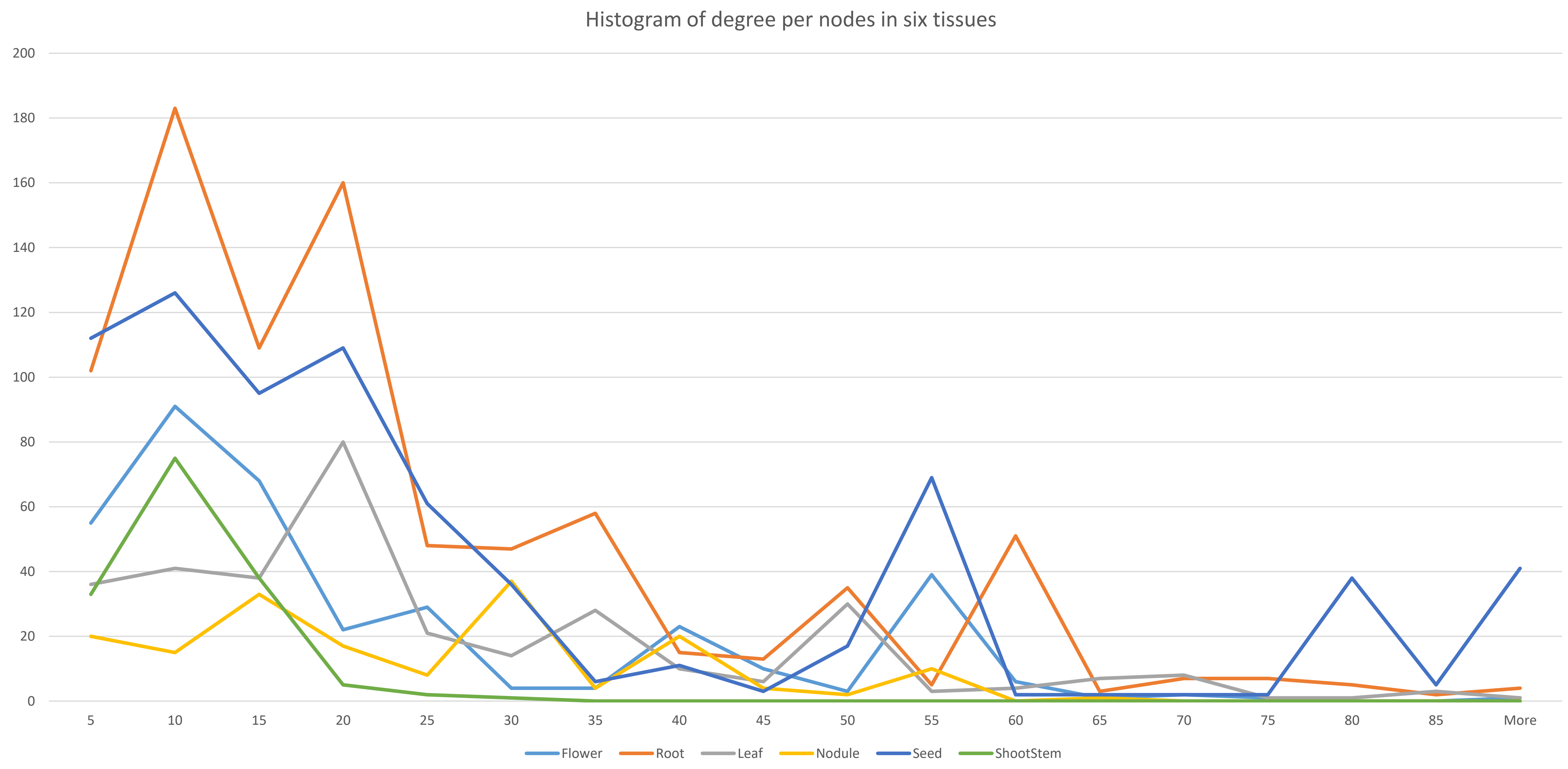
Histogram of degree per nodes in six tissues
200
180
160
140
120
100
80
60
40
20
0
5
10
15
20
25
30
35
40
45
50
55
60
65
70
75
80
85
More
Flower
Root
Leaf
Nodule
Seed
ShootStem

Figure.S7

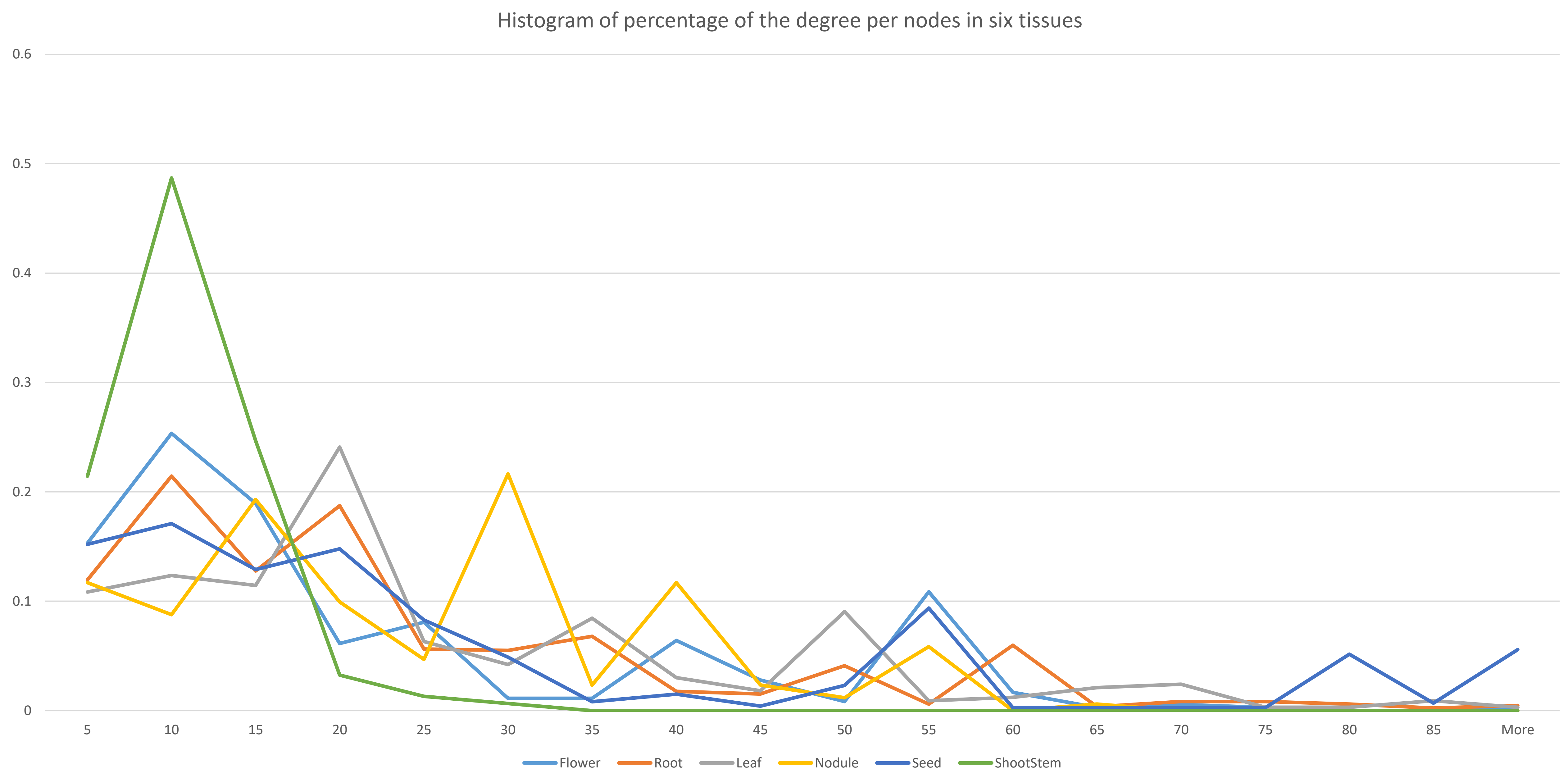
Histogram of percentage of the degree per nodes in six tissues
0.6
0.5
0.4
0.3
0.2
0.1
0
5
10
15
20
25
30
35
40
45
50
55
60
65
70
75
80
85
More
Flower
Root
Leaf
Nodule
Seed
ShootStem

Figure.S8

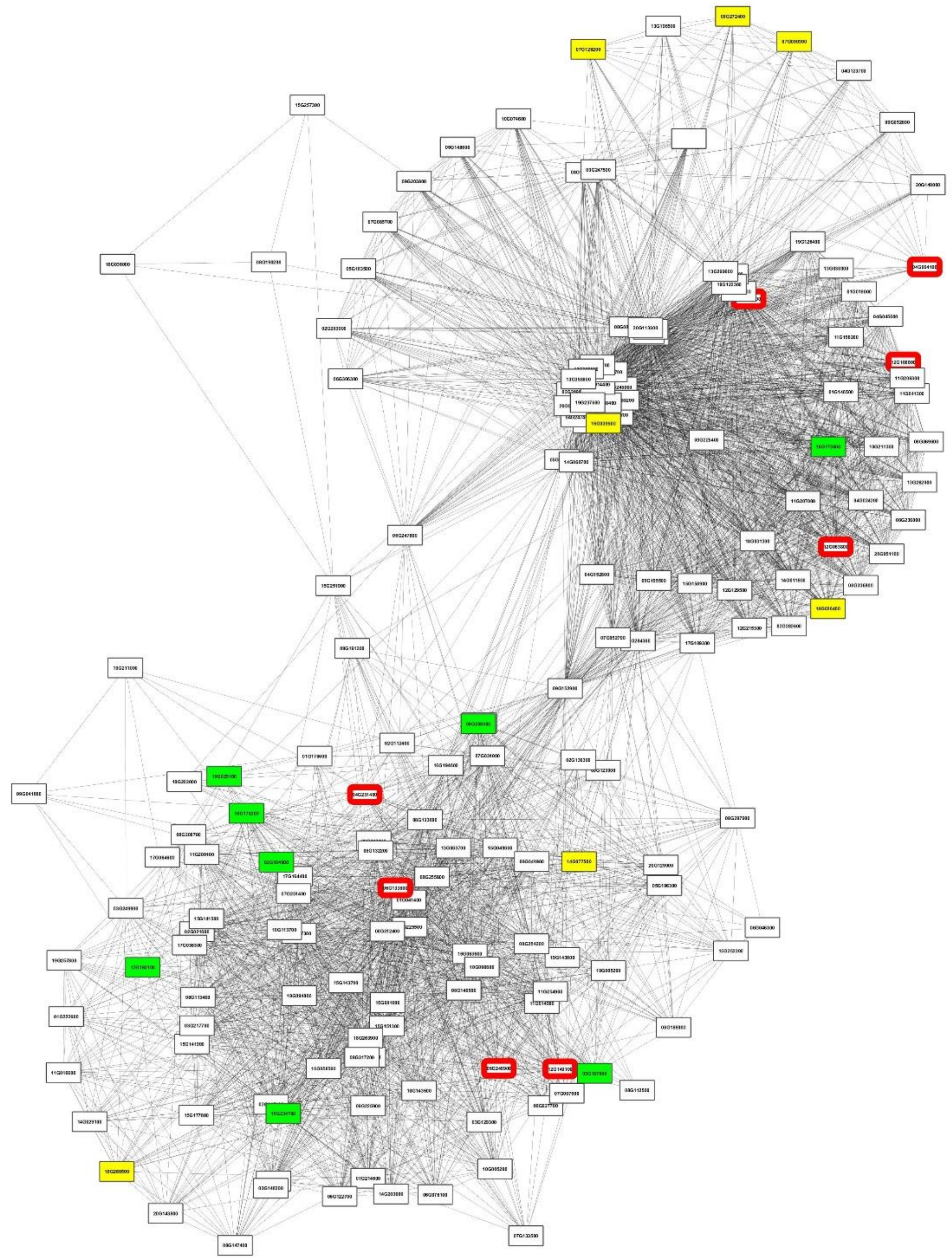

Figure.S9

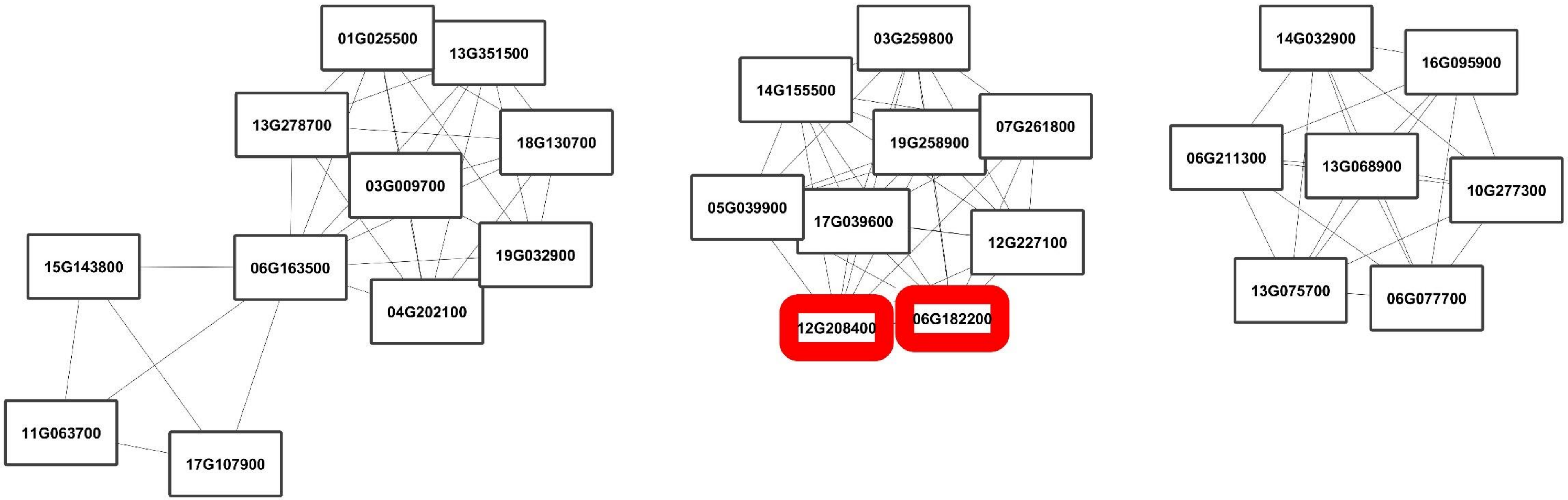
01G025500
13G351500
13G278700
18G130700
03G009700
15G143800
06G163500
19G032900
04G202100
11G063700
17G107900
03G259800
14G155500
07G261800
19G258900
05G039900
17G039600
12G227100
12G208400
06G182200
14G032900
16G095900
06G211300
13G068900
10G277300
13G075700
06G077700

Figure.S10

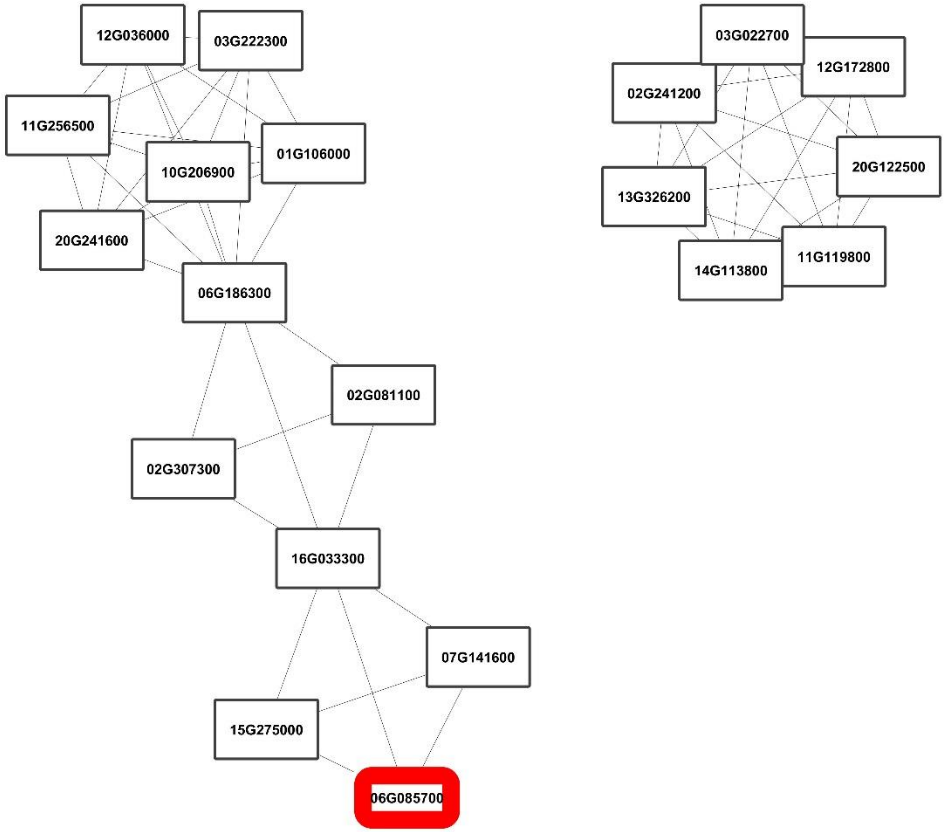
12G036000
03G222300
11G256500
10G206900
01G106000
20G241600
06G186300
02G081100
02G307300
16G033300
07G141600
15G275000
06G085700
03G022700
12G172800
02G241200
20G122500
13G326200
11G119800
14G113800

Figure.S11

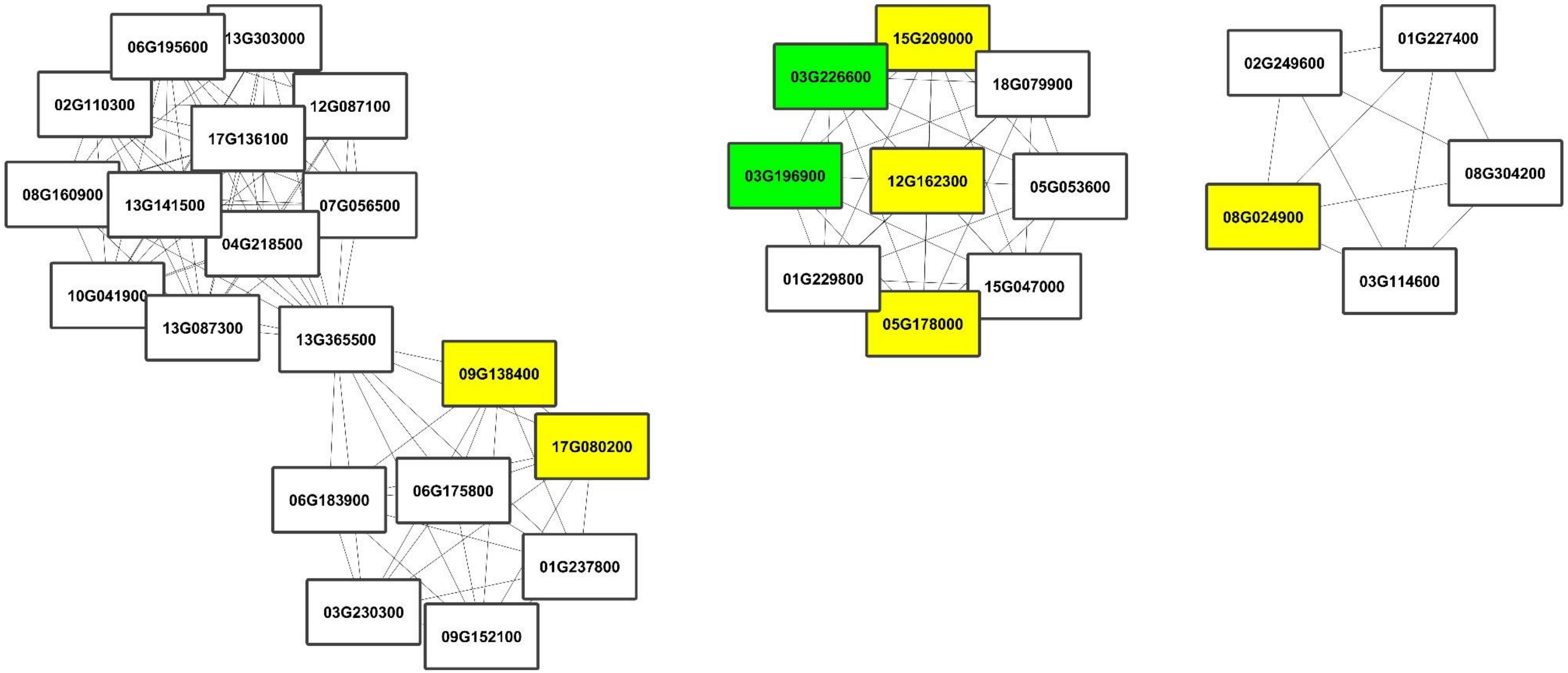
13G303000
06G195600
02G110300
12G087100
17G136100
08G160900
13G141500
07G056500
04G218500
10G041900
13G087300
13G365500
09G138400
17G080200
06G183900
06G175800
01G237800
03G230300
09G152100
15G209000
03G226600
18G079900
03G196900
12G162300
05G053600
01G229800
15G047000
05G178000
01G227400
02G249600
08G304200
08G024900
03G114600

Figure.S12

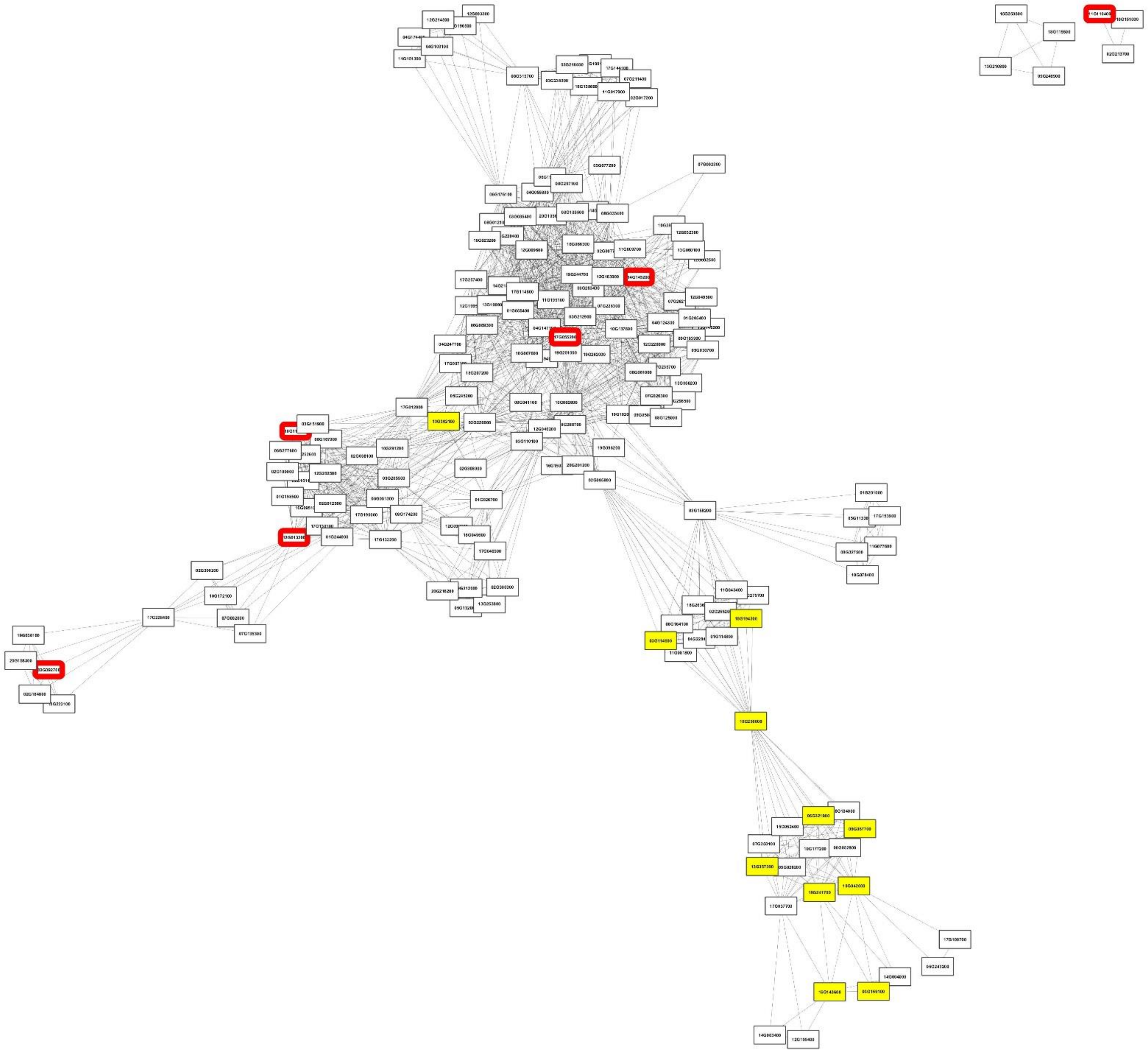

Figure.S13

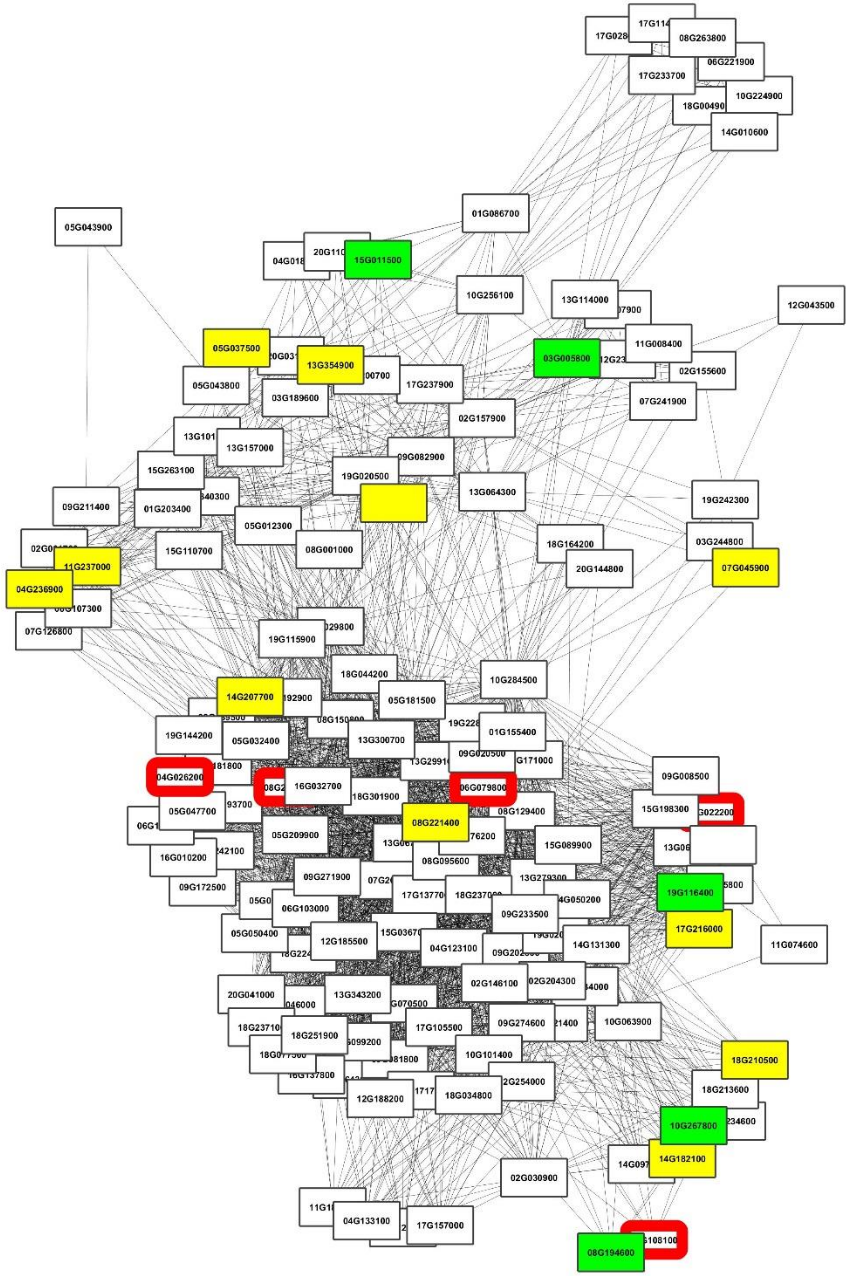

08G263800
06G221900
17G233700
10G224900
14G010600
05G043900
01G086700
15G011500
10G256100
13G114000
12G043500
11G008400
03G005800
02G155600
07G241900
05G037500
13G354900
05G043800
03G189600
17G237900
02G157900
13G157000
15G263100
09G082900
19G020500
13G064300
09G211400
01G203400
05G012300
19G242300
18G164200
03G244800
15G110700
08G001000
20G144800
11G237000
07G045900
04G236900
07G126800
19G115900
18G044200
10G284500
14G207700
05G181500
19G144200
05G032400
13G300700
01G155400
04G026200
16G032700
06G079800
09G008500
18G301900
08G129400
15G198300
05G047700
08G221400
05G209900
15G089900
16G010200
08G095600
09G271900
09G172500
19G116400
06G103000
18G237000
09G233500
17G216000
05G050400
12G185500
04G123100
14G131300
11G074600
20G041000
02G146100
02G204300
13G343200
17G105500
09G274600
10G063900
18G251900
10G101400
18G210500
18G034800
12G188200
18G213600
10G267800
14G182100
02G030900
04G133100
17G157000
08G194600

Figure.S14

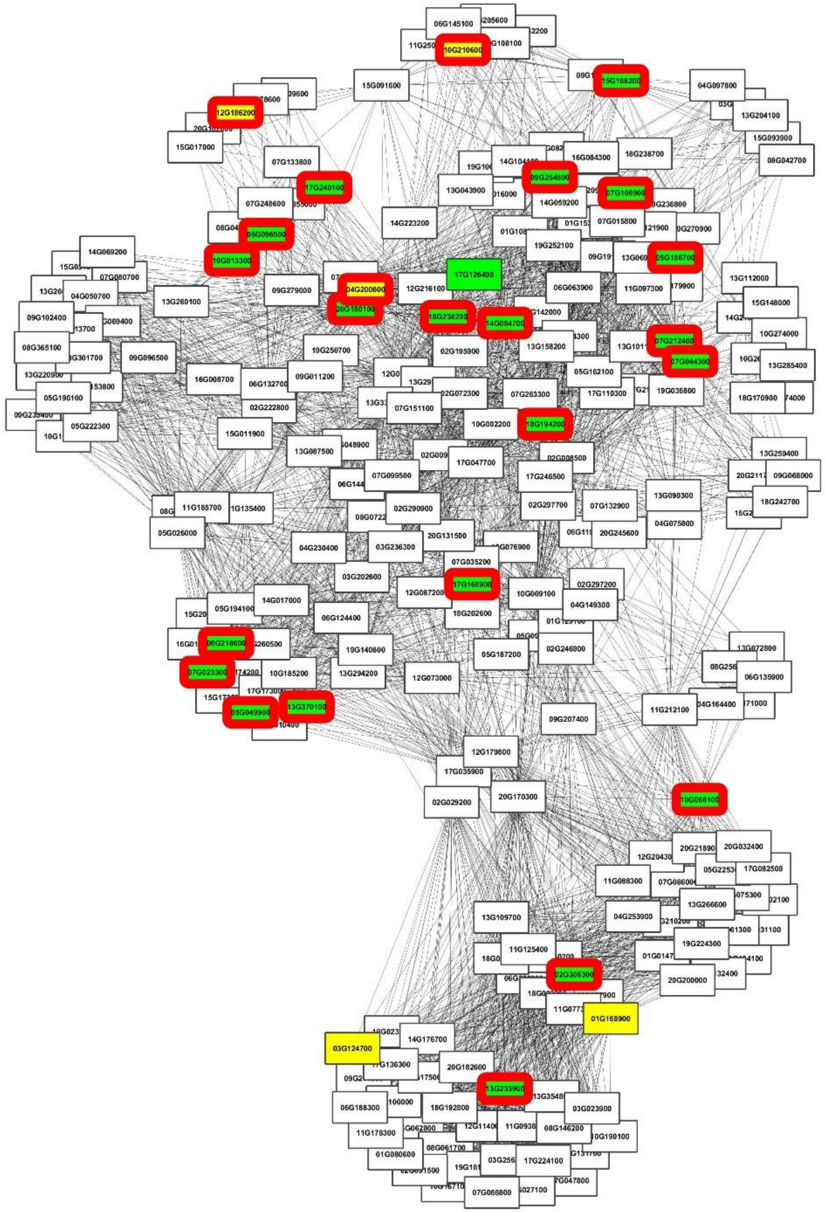

Figure.S15

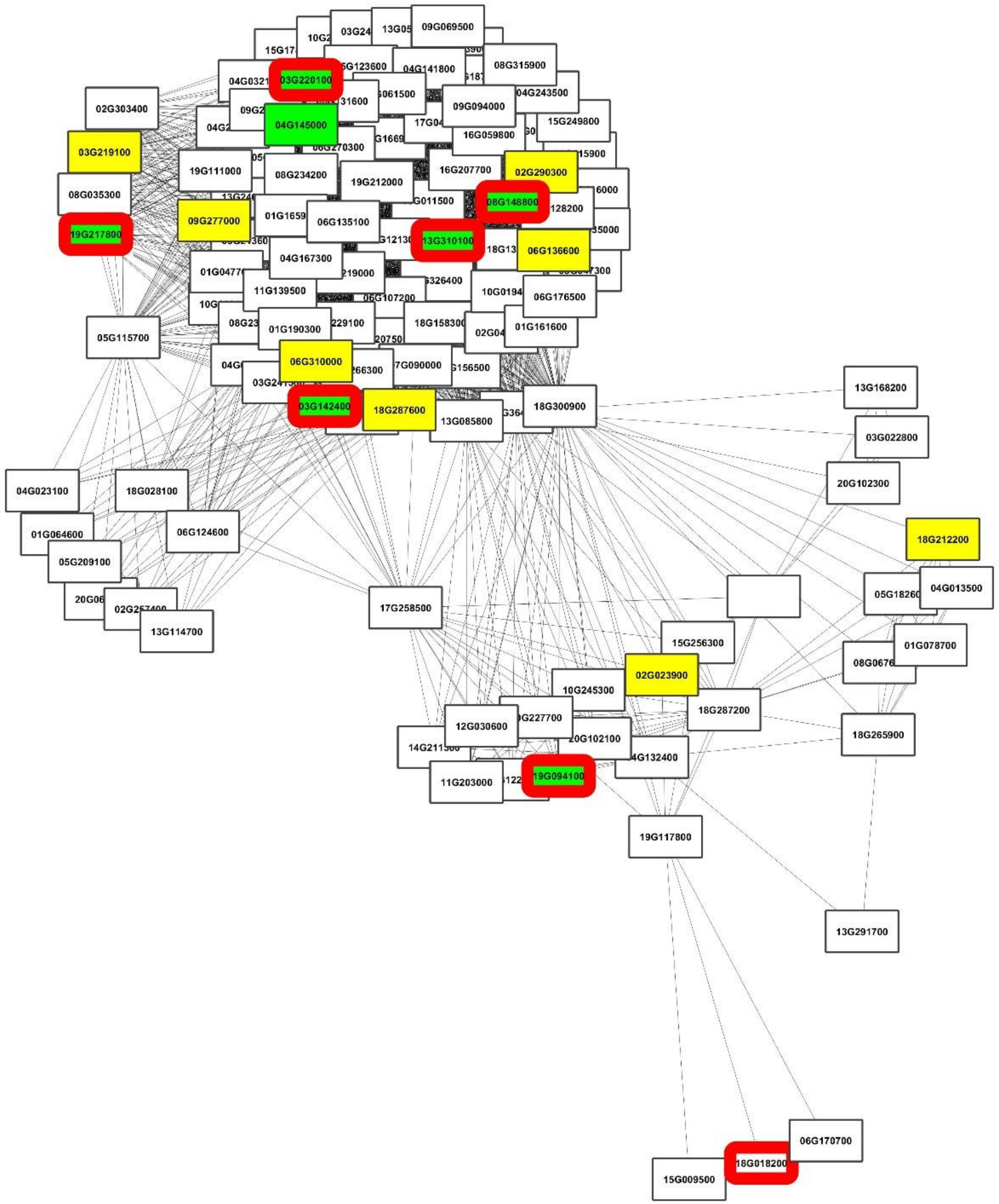

Figure.S16

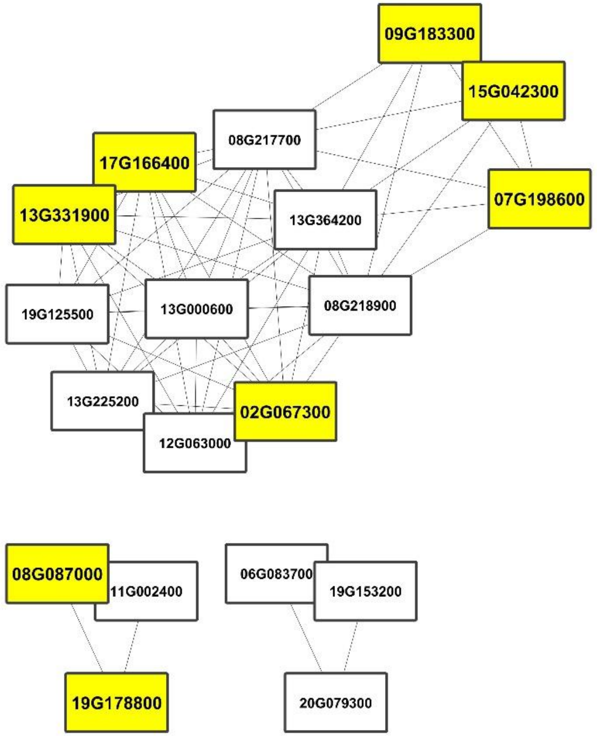
09G183300
15G042300
08G217700
17G166400
13G331900
07G198600
13G364200
19G125500
13G000600
08G218900
13G225200
02G067300
12G063000
08G087000
11G002400
06G083700
19G153200
19G178800
20G079300

Figure.S17

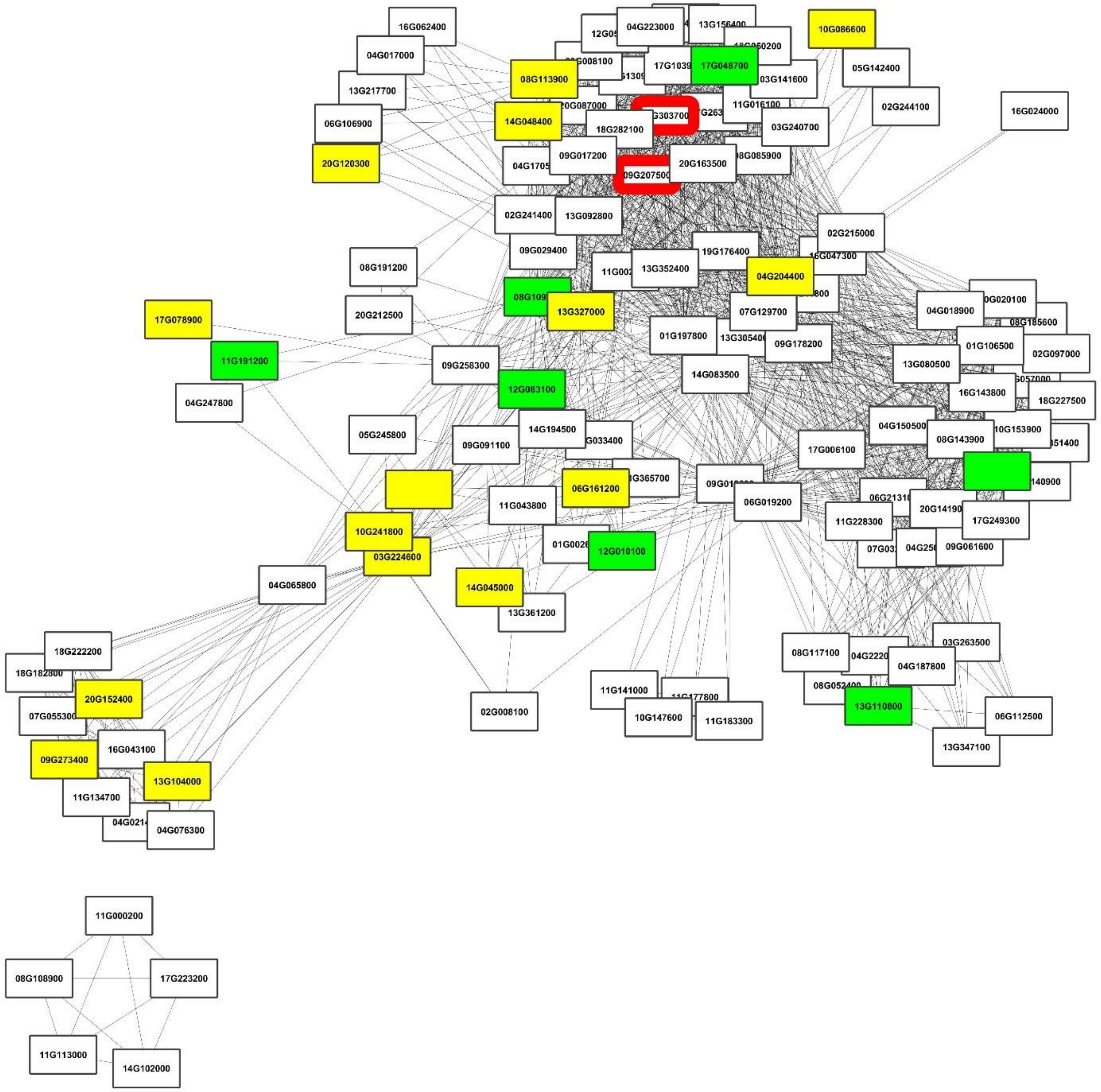
16G062400
04G017000
13G217700
06G106900
20G120300
08G113900
14G048400
20G087000
04G223000
13G156400
10G086600
17G048700
03G141600
05G142400
02G244100
16G024000
11G016100
03G240700
18G282100
09G017200
20G163500
02G241400
13G092800
09G029400
02G215000
19G176400
13G352400
04G204400
08G191200
20G212500
13G327000
07G129700
04G018900
01G197800
09G178200
01G106500
02G097000
17G078900
11G191200
09G258300
12G083100
14G083500
13G080500
16G143800
18G227500
04G247800
05G245800
14G194500
09G091100
04G150500
08G143900
17G006100
06G161200
11G043800
06G019200
11G228300
17G249300
09G061600
10G241800
03G224600
12G010100
04G065800
14G045000
13G361200
18G222200
18G182800
20G152400
07G055300
03G263500
08G117100
04G187800
11G141000
10G147600
11G183300
13G110800
06G112500
13G347100
02G008100
16G043100
09G273400
13G104000
11G134700
04G076300
11G000200
08G108900
17G223200
11G113000
14G102000

Figure.S18

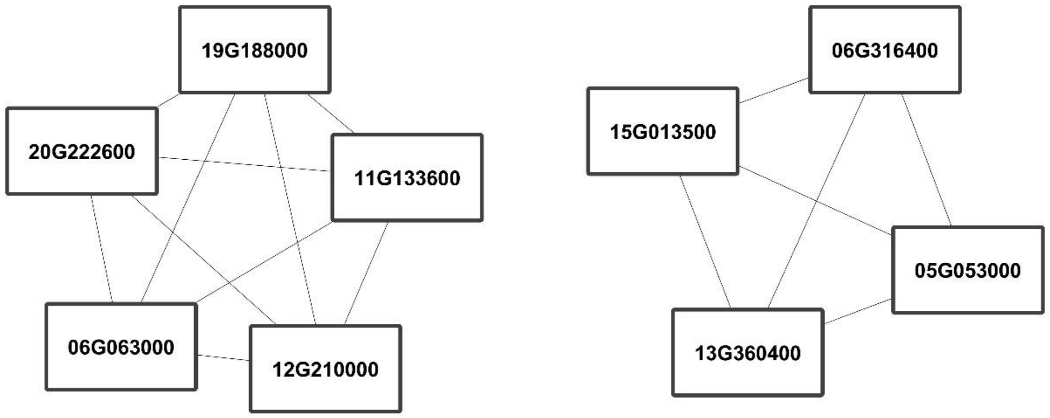
19G188000
20G222600
11G133600
06G063000
12G210000
06G316400
15G013500
05G053000
13G360400

Figure.S19

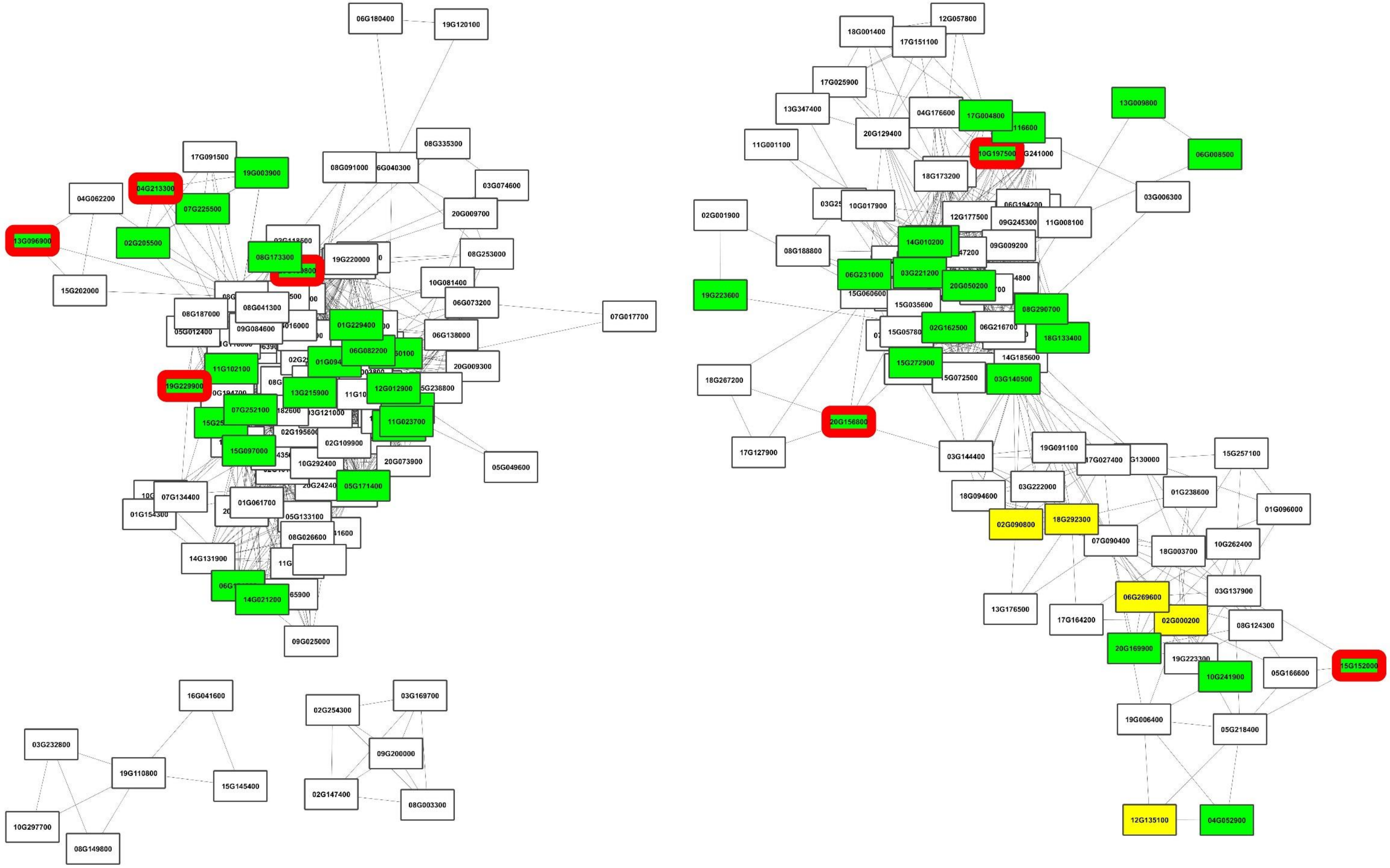
06G180400
19G120100
08G335300
17G091500
19G003900
08G091000
03G074600
04G062200
04G213300
07G225500
20G009700
13G096900
02G205500
08G173300
19G220000
08G253000
15G202000
10G081400
08G187000
08G041300
06G073200
07G017700
05G012400
09G084600
01G229400
06G138000
06G082200
11G102100
20G009300
19G229900
13G215900
12G012900
07G252100
11G023700
02G109900
15G097000
10G292400
20G073900
05G049600
05G171400
07G134400
01G061700
01G154300
05G133100
08G026600
14G131900
14G021200
09G025000
16G041600
03G169700
02G254300
03G232800
09G200000
19G110800
15G145400
02G147400
08G003300
10G297700
08G149800
12G057800
18G001400
17G151100
17G025900
13G009800
13G347400
04G176600
17G004800
20G129400
11G001100
10G197500
06G008500
18G173200
03G006300
10G017900
02G001900
12G177500
09G245300
11G008100
08G188800
14G010200
09G009200
06G231000
03G221200
20G050200
19G223600
15G060600
15G035600
08G290700
02G162500
06G216700
18G133400
14G185600
15G272900
18G267200
15G072500
03G140500
20G156800
17G127900
03G144400
19G091100
15G257100
18G094600
03G222000
01G238600
01G096000
02G090800
18G292300
07G090400
18G003700
10G262400
03G137900
13G176500
06G269600
17G164200
02G000200
08G124300
20G169900
19G223300
10G241900
05G166600
15G152000
19G006400
05G218400
12G135100
04G052900

Figure.S20

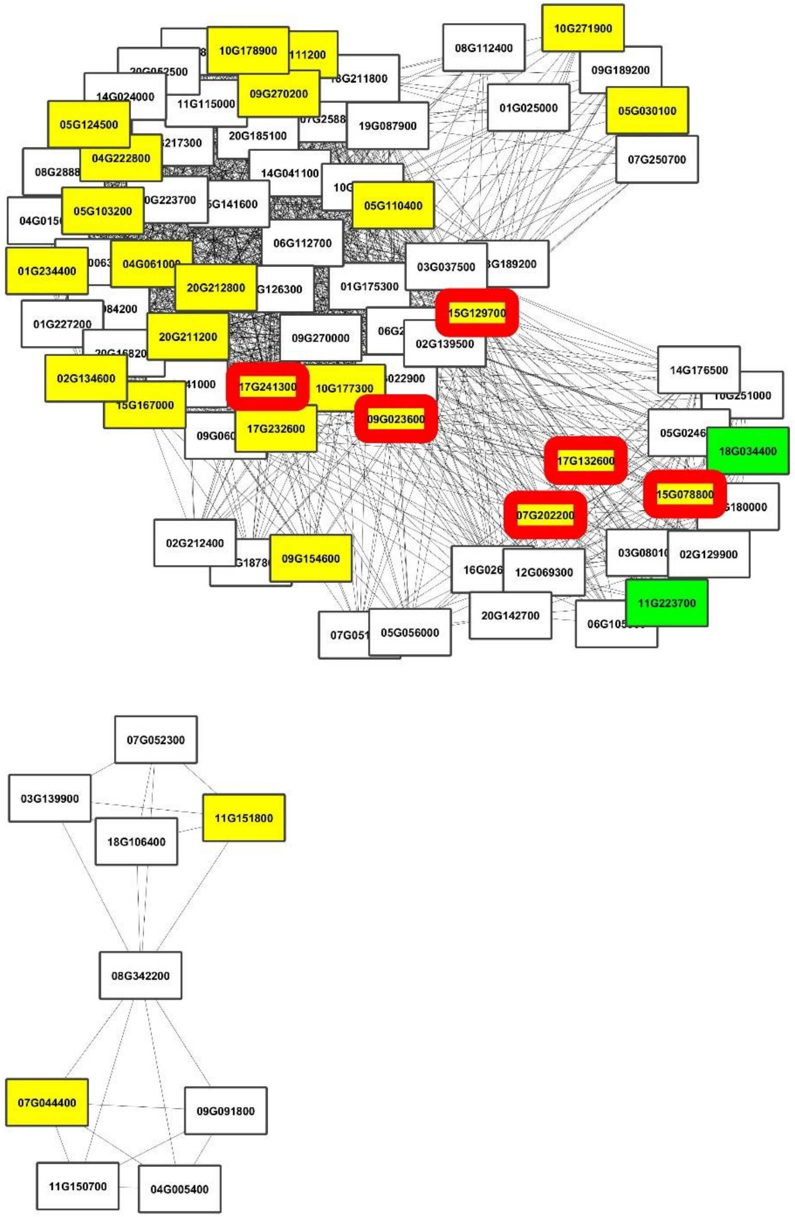

10G178900
09G270200
14G024000
11G115000
05G124500
04G222800
20G185100
14G041100
19G087900
05G103200
06G112700
01G234400
20G212800
01G175300
03G037500
01G227200
20G211200
09G270000
02G139500
02G134600
15G167000
17G241300
10G177300
09G023600
17G232600
08G112400
10G271900
09G189200
01G025000
05G030100
07G250700
05G110400
15G129700
14G176500
18G034400
17G132600
15G078800
07G202200
02G129900
12G069300
20G142700
11G223700
02G212400
09G154600
05G056000
07G052300
03G139900
11G151800
18G106400
08G342200
07G044400
09G091800
11G150700
04G005400

Figure.S21

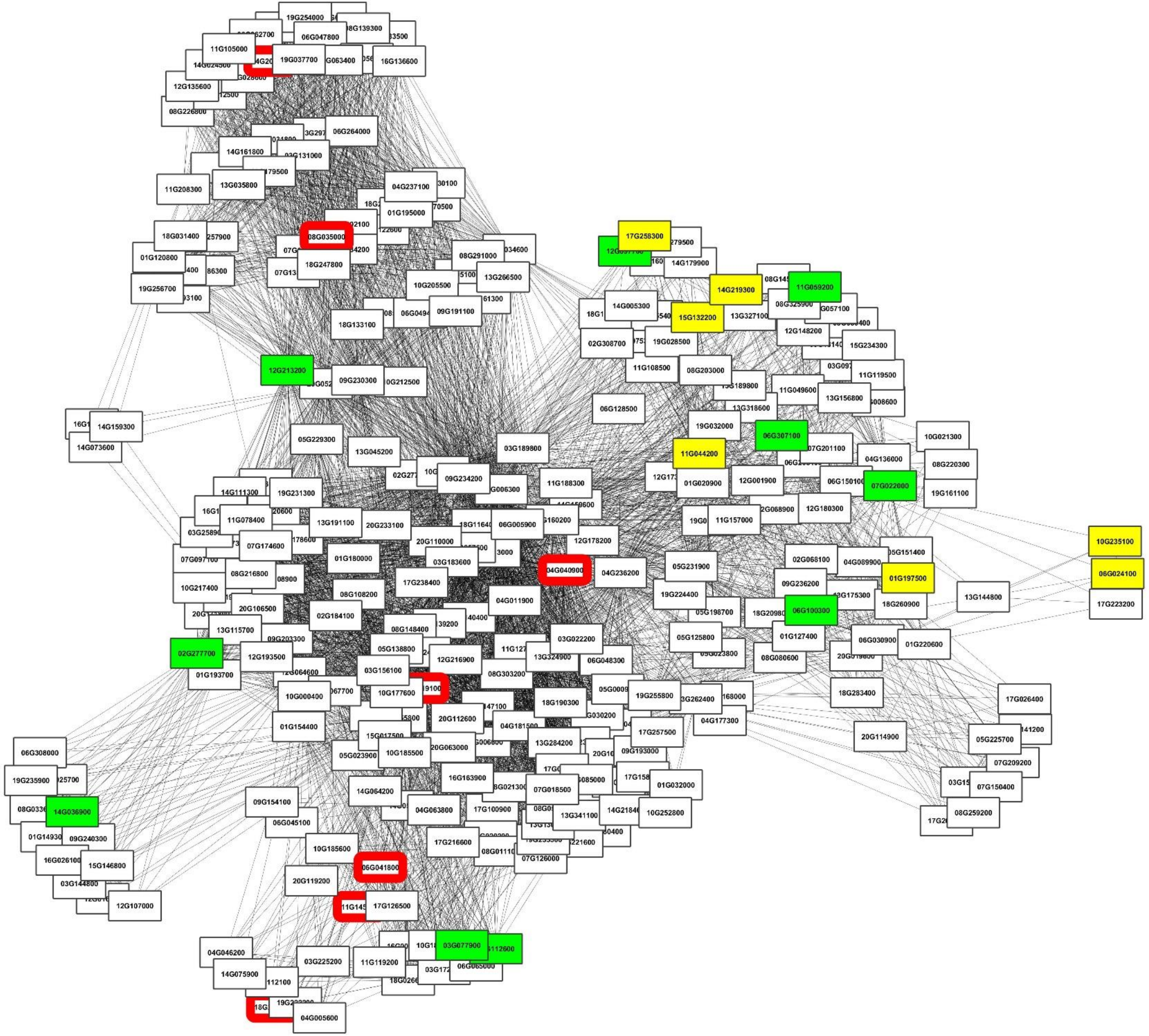
19G254000
06G047800
08G139300
11G105000
19G037700
16G136600
12G135600
08G226800
06G264000
14G161800
03G131000
11G208300
13G035800
04G237100
01G195000
08G035000
18G031400
01G120800
18G247800
08G291000
13G266500
19G256700
10G205500
09G191100
18G133100
12G213200
09G230300
17G258300
14G179900
14G219300
11G059200
14G005300
15G132200
13G327100
08G325900
12G148200
15G234300
02G308700
19G028500
11G108500
08G203000
11G119500
11G049600
13G156800
13G318600
06G128500
19G032000
06G307100
10G021300
11G044200
07G201100
04G136000
08G220300
16G159300
14G073600
05G229300
13G045200
03G189800
09G234200
11G188300
01G020900
12G001900
07G022000
19G161100
14G111300
19G231300
11G078400
13G191100
20G233100
06G005900
11G157000
12G180300
07G174600
20G110000
12G178200
01G180000
03G183600
10G235100
07G097100
08G216800
04G040900
04G236200
05G231900
02G068100
04G089900
05G151400
01G197500
06G024100
10G217400
17G238400
08G108200
04G011900
19G224400
09G236200
13G175300
13G144800
17G223200
20G106500
02G184100
05G198700
06G100300
18G260900
13G115700
08G148400
03G022200
05G125800
01G127400
06G030900
01G220600
02G277700
09G203300
05G138800
12G216900
13G324900
06G048300
08G080600
12G193500
03G156100
08G303200
01G193700
10G000400
10G177600
18G190300
19G255800
18G283400
17G026400
20G112600
04G177300
01G154400
04G181500
17G257500
20G114900
05G225700
06G308000
13G284200
07G209200
15G017500
10G185500
20G063000
09G193000
05G023900
19G235900
16G163900
07G150400
14G036900
14G064200
01G032000
08G259200
07G018500
09G154100
04G063800
17G100900
14G21840
10G252800
06G045100
13G341100
09G240300
17G216600
10G185600
16G026100
15G146800
06G041800
07G126000
20G119200
03G144800
12G107000
17G126500
03G077900
04G046200
03G225200
11G119200
14G075900
06G065000
19G222300
04G005600

Figure.S22

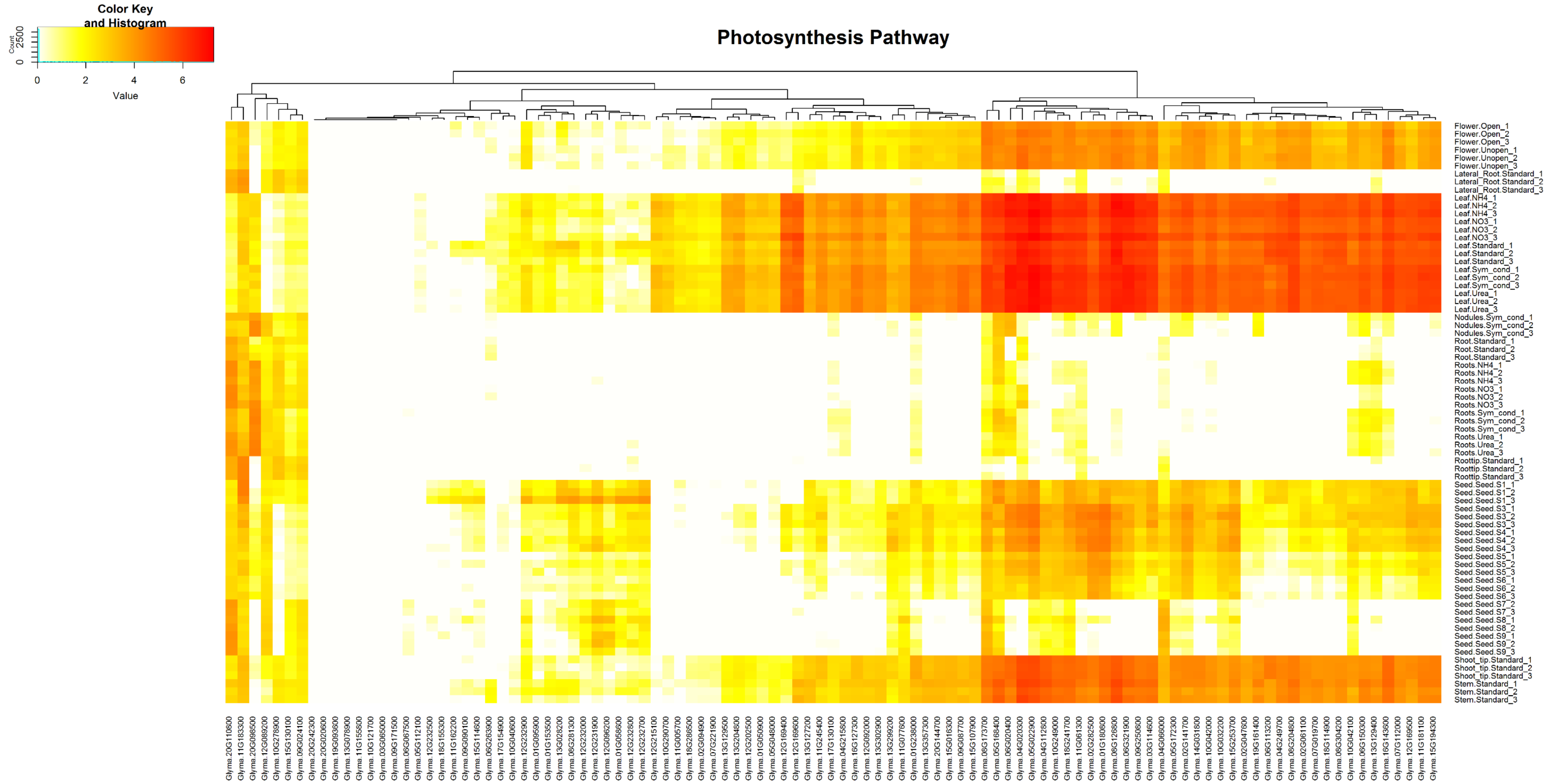
Color Key
and Histogram
Count
0
2500
0
2
4
6
Value
Photosynthesis Pathway
Flower.Open_1
Flower.Open_2
Flower.Open_3
Flower.Unopen_1
Flower.Unopen_2
Flower.Unopen_3
Lateral_Root.Standard_1
Lateral_Root.Standard_2
Lateral_Root.Standard_3
Leaf.NH4_1
Leaf.NH4_2
Leaf.NH4_3
Leaf.NO3_1
Leaf.NO3_2
Leaf.NO3_3
Leaf.Standard_1
Leaf.Standard_2
Leaf.Standard_3
Leaf.Sym_cond_1
Leaf.Sym_cond_2
Leaf.Sym_cond_3
Leaf.Urea_1
Leaf.Urea_2
Leaf.Urea_3
Nodules.Sym_cond_1
Nodules.Sym_cond_2
Nodules.Sym_cond_3
Root.Standard_1
Root.Standard_2
Root.Standard_3
Roots.NH4_1
Roots.NH4_2
Roots.NH4_3
Roots.NO3_1
Roots.NO3_2
Roots.NO3_3
Roots.Sym_cond_1
Roots.Sym_cond_2
Roots.Sym_cond_3
Roots.Urea_1
Roots.Urea_2
Roots.Urea_3
Roottip.Standard_1
Roottip.Standard_2
Roottip.Standard_3
Seed.Seed.S1_1
Seed.Seed.S1_2
Seed.Seed.S1_3
Seed.Seed.S3_1
Seed.Seed.S3_2
Seed.Seed.S3_3
Seed.Seed.S4_1
Seed.Seed.S4_2
Seed.Seed.S4_3
Seed.Seed.S5_1
Seed.Seed.S5_2
Seed.Seed.S5_3
Seed.Seed.S6_1
Seed.Seed.S6_2
Seed.Seed.S6_3
Seed.Seed.S7_2
Seed.Seed.S7_3
Seed.Seed.S8_1
Seed.Seed.S8_2
Seed.Seed.S9_1
Seed.Seed.S9_2
Seed.Seed.S9_3
Shoot_tip.Standard_1
Shoot_tip.Standard_2
Shoot_tip.Standard_3
Stem.Standard_1
Stem.Standard_2
Stem.Standard_3

Figure.S23

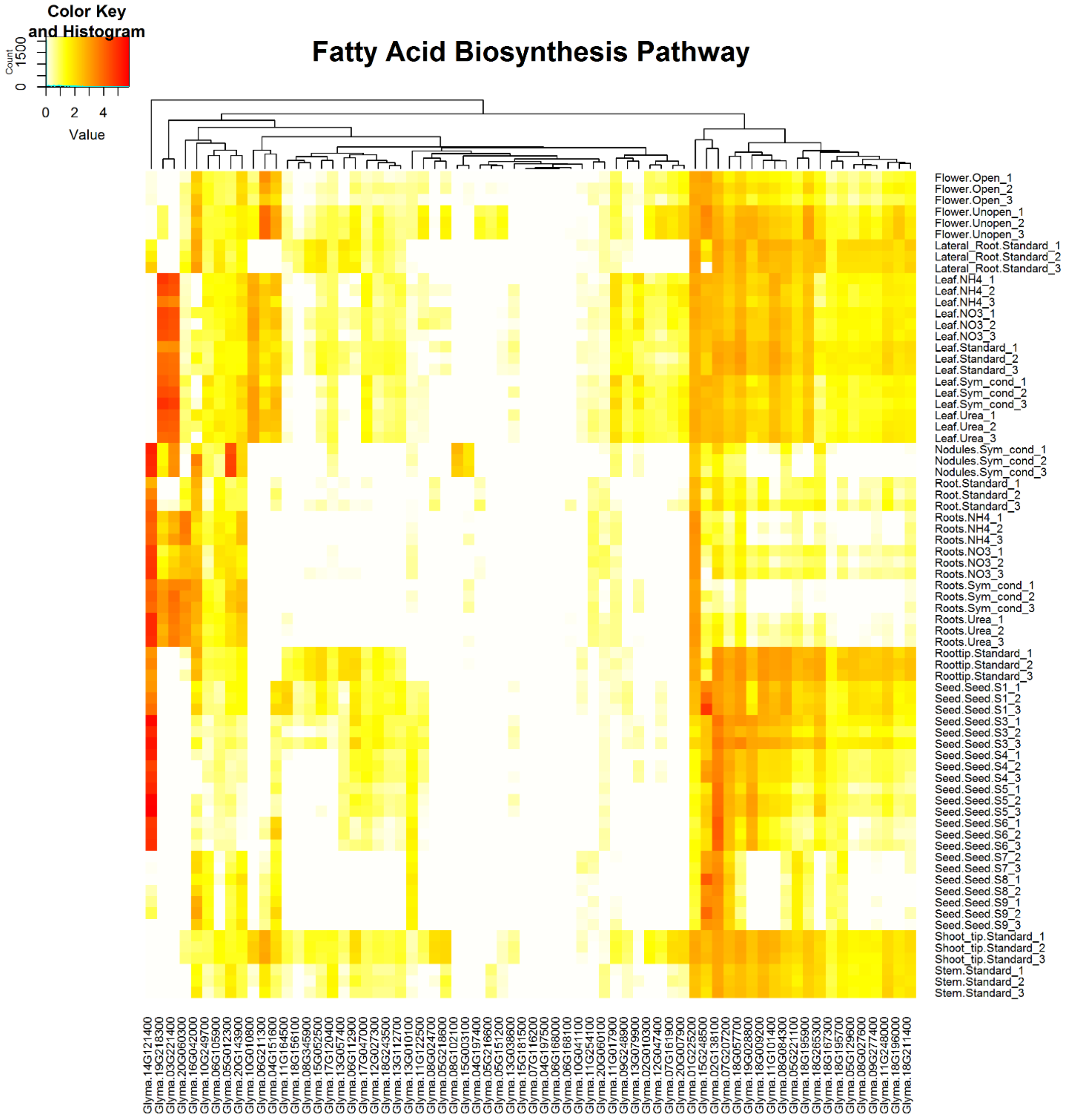
Color Key
and Histogram
Count
0
1500
0
2
4
Value
Fatty Acid Biosynthesis Pathway
Flower.Open_1
Flower.Open_2
Flower.Open_3
Flower.Unopen_1
Flower.Unopen_2
Flower.Unopen_3
Lateral_Root.Standard_1
Lateral_Root.Standard_2
Lateral_Root.Standard_3
Leaf.NH4_1
Leaf.NH4_2
Leaf.NH4_3
Leaf.NO3_1
Leaf.NO3_2
Leaf.NO3_3
Leaf.Standard_1
Leaf.Standard_2
Leaf.Standard_3
Leaf.Sym_cond_1
Leaf.Sym_cond_2
Leaf.Sym_cond_3
Leaf.Urea_1
Leaf.Urea_2
Leaf.Urea_3
Nodules.Sym_cond_1
Nodules.Sym_cond_2
Nodules.Sym_cond_3
Root.Standard_1
Root.Standard_2
Root.Standard_3
Roots.NH4_1
Roots.NH4_2
Roots.NH4_3
Roots.NO3_1
Roots.NO3_2
Roots.NO3_3
Roots.Sym_cond_1
Roots.Sym_cond_2
Roots.Sym_cond_3
Roots.Urea_1
Roots.Urea_2
Roots.Urea_3
Roottip.Standard_1
Roottip.Standard_2
Roottip.Standard_3
Seed.Seed.S1_1
Seed.Seed.S1_2
Seed.Seed.S1_3
Seed.Seed.S3_1
Seed.Seed.S3_2
Seed.Seed.S3_3
Seed.Seed.S4_1
Seed.Seed.S4_2
Seed.Seed.S4_3
Seed.Seed.S5_1
Seed.Seed.S5_2
Seed.Seed.S5_3
Seed.Seed.S6_1
Seed.Seed.S6_2
Seed.Seed.S6_3
Seed.Seed.S7_2
Seed.Seed.S7_3
Seed.Seed.S8_1
Seed.Seed.S8_2
Seed.Seed.S9_1
Seed.Seed.S9_2
Seed.Seed.S9_3
Shoot_tip.Standard_1
Shoot_tip.Standard_2
Shoot_tip.Standard_3
Stem.Standard_1
Stem.Standard_2
Stem.Standard_3
Glyma.14G121400
Glyma.19G218300
Glyma.03G221400
Glyma.20G060300
Glyma.16G042000
Glyma.10G249700
Glyma.06G105900
Glyma.05G012300
Glyma.20G143900
Glyma.10G010800
Glyma.06G211300
Glyma.04G151600
Glyma.11G164500
Glyma.18G156100
Glyma.08G345900
Glyma.15G052500
Glyma.17G120400
Glyma.13G057400
Glyma.06G112900
Glyma.17G047000
Glyma.12G027300
Glyma.18G243500
Glyma.13G112700
Glyma.13G010100
Glyma.11G122500
Glyma.08G024700
Glyma.05G218600
Glyma.08G102100
Glyma.15G003100
Glyma.04G197400
Glyma.05G216600
Glyma.05G151200
Glyma.13G038600
Glyma.15G181500
Glyma.07G116200
Glyma.04G197500
Glyma.06G168000
Glyma.06G168100
Glyma.10G041100
Glyma.11G254100
Glyma.20G060100
Glyma.11G017900
Glyma.09G248900
Glyma.13G079900
Glyma.02G010300
Glyma.12G047400
Glyma.07G161900
Glyma.20G007900
Glyma.01G225200
Glyma.15G248500
Glyma.02G138100
Glyma.07G207200
Glyma.18G057700
Glyma.19G028800
Glyma.18G009200
Glyma.11G101400
Glyma.08G084300
Glyma.05G221100
Glyma.18G195900
Glyma.18G265300
Glyma.18G167300
Glyma.18G195700
Glyma.05G129600
Glyma.08G027600
Glyma.09G277400
Glyma.11G248000
Glyma.18G196000
Glyma.18G211400

Figure.S24

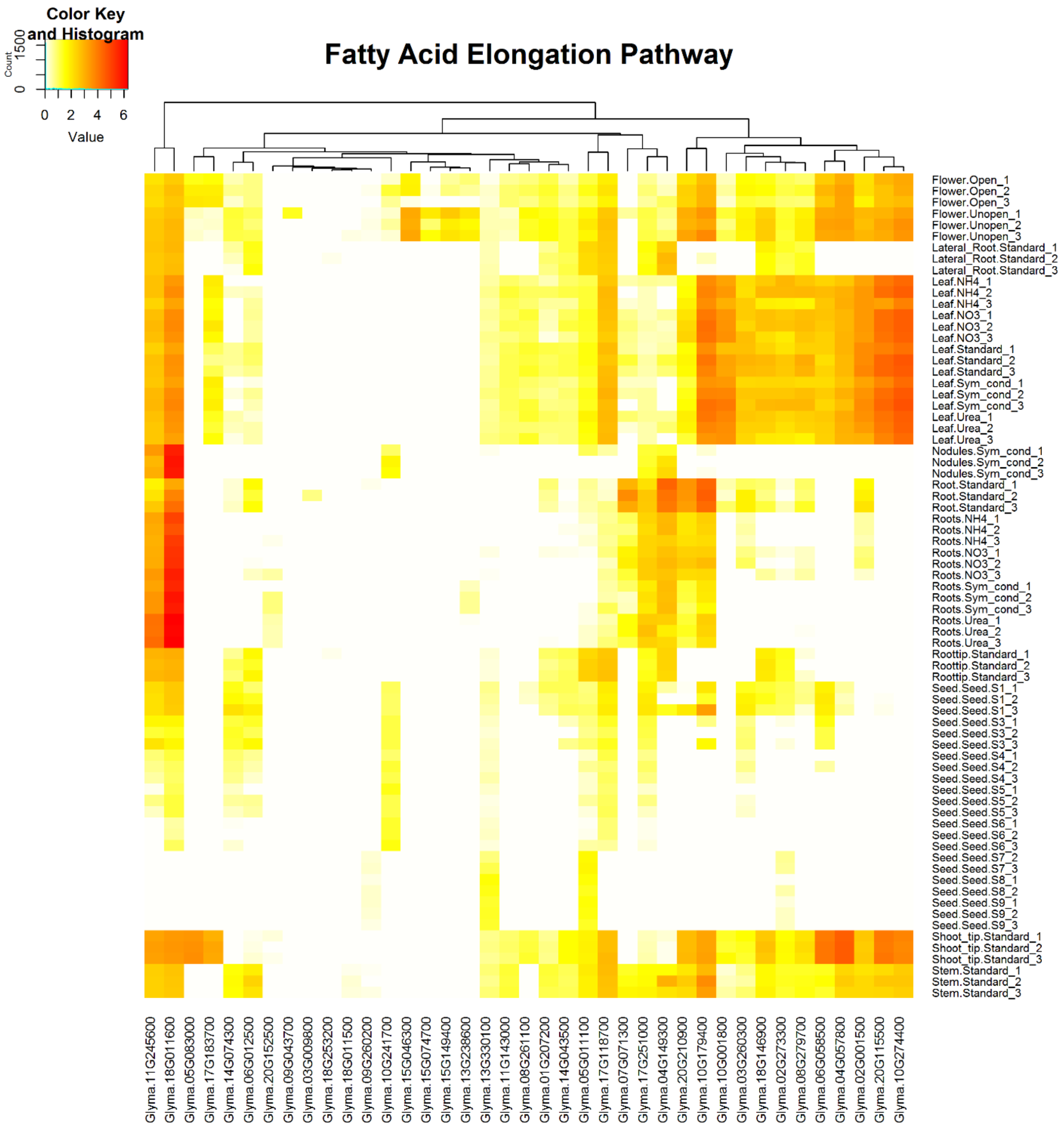
Color Key and Histogram
Count
0
1500
0 2 4 6
Value
Fatty Acid Elongation Pathway
Flower.Open_1
Flower.Open_2
Flower.Open_3
Flower.Unopen_1
Flower.Unopen_2
Flower.Unopen_3
Lateral_Root.Standard_1
Lateral_Root.Standard_2
Lateral_Root.Standard_3
Leaf.NH4_1
Leaf.NH4_2
Leaf.NH4_3
Leaf.NO3_1
Leaf.NO3_2
Leaf.NO3_3
Leaf.Standard_1
Leaf.Standard_2
Leaf.Standard_3
Leaf.Sym_cond_1
Leaf.Sym_cond_2
Leaf.Sym_cond_3
Leaf.Urea_1
Leaf.Urea_2
Leaf.Urea_3
Nodules.Sym_cond_1
Nodules.Sym_cond_2
Nodules.Sym_cond_3
Root.Standard_1
Root.Standard_2
Root.Standard_3
Roots.NH4_1
Roots.NH4_2
Roots.NH4_3
Roots.NO3_1
Roots.NO3_2
Roots.NO3_3
Roots.Sym_cond_1
Roots.Sym_cond_2
Roots.Sym_cond_3
Roots.Urea_1
Roots.Urea_2
Roots.Urea_3
Roottip.Standard_1
Roottip.Standard_2
Roottip.Standard_3
Seed.Seed.S1_1
Seed.Seed.S1_2
Seed.Seed.S1_3
Seed.Seed.S3_1
Seed.Seed.S3_2
Seed.Seed.S3_3
Seed.Seed.S4_1
Seed.Seed.S4_2
Seed.Seed.S4_3
Seed.Seed.S5_1
Seed.Seed.S5_2
Seed.Seed.S5_3
Seed.Seed.S6_1
Seed.Seed.S6_2
Seed.Seed.S6_3
Seed.Seed.S7_2
Seed.Seed.S7_3
Seed.Seed.S8_1
Seed.Seed.S8_2
Seed.Seed.S9_1
Seed.Seed.S9_2
Seed.Seed.S9_3
Shoot_tip.Standard_1
Shoot_tip.Standard_2
Shoot_tip.Standard_3
Stem.Standard_1
Stem.Standard_2
Stem.Standard_3
Glyma.11G245600
Glyma.18G011600
Glyma.05G083000
Glyma.17G183700
Glyma.14G074300
Glyma.06G012500
Glyma.20G152500
Glyma.09G043700
Glyma.03G009800
Glyma.18G253200
Glyma.18G011500
Glyma.09G260200
Glyma.10G241700
Glyma.15G046300
Glyma.15G074700
Glyma.15G149400
Glyma.13G238600
Glyma.13G330100
Glyma.11G143000
Glyma.08G261100
Glyma.01G207200
Glyma.14G043500
Glyma.05G011100
Glyma.17G118700
Glyma.07G071300
Glyma.17G251000
Glyma.04G149300
Glyma.20G210900
Glyma.10G179400
Glyma.10G001800
Glyma.03G260300
Glyma.18G146900
Glyma.02G273300
Glyma.08G279700
Glyma.06G058500
Glyma.04G057800
Glyma.02G001500
Glyma.20G115500
Glyma.10G274400

Figure.S25

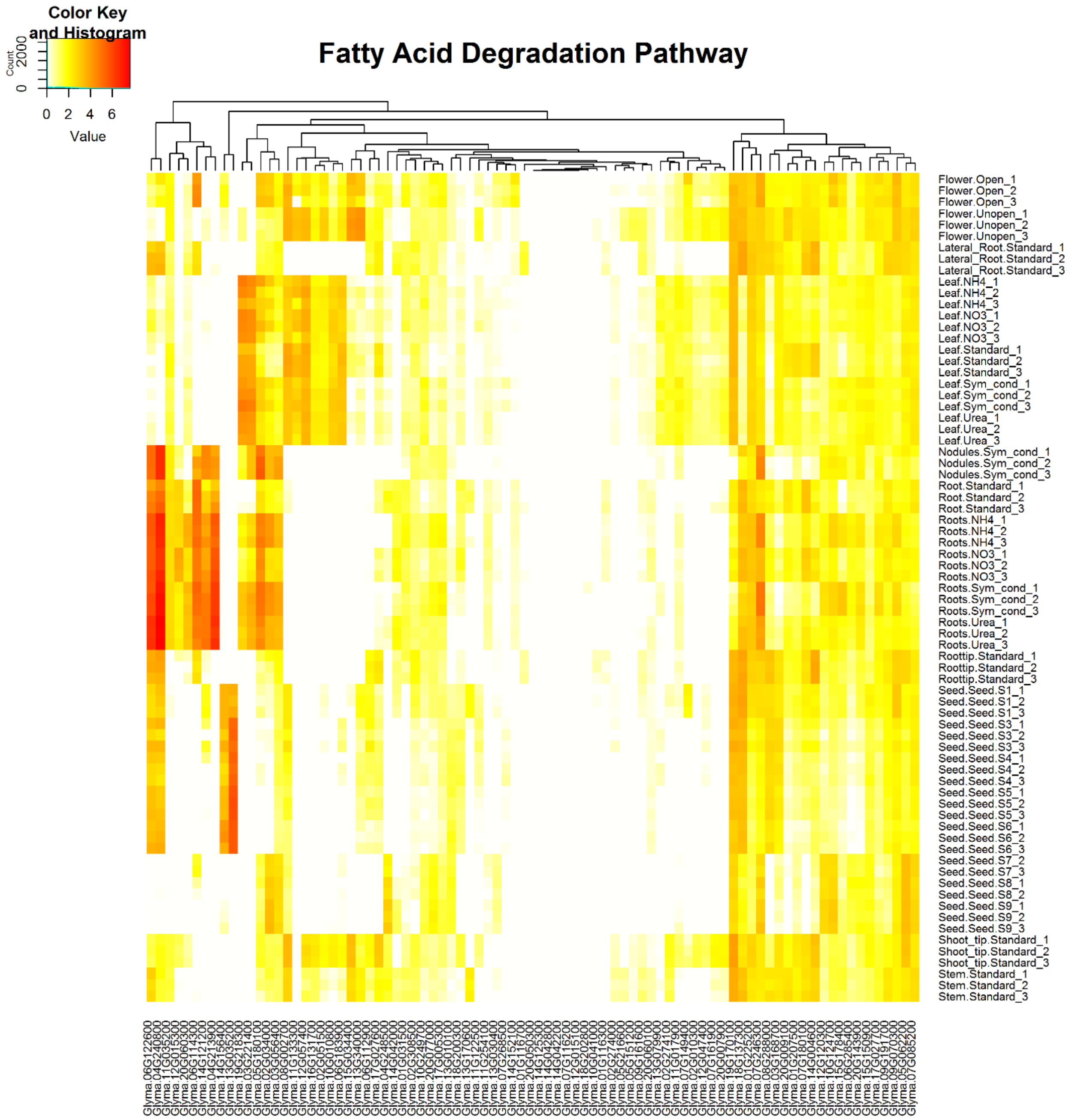
Color Key
and Histogram
Count
0
2000
0 2 4 6
Value
Fatty Acid Degradation Pathway
Flower.Open_1
Flower.Open_2
Flower.Open_3
Flower.Unopen_1
Flower.Unopen_2
Flower.Unopen_3
Lateral_Root.Standard_1
Lateral_Root.Standard_2
Lateral_Root.Standard_3
Leaf.NH4_1
Leaf.NH4_2
Leaf.NH4_3
Leaf.NO3_1
Leaf.NO3_2
Leaf.NO3_3
Leaf.Standard_1
Leaf.Standard_2
Leaf.Standard_3
Leaf.Sym_cond_1
Leaf.Sym_cond_2
Leaf.Sym_cond_3
Leaf.Urea_1
Leaf.Urea_2
Leaf.Urea_3
Nodules.Sym_cond_1
Nodules.Sym_cond_2
Nodules.Sym_cond_3
Root.Standard_1
Root.Standard_2
Root.Standard_3
Roots.NH4_1
Roots.NH4_2
Roots.NH4_3
Roots.NO3_1
Roots.NO3_2
Roots.NO3_3
Roots.Sym_cond_1
Roots.Sym_cond_2
Roots.Sym_cond_3
Roots.Urea_1
Roots.Urea_2
Roots.Urea_3
Roottip.Standard_1
Roottip.Standard_2
Roottip.Standard_3
Seed.Seed.S1_1
Seed.Seed.S1_2
Seed.Seed.S1_3
Seed.Seed.S3_1
Seed.Seed.S3_2
Seed.Seed.S3_3
Seed.Seed.S4_1
Seed.Seed.S4_2
Seed.Seed.S4_3
Seed.Seed.S5_1
Seed.Seed.S5_2
Seed.Seed.S5_3
Seed.Seed.S6_1
Seed.Seed.S6_2
Seed.Seed.S6_3
Seed.Seed.S7_2
Seed.Seed.S7_3
Seed.Seed.S8_1
Seed.Seed.S8_2
Seed.Seed.S9_1
Seed.Seed.S9_2
Seed.Seed.S9_3
Shoot_tip.Standard_1
Shoot_tip.Standard_2
Shoot_tip.Standard_3
Stem.Standard_1
Stem.Standard_2
Stem.Standard_3

Figure.S26

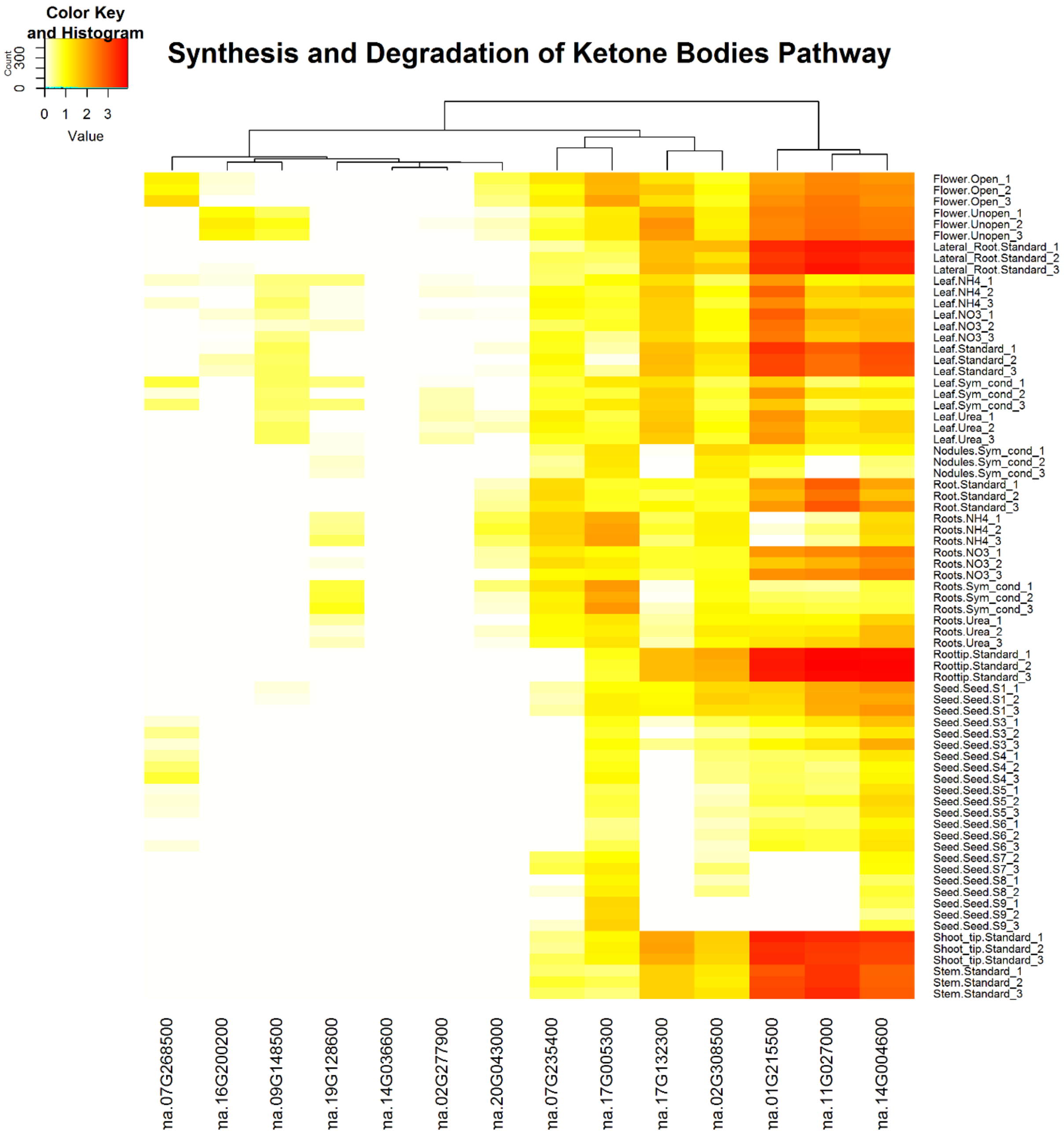
Color Key
and Histogram
Count
0
300
0 1 2 3
Value
Synthesis and Degradation of Ketone Bodies Pathway
Flower.Open_1
Flower.Open_2
Flower.Open_3
Flower.Unopen_1
Flower.Unopen_2
Flower.Unopen_3
Lateral_Root.Standard_1
Lateral_Root.Standard_2
Lateral_Root.Standard_3
Leaf.NH4_1
Leaf.NH4_2
Leaf.NH4_3
Leaf.NO3_1
Leaf.NO3_2
Leaf.NO3_3
Leaf.Standard_1
Leaf.Standard_2
Leaf.Standard_3
Leaf.Sym_cond_1
Leaf.Sym_cond_2
Leaf.Sym_cond_3
Leaf.Urea_1
Leaf.Urea_2
Leaf.Urea_3
Nodules.Sym_cond_1
Nodules.Sym_cond_2
Nodules.Sym_cond_3
Root.Standard_1
Root.Standard_2
Root.Standard_3
Roots.NH4_1
Roots.NH4_2
Roots.NH4_3
Roots.NO3_1
Roots.NO3_2
Roots.NO3_3
Roots.Sym_cond_1
Roots.Sym_cond_2
Roots.Sym_cond_3
Roots.Urea_1
Roots.Urea_2
Roots.Urea_3
Roottip.Standard_1
Roottip.Standard_2
Roottip.Standard_3
Seed.Seed.S1_1
Seed.Seed.S1_2
Seed.Seed.S1_3
Seed.Seed.S3_1
Seed.Seed.S3_2
Seed.Seed.S3_3
Seed.Seed.S4_1
Seed.Seed.S4_2
Seed.Seed.S4_3
Seed.Seed.S5_1
Seed.Seed.S5_2
Seed.Seed.S5_3
Seed.Seed.S6_1
Seed.Seed.S6_2
Seed.Seed.S6_3
Seed.Seed.S7_2
Seed.Seed.S7_3
Seed.Seed.S8_1
Seed.Seed.S8_2
Seed.Seed.S9_1
Seed.Seed.S9_2
Seed.Seed.S9_3
Shoot_tip.Standard_1
Shoot_tip.Standard_2
Shoot_tip.Standard_3
Stem.Standard_1
Stem.Standard_2
Stem.Standard_3
na.07G268500
na.16G200200
na.09G148500
na.19G128600
na.14G036600
na.02G277900
na.20G043000
na.07G235400
na.17G005300
na.17G132300
na.02G308500
na.01G215500
na.11G027000
na.14G004600

Figure.S27

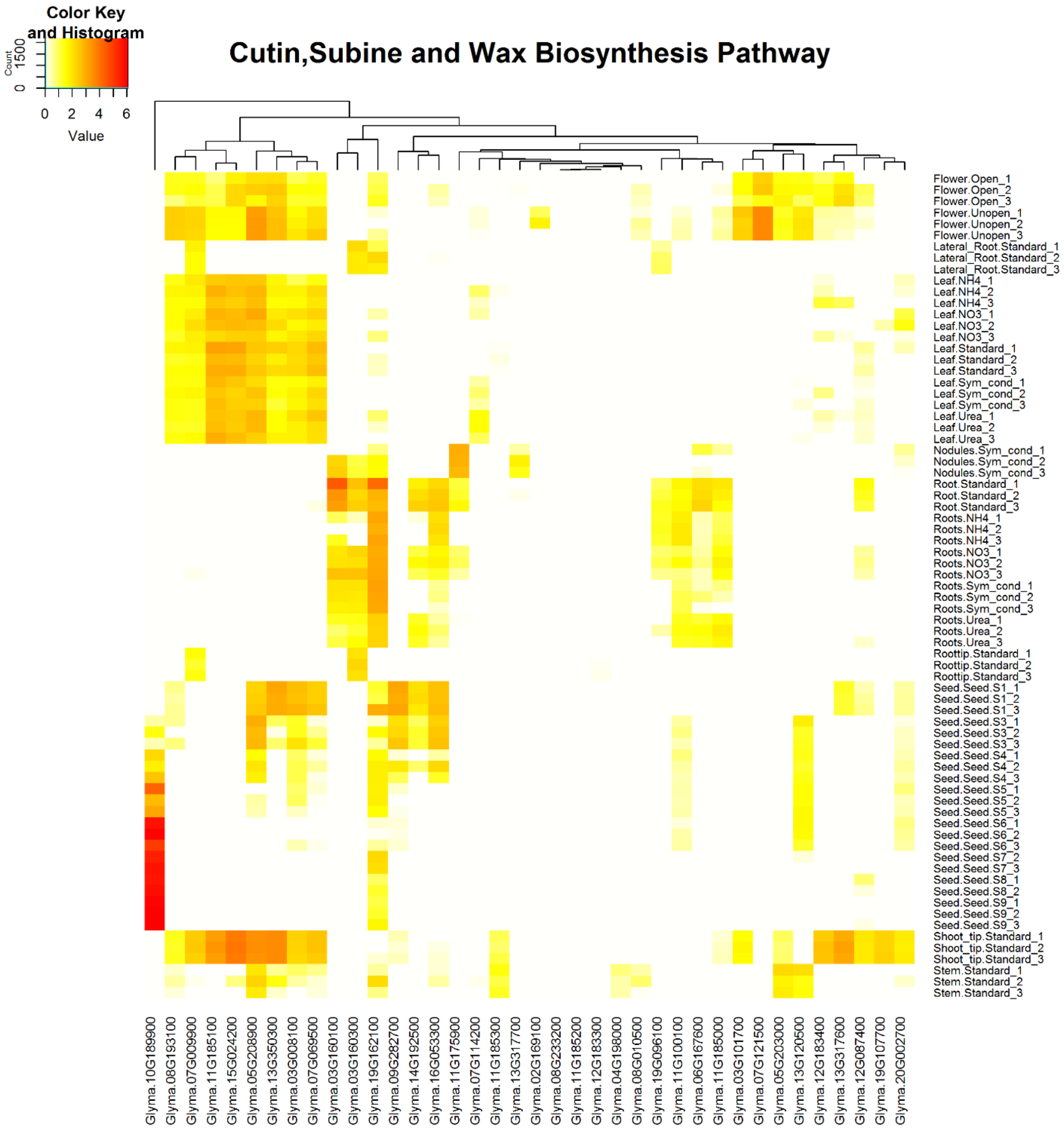
Color Key
and Histogram
Count
0
1500
0 2 4 6
Value
Cutin,Subine and Wax Biosynthesis Pathway
Flower.Open_1
Flower.Open_2
Flower.Open_3
Flower.Unopen_1
Flower.Unopen_2
Flower.Unopen_3
Lateral_Root.Standard_1
Lateral_Root.Standard_2
Lateral_Root.Standard_3
Leaf.NH4_1
Leaf.NH4_2
Leaf.NH4_3
Leaf.NO3_1
Leaf.NO3_2
Leaf.NO3_3
Leaf.Standard_1
Leaf.Standard_2
Leaf.Standard_3
Leaf.Sym_cond_1
Leaf.Sym_cond_2
Leaf.Sym_cond_3
Leaf.Urea_1
Leaf.Urea_2
Leaf.Urea_3
Nodules.Sym_cond_1
Nodules.Sym_cond_2
Nodules.Sym_cond_3
Root.Standard_1
Root.Standard_2
Root.Standard_3
Roots.NH4_1
Roots.NH4_2
Roots.NH4_3
Roots.NO3_1
Roots.NO3_2
Roots.NO3_3
Roots.Sym_cond_1
Roots.Sym_cond_2
Roots.Sym_cond_3
Roots.Urea_1
Roots.Urea_2
Roots.Urea_3
Roottip.Standard_1
Roottip.Standard_2
Roottip.Standard_3
Seed.Seed.S1_1
Seed.Seed.S1_2
Seed.Seed.S1_3
Seed.Seed.S3_1
Seed.Seed.S3_2
Seed.Seed.S3_3
Seed.Seed.S4_1
Seed.Seed.S4_2
Seed.Seed.S4_3
Seed.Seed.S5_1
Seed.Seed.S5_2
Seed.Seed.S5_3
Seed.Seed.S6_1
Seed.Seed.S6_2
Seed.Seed.S6_3
Seed.Seed.S7_2
Seed.Seed.S7_3
Seed.Seed.S8_1
Seed.Seed.S8_2
Seed.Seed.S9_1
Seed.Seed.S9_2
Seed.Seed.S9_3
Shoot_tip.Standard_1
Shoot_tip.Standard_2
Shoot_tip.Standard_3
Stem.Standard_1
Stem.Standard_2
Stem.Standard_3
Glyma.10G189900
Glyma.08G193100
Glyma.07G009900
Glyma.11G185100
Glyma.15G024200
Glyma.05G208900
Glyma.13G350300
Glyma.03G008100
Glyma.07G069500
Glyma.03G160100
Glyma.03G160300
Glyma.19G162100
Glyma.09G282700
Glyma.14G192500
Glyma.16G053300
Glyma.11G175900
Glyma.07G114200
Glyma.11G185300
Glyma.13G317700
Glyma.02G169100
Glyma.08G233200
Glyma.11G185200
Glyma.12G183300
Glyma.04G198000
Glyma.08G010500
Glyma.19G096100
Glyma.11G100100
Glyma.06G167600
Glyma.11G185000
Glyma.03G101700
Glyma.07G121500
Glyma.05G203000
Glyma.13G120500
Glyma.12G183400
Glyma.13G317600
Glyma.12G087400
Glyma.19G107700
Glyma.20G002700

Figure.S28

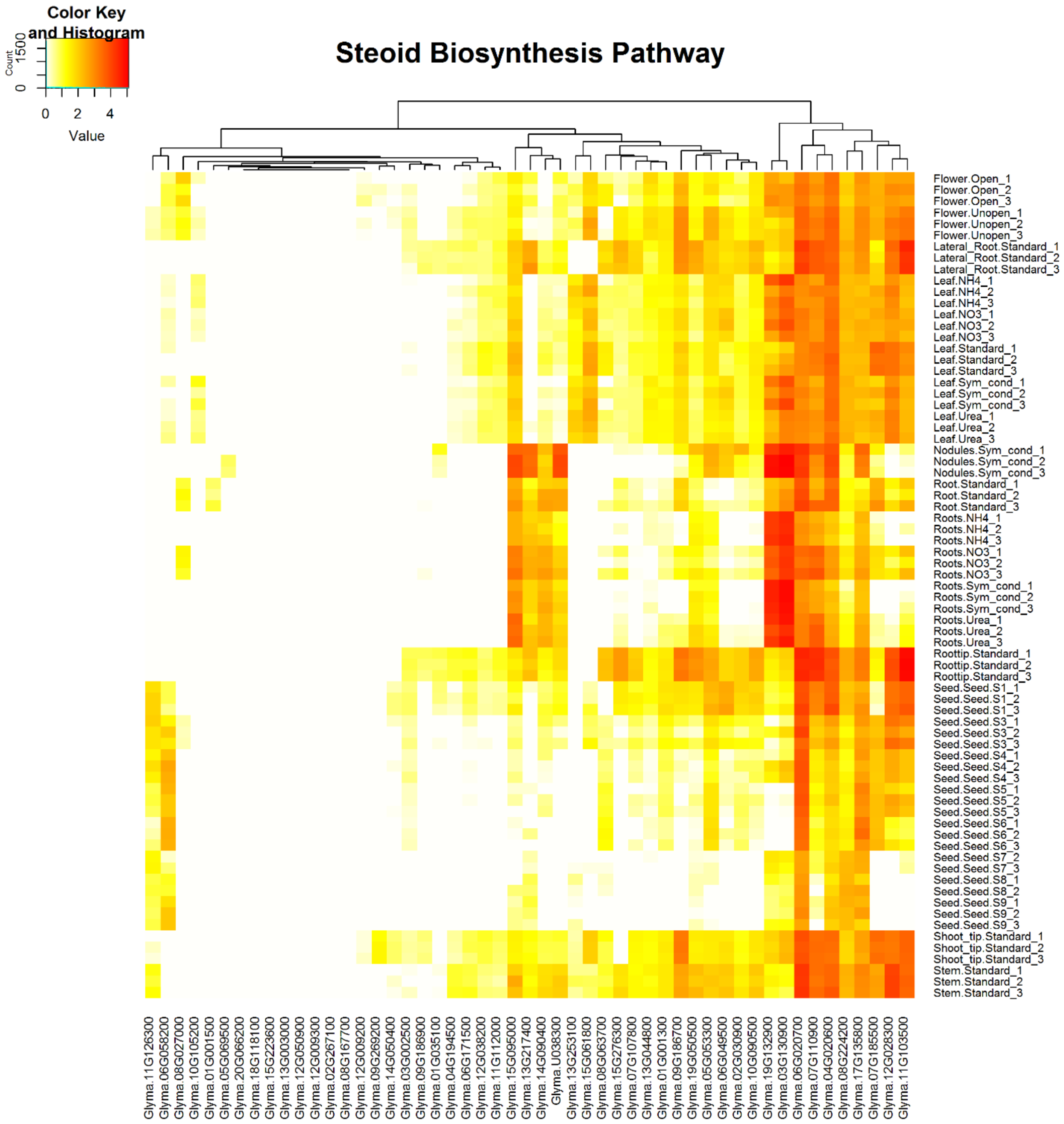
Color Key and Histogram
Count
0
1500
0
2
4
Value
Steoid Biosynthesis Pathway
Flower.Open_1
Flower.Open_2
Flower.Open_3
Flower.Unopen_1
Flower.Unopen_2
Flower.Unopen_3
Lateral_Root.Standard_1
Lateral_Root.Standard_2
Lateral_Root.Standard_3
Leaf.NH4_1
Leaf.NH4_2
Leaf.NH4_3
Leaf.NO3_1
Leaf.NO3_2
Leaf.NO3_3
Leaf.Standard_1
Leaf.Standard_2
Leaf.Standard_3
Leaf.Sym_cond_1
Leaf.Sym_cond_2
Leaf.Sym_cond_3
Leaf.Urea_1
Leaf.Urea_2
Leaf.Urea_3
Nodules.Sym_cond_1
Nodules.Sym_cond_2
Nodules.Sym_cond_3
Root.Standard_1
Root.Standard_2
Root.Standard_3
Roots.NH4_1
Roots.NH4_2
Roots.NH4_3
Roots.NO3_1
Roots.NO3_2
Roots.NO3_3
Roots.Sym_cond_1
Roots.Sym_cond_2
Roots.Sym_cond_3
Roots.Urea_1
Roots.Urea_2
Roots.Urea_3
Roottip.Standard_1
Roottip.Standard_2
Roottip.Standard_3
Seed.Seed.S1_1
Seed.Seed.S1_2
Seed.Seed.S1_3
Seed.Seed.S3_1
Seed.Seed.S3_2
Seed.Seed.S3_3
Seed.Seed.S4_1
Seed.Seed.S4_2
Seed.Seed.S4_3
Seed.Seed.S5_1
Seed.Seed.S5_2
Seed.Seed.S5_3
Seed.Seed.S6_1
Seed.Seed.S6_2
Seed.Seed.S6_3
Seed.Seed.S7_2
Seed.Seed.S7_3
Seed.Seed.S8_1
Seed.Seed.S8_2
Seed.Seed.S9_1
Seed.Seed.S9_2
Seed.Seed.S9_3
Shoot_tip.Standard_1
Shoot_tip.Standard_2
Shoot_tip.Standard_3
Stem.Standard_1
Stem.Standard_2
Stem.Standard_3
Glyma.11G126300
Glyma.06G058200
Glyma.08G027000
Glyma.10G105200
Glyma.01G001500
Glyma.05G069500
Glyma.20G066200
Glyma.18G118100
Glyma.15G223600
Glyma.13G003000
Glyma.12G050900
Glyma.12G009300
Glyma.02G267100
Glyma.08G167700
Glyma.12G009200
Glyma.09G269200
Glyma.14G050400
Glyma.03G002500
Glyma.09G186900
Glyma.01G035100
Glyma.04G194500
Glyma.06G171500
Glyma.12G038200
Glyma.11G112000
Glyma.15G095000
Glyma.13G217400
Glyma.14G090400
Glyma.U038300
Glyma.13G253100
Glyma.15G061800
Glyma.08G063700
Glyma.15G276300
Glyma.07G107800
Glyma.13G044800
Glyma.01G001300
Glyma.09G186700
Glyma.19G050500
Glyma.05G053300
Glyma.06G049500
Glyma.02G030900
Glyma.10G090500
Glyma.19G132900
Glyma.03G130900
Glyma.06G020700
Glyma.07G110900
Glyma.04G020600
Glyma.08G224200
Glyma.17G135800
Glyma.07G185500
Glyma.12G028300
Glyma.11G103500

Figure.S29

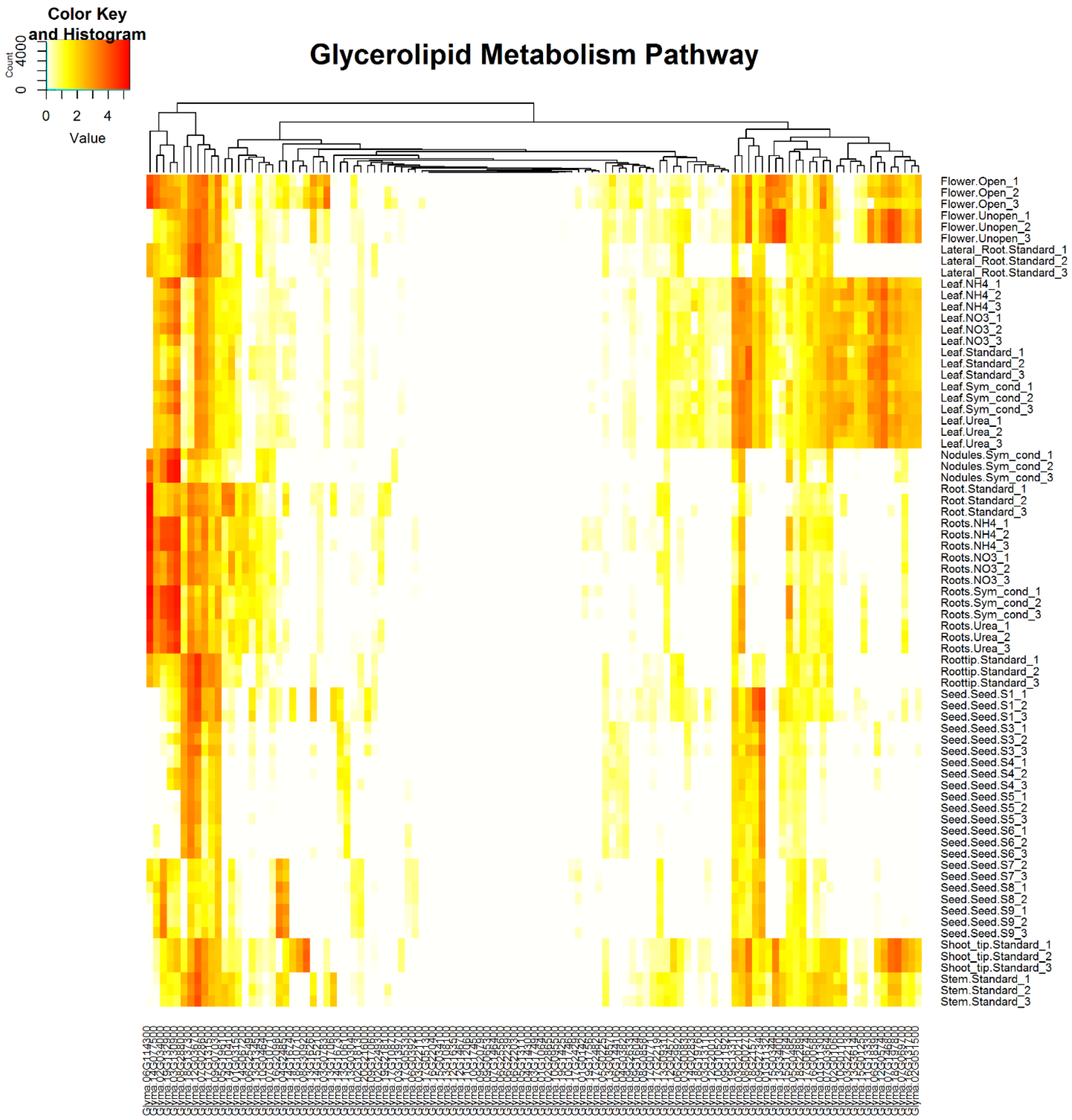
Color Key
and Histogram
Count
0
4000
0
2
4
Value
Glycerolipid Metabolism Pathway
Flower.Open_1
Flower.Open_2
Flower.Open_3
Flower.Unopen_1
Flower.Unopen_2
Flower.Unopen_3
Lateral_Root.Standard_1
Lateral_Root.Standard_2
Lateral_Root.Standard_3
Leaf.NH4_1
Leaf.NH4_2
Leaf.NH4_3
Leaf.NO3_1
Leaf.NO3_2
Leaf.NO3_3
Leaf.Standard_1
Leaf.Standard_2
Leaf.Standard_3
Leaf.Sym_cond_1
Leaf.Sym_cond_2
Leaf.Sym_cond_3
Leaf.Urea_1
Leaf.Urea_2
Leaf.Urea_3
Nodules.Sym_cond_1
Nodules.Sym_cond_2
Nodules.Sym_cond_3
Root.Standard_1
Root.Standard_2
Root.Standard_3
Roots.NH4_1
Roots.NH4_2
Roots.NH4_3
Roots.NO3_1
Roots.NO3_2
Roots.NO3_3
Roots.Sym_cond_1
Roots.Sym_cond_2
Roots.Sym_cond_3
Roots.Urea_1
Roots.Urea_2
Roots.Urea_3
Roottip.Standard_1
Roottip.Standard_2
Roottip.Standard_3
Seed.Seed.S1_1
Seed.Seed.S1_2
Seed.Seed.S1_3
Seed.Seed.S3_1
Seed.Seed.S3_2
Seed.Seed.S3_3
Seed.Seed.S4_1
Seed.Seed.S4_2
Seed.Seed.S4_3
Seed.Seed.S5_1
Seed.Seed.S5_2
Seed.Seed.S5_3
Seed.Seed.S6_1
Seed.Seed.S6_2
Seed.Seed.S6_3
Seed.Seed.S7_2
Seed.Seed.S7_3
Seed.Seed.S8_1
Seed.Seed.S8_2
Seed.Seed.S9_1
Seed.Seed.S9_2
Seed.Seed.S9_3
Shoot_tip.Standard_1
Shoot_tip.Standard_2
Shoot_tip.Standard_3
Stem.Standard_1
Stem.Standard_2
Stem.Standard_3

Figure.S30

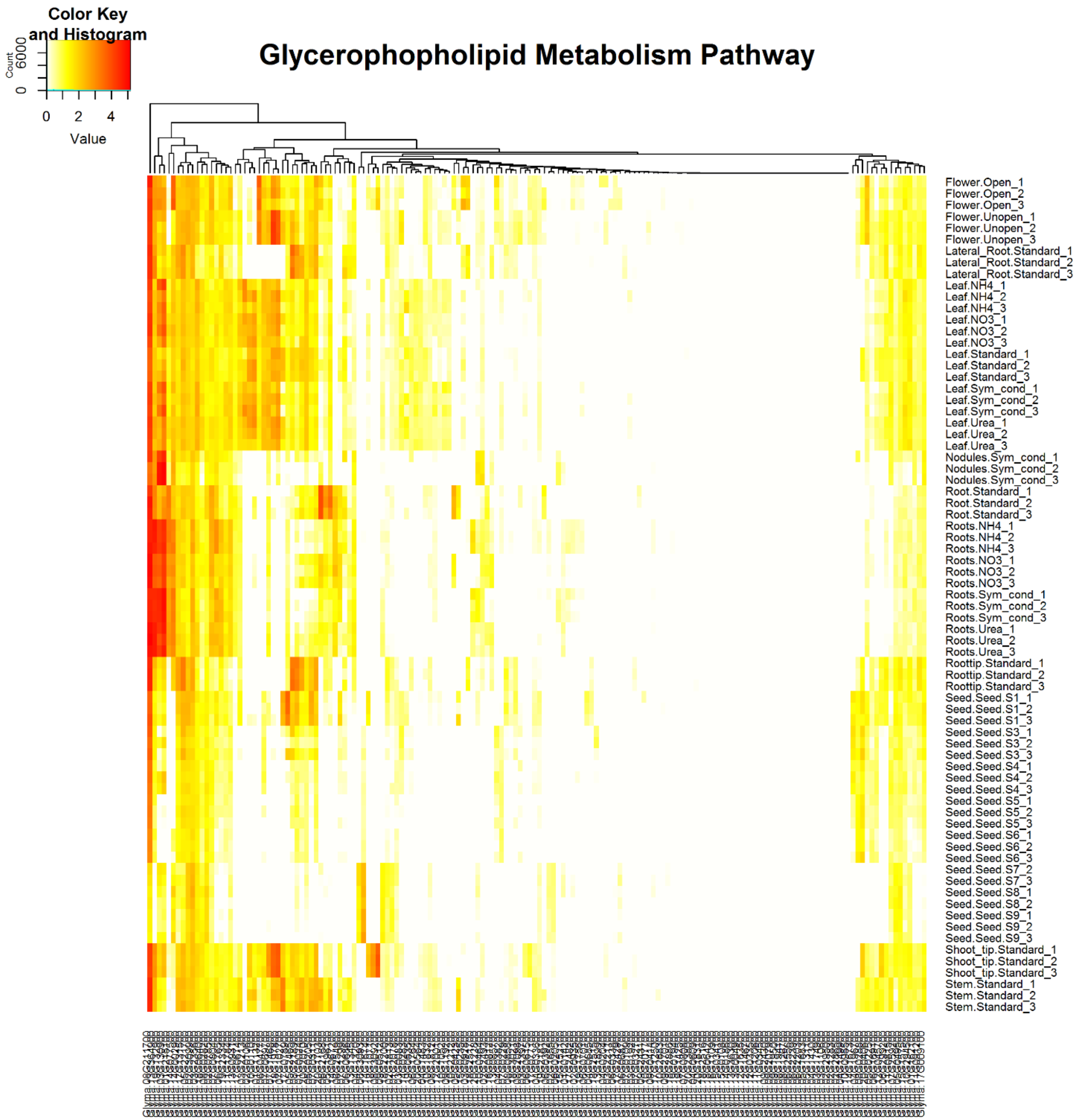
Color Key
and Histogram
Count
0
6000
0
2
4
Value
Glycerophospholipid Metabolism Pathway
Flower.Open_1
Flower.Open_2
Flower.Open_3
Flower.Unopen_1
Flower.Unopen_2
Flower.Unopen_3
Lateral_Root.Standard_1
Lateral_Root.Standard_2
Lateral_Root.Standard_3
Leaf.NH4_1
Leaf.NH4_2
Leaf.NH4_3
Leaf.NO3_1
Leaf.NO3_2
Leaf.NO3_3
Leaf.Standard_1
Leaf.Standard_2
Leaf.Standard_3
Leaf.Sym_cond_1
Leaf.Sym_cond_2
Leaf.Sym_cond_3
Leaf.Urea_1
Leaf.Urea_2
Leaf.Urea_3
Nodules.Sym_cond_1
Nodules.Sym_cond_2
Nodules.Sym_cond_3
Root.Standard_1
Root.Standard_2
Root.Standard_3
Roots.NH4_1
Roots.NH4_2
Roots.NH4_3
Roots.NO3_1
Roots.NO3_2
Roots.NO3_3
Roots.Sym_cond_1
Roots.Sym_cond_2
Roots.Sym_cond_3
Roots.Urea_1
Roots.Urea_2
Roots.Urea_3
Roottip.Standard_1
Roottip.Standard_2
Roottip.Standard_3
Seed.Seed.S1_1
Seed.Seed.S1_2
Seed.Seed.S1_3
Seed.Seed.S3_1
Seed.Seed.S3_2
Seed.Seed.S3_3
Seed.Seed.S4_1
Seed.Seed.S4_2
Seed.Seed.S4_3
Seed.Seed.S5_1
Seed.Seed.S5_2
Seed.Seed.S5_3
Seed.Seed.S6_1
Seed.Seed.S6_2
Seed.Seed.S6_3
Seed.Seed.S7_2
Seed.Seed.S7_3
Seed.Seed.S8_1
Seed.Seed.S8_2
Seed.Seed.S9_1
Seed.Seed.S9_2
Seed.Seed.S9_3
Shoot_tip.Standard_1
Shoot_tip.Standard_2
Shoot_tip.Standard_3
Stem.Standard_1
Stem.Standard_2
Stem.Standard_3

Figure.S31

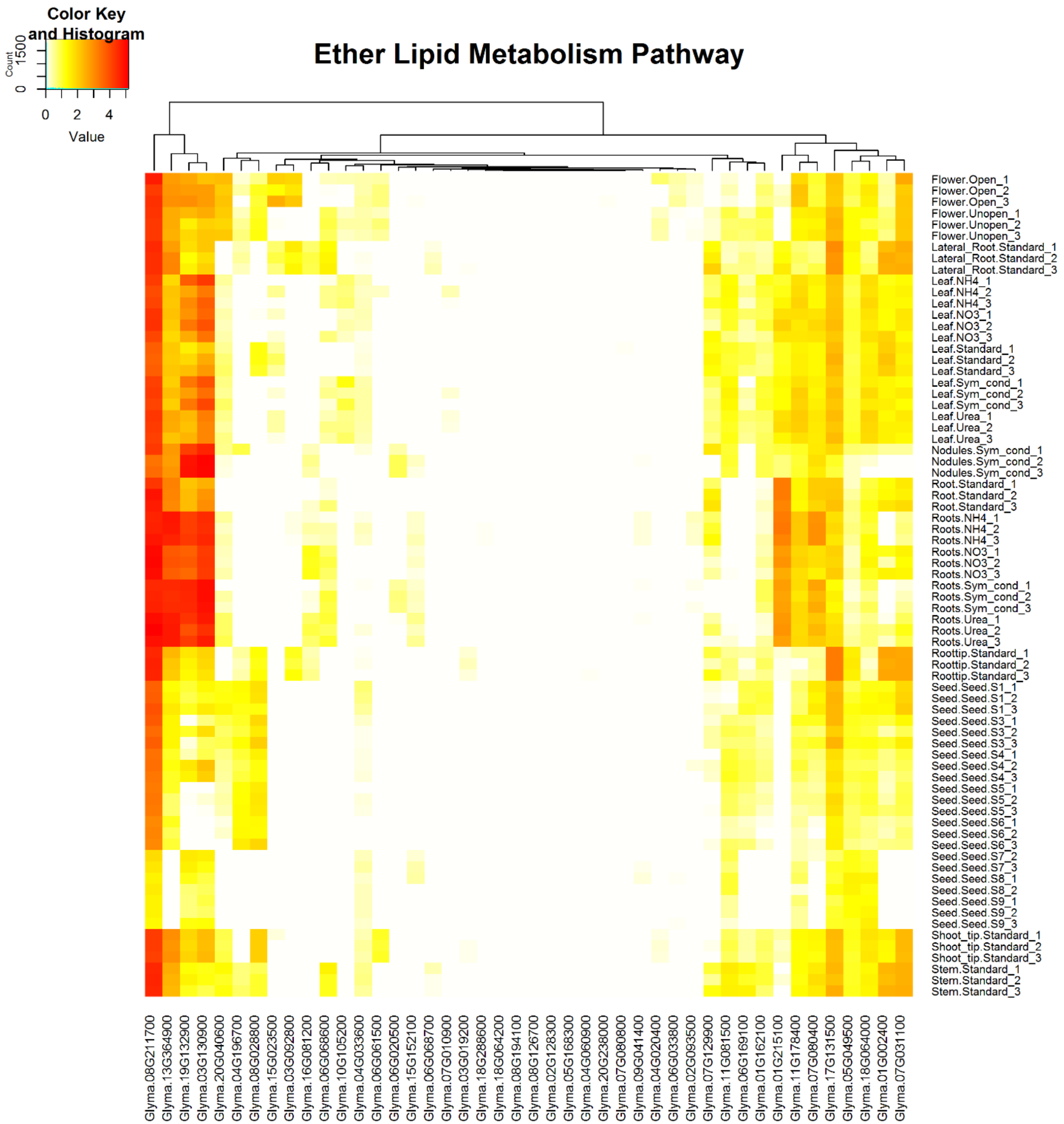

Color Key
and Histogram
Count
0
1500
0
2
4
Value
Ether Lipid Metabolism Pathway
Flower.Open_1
Flower.Open_2
Flower.Open_3
Flower.Unopen_1
Flower.Unopen_2
Flower.Unopen_3
Lateral_Root.Standard_1
Lateral_Root.Standard_2
Lateral_Root.Standard_3
Leaf.NH4_1
Leaf.NH4_2
Leaf.NH4_3
Leaf.NO3_1
Leaf.NO3_2
Leaf.NO3_3
Leaf.Standard_1
Leaf.Standard_2
Leaf.Standard_3
Leaf.Sym_cond_1
Leaf.Sym_cond_2
Leaf.Sym_cond_3
Leaf.Urea_1
Leaf.Urea_2
Leaf.Urea_3
Nodules.Sym_cond_1
Nodules.Sym_cond_2
Nodules.Sym_cond_3
Root.Standard_1
Root.Standard_2
Root.Standard_3
Roots.NH4_1
Roots.NH4_2
Roots.NH4_3
Roots.NO3_1
Roots.NO3_2
Roots.NO3_3
Roots.Sym_cond_1
Roots.Sym_cond_2
Roots.Sym_cond_3
Roots.Urea_1
Roots.Urea_2
Roots.Urea_3
Roottip.Standard_1
Roottip.Standard_2
Roottip.Standard_3
Seed.Seed.S1_1
Seed.Seed.S1_2
Seed.Seed.S1_3
Seed.Seed.S3_1
Seed.Seed.S3_2
Seed.Seed.S3_3
Seed.Seed.S4_1
Seed.Seed.S4_2
Seed.Seed.S4_3
Seed.Seed.S5_1
Seed.Seed.S5_2
Seed.Seed.S5_3
Seed.Seed.S6_1
Seed.Seed.S6_2
Seed.Seed.S6_3
Seed.Seed.S7_2
Seed.Seed.S7_3
Seed.Seed.S8_1
Seed.Seed.S8_2
Seed.Seed.S9_1
Seed.Seed.S9_2
Seed.Seed.S9_3
Shoot_tip.Standard_1
Shoot_tip.Standard_2
Shoot_tip.Standard_3
Stem.Standard_1
Stem.Standard_2
Stem.Standard_3
Glyma.08G211700
Glyma.13G364900
Glyma.19G132900
Glyma.03G130900
Glyma.20G040600
Glyma.04G196700
Glyma.08G028800
Glyma.15G023500
Glyma.03G092800
Glyma.16G081200
Glyma.06G068600
Glyma.10G105200
Glyma.04G033600
Glyma.06G061500
Glyma.06G020500
Glyma.15G152100
Glyma.06G068700
Glyma.07G010900
Glyma.03G019200
Glyma.18G288600
Glyma.18G064200
Glyma.08G194100
Glyma.08G126700
Glyma.02G128300
Glyma.05G168300
Glyma.04G060900
Glyma.20G238000
Glyma.07G080800
Glyma.09G041400
Glyma.04G020400
Glyma.06G033800
Glyma.02G093500
Glyma.07G129900
Glyma.11G081500
Glyma.06G169100
Glyma.01G162100
Glyma.01G215100
Glyma.11G178400
Glyma.07G080400
Glyma.17G131500
Glyma.05G049500
Glyma.18G064000
Glyma.01G002400
Glyma.07G031100

Figure.S32

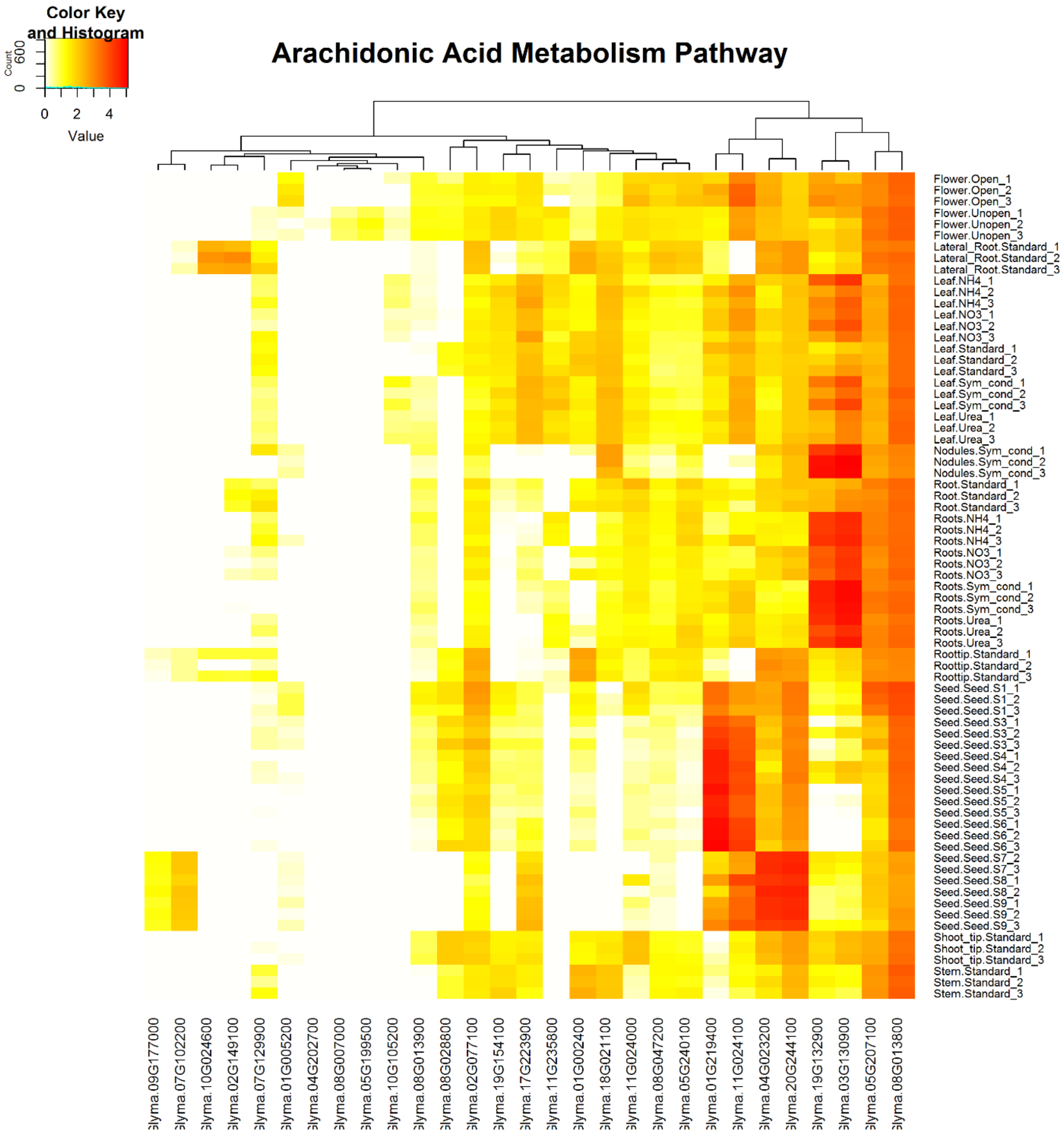
Color Key
and Histogram
Count
0
600
0
2
4
Value
Arachidonic Acid Metabolism Pathway
Flower.Open_1
Flower.Open_2
Flower.Open_3
Flower.Unopen_1
Flower.Unopen_2
Flower.Unopen_3
Lateral_Root.Standard_1
Lateral_Root.Standard_2
Lateral_Root.Standard_3
Leaf.NH4_1
Leaf.NH4_2
Leaf.NH4_3
Leaf.NO3_1
Leaf.NO3_2
Leaf.NO3_3
Leaf.Standard_1
Leaf.Standard_2
Leaf.Standard_3
Leaf.Sym_cond_1
Leaf.Sym_cond_2
Leaf.Sym_cond_3
Leaf.Urea_1
Leaf.Urea_2
Leaf.Urea_3
Nodules.Sym_cond_1
Nodules.Sym_cond_2
Nodules.Sym_cond_3
Root.Standard_1
Root.Standard_2
Root.Standard_3
Roots.NH4_1
Roots.NH4_2
Roots.NH4_3
Roots.NO3_1
Roots.NO3_2
Roots.NO3_3
Roots.Sym_cond_1
Roots.Sym_cond_2
Roots.Sym_cond_3
Roots.Urea_1
Roots.Urea_2
Roots.Urea_3
Roottip.Standard_1
Roottip.Standard_2
Roottip.Standard_3
Seed.Seed.S1_1
Seed.Seed.S1_2
Seed.Seed.S1_3
Seed.Seed.S3_1
Seed.Seed.S3_2
Seed.Seed.S3_3
Seed.Seed.S4_1
Seed.Seed.S4_2
Seed.Seed.S4_3
Seed.Seed.S5_1
Seed.Seed.S5_2
Seed.Seed.S5_3
Seed.Seed.S6_1
Seed.Seed.S6_2
Seed.Seed.S6_3
Seed.Seed.S7_2
Seed.Seed.S7_3
Seed.Seed.S8_1
Seed.Seed.S8_2
Seed.Seed.S9_1
Seed.Seed.S9_2
Seed.Seed.S9_3
Shoot_tip.Standard_1
Shoot_tip.Standard_2
Shoot_tip.Standard_3
Stem.Standard_1
Stem.Standard_2
Stem.Standard_3
lyma.09G177000
lyma.07G102200
lyma.10G024600
lyma.02G149100
lyma.07G129900
lyma.01G005200
lyma.04G202700
lyma.08G007000
lyma.05G199500
lyma.10G105200
lyma.08G013900
lyma.08G028800
lyma.02G077100
lyma.19G154100
lyma.17G223900
lyma.11G235800
lyma.01G002400
lyma.18G021100
lyma.11G024000
lyma.08G047200
lyma.05G240100
lyma.01G219400
lyma.11G024100
lyma.04G023200
lyma.20G244100
lyma.19G132900
lyma.03G130900
lyma.05G207100
lyma.08G013800

Figure.S33

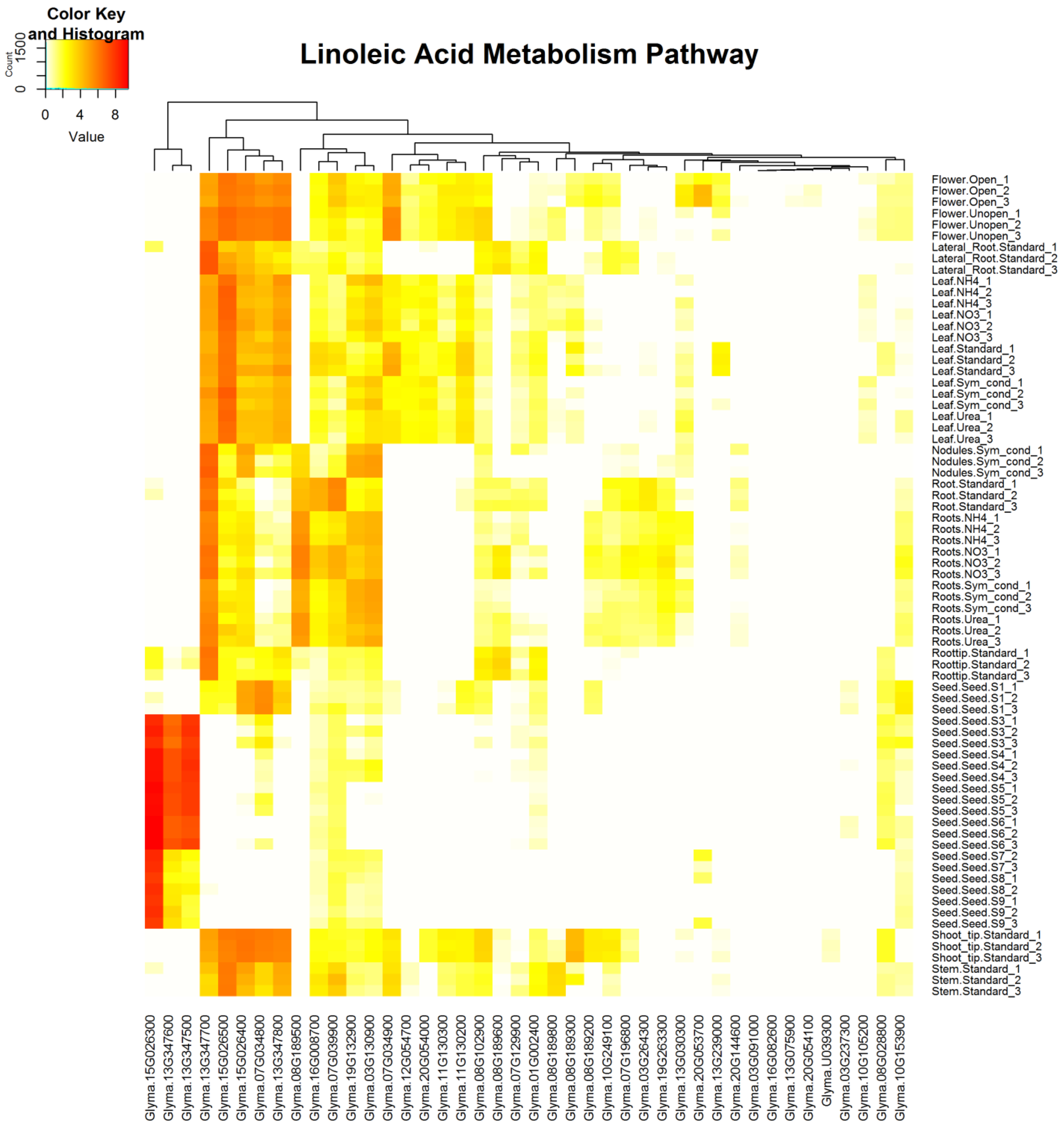

Color Key
and Histogram
Count
0
1500
0
4
8
Value
Linoleic Acid Metabolism Pathway
Flower.Open_1
Flower.Open_2
Flower.Open_3
Flower.Unopen_1
Flower.Unopen_2
Flower.Unopen_3
Lateral_Root.Standard_1
Lateral_Root.Standard_2
Lateral_Root.Standard_3
Leaf.NH4_1
Leaf.NH4_2
Leaf.NH4_3
Leaf.NO3_1
Leaf.NO3_2
Leaf.NO3_3
Leaf.Standard_1
Leaf.Standard_2
Leaf.Standard_3
Leaf.Sym_cond_1
Leaf.Sym_cond_2
Leaf.Sym_cond_3
Leaf.Urea_1
Leaf.Urea_2
Leaf.Urea_3
Nodules.Sym_cond_1
Nodules.Sym_cond_2
Nodules.Sym_cond_3
Root.Standard_1
Root.Standard_2
Root.Standard_3
Roots.NH4_1
Roots.NH4_2
Roots.NH4_3
Roots.NO3_1
Roots.NO3_2
Roots.NO3_3
Roots.Sym_cond_1
Roots.Sym_cond_2
Roots.Sym_cond_3
Roots.Urea_1
Roots.Urea_2
Roots.Urea_3
Roottip.Standard_1
Roottip.Standard_2
Roottip.Standard_3
Seed.Seed.S1_1
Seed.Seed.S1_2
Seed.Seed.S1_3
Seed.Seed.S3_1
Seed.Seed.S3_2
Seed.Seed.S3_3
Seed.Seed.S4_1
Seed.Seed.S4_2
Seed.Seed.S4_3
Seed.Seed.S5_1
Seed.Seed.S5_2
Seed.Seed.S5_3
Seed.Seed.S6_1
Seed.Seed.S6_2
Seed.Seed.S6_3
Seed.Seed.S7_2
Seed.Seed.S7_3
Seed.Seed.S8_1
Seed.Seed.S8_2
Seed.Seed.S9_1
Seed.Seed.S9_2
Seed.Seed.S9_3
Shoot_tip.Standard_1
Shoot_tip.Standard_2
Shoot_tip.Standard_3
Stem.Standard_1
Stem.Standard_2
Stem.Standard_3
Glyma.15G026300
Glyma.13G347600
Glyma.13G347500
Glyma.13G347700
Glyma.15G026500
Glyma.15G026400
Glyma.07G034800
Glyma.13G347800
Glyma.08G189500
Glyma.16G008700
Glyma.07G039900
Glyma.19G132900
Glyma.03G130900
Glyma.07G034900
Glyma.12G054700
Glyma.20G054000
Glyma.11G130300
Glyma.11G130200
Glyma.08G102900
Glyma.08G189600
Glyma.07G129900
Glyma.01G002400
Glyma.08G189800
Glyma.08G189300
Glyma.08G189200
Glyma.10G249100
Glyma.07G196800
Glyma.03G264300
Glyma.19G263300
Glyma.13G030300
Glyma.20G053700
Glyma.13G239000
Glyma.20G144600
Glyma.03G091000
Glyma.16G082600
Glyma.13G075900
Glyma.20G054100
Glyma.U039300
Glyma.03G237300
Glyma.10G105200
Glyma.08G028800
Glyma.10G153900

Figure.S34

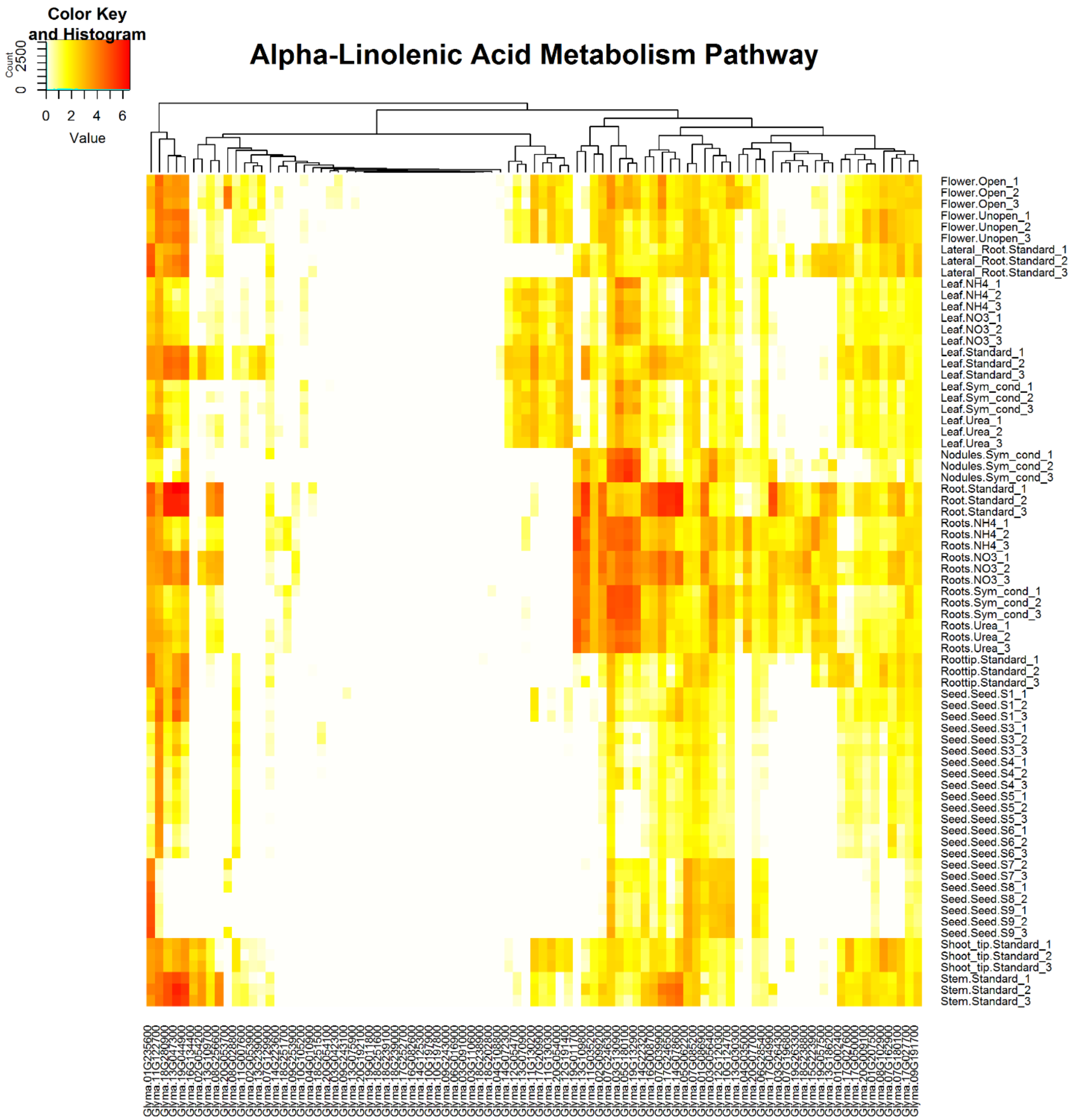
Color Key
and Histogram
Count
0
2500
0 2 4 6
Value
Alpha-Linolenic Acid Metabolism Pathway
Flower.Open_1
Flower.Open_2
Flower.Open_3
Flower.Unopen_1
Flower.Unopen_2
Flower.Unopen_3
Lateral_Root.Standard_1
Lateral_Root.Standard_2
Lateral_Root.Standard_3
Leaf.NH4_1
Leaf.NH4_2
Leaf.NH4_3
Leaf.NO3_1
Leaf.NO3_2
Leaf.NO3_3
Leaf.Standard_1
Leaf.Standard_2
Leaf.Standard_3
Leaf.Sym_cond_1
Leaf.Sym_cond_2
Leaf.Sym_cond_3
Leaf.Urea_1
Leaf.Urea_2
Leaf.Urea_3
Nodules.Sym_cond_1
Nodules.Sym_cond_2
Nodules.Sym_cond_3
Root.Standard_1
Root.Standard_2
Root.Standard_3
Roots.NH4_1
Roots.NH4_2
Roots.NH4_3
Roots.NO3_1
Roots.NO3_2
Roots.NO3_3
Roots.Sym_cond_1
Roots.Sym_cond_2
Roots.Sym_cond_3
Roots.Urea_1
Roots.Urea_2
Roots.Urea_3
Roottip.Standard_1
Roottip.Standard_2
Roottip.Standard_3
Seed.Seed.S1_1
Seed.Seed.S1_2
Seed.Seed.S1_3
Seed.Seed.S3_1
Seed.Seed.S3_2
Seed.Seed.S3_3
Seed.Seed.S4_1
Seed.Seed.S4_2
Seed.Seed.S4_3
Seed.Seed.S5_1
Seed.Seed.S5_2
Seed.Seed.S5_3
Seed.Seed.S6_1
Seed.Seed.S6_2
Seed.Seed.S6_3
Seed.Seed.S7_2
Seed.Seed.S7_3
Seed.Seed.S8_1
Seed.Seed.S8_2
Seed.Seed.S9_1
Seed.Seed.S9_2
Seed.Seed.S9_3
Shoot_tip.Standard_1
Shoot_tip.Standard_2
Shoot_tip.Standard_3
Stem.Standard_1
Stem.Standard_2
Stem.Standard_3

Figure.S35

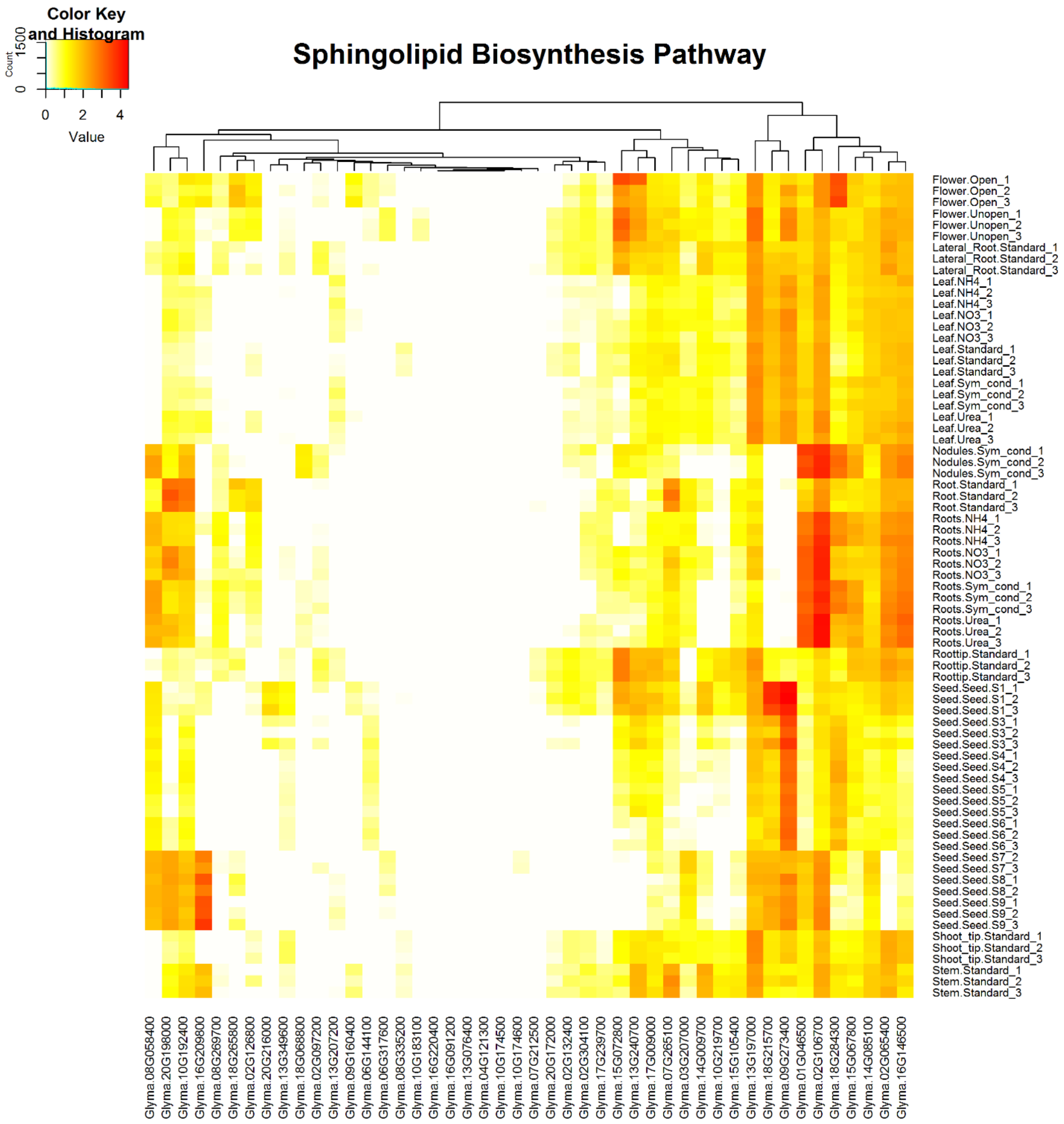
Color Key
and Histogram
Count
0
1500
0
2
4
Value
Sphingolipid Biosynthesis Pathway
Flower.Open_1
Flower.Open_2
Flower.Open_3
Flower.Unopen_1
Flower.Unopen_2
Flower.Unopen_3
Lateral_Root.Standard_1
Lateral_Root.Standard_2
Lateral_Root.Standard_3
Leaf.NH4_1
Leaf.NH4_2
Leaf.NH4_3
Leaf.NO3_1
Leaf.NO3_2
Leaf.NO3_3
Leaf.Standard_1
Leaf.Standard_2
Leaf.Standard_3
Leaf.Sym_cond_1
Leaf.Sym_cond_2
Leaf.Sym_cond_3
Leaf.Urea_1
Leaf.Urea_2
Leaf.Urea_3
Nodules.Sym_cond_1
Nodules.Sym_cond_2
Nodules.Sym_cond_3
Root.Standard_1
Root.Standard_2
Root.Standard_3
Roots.NH4_1
Roots.NH4_2
Roots.NH4_3
Roots.NO3_1
Roots.NO3_2
Roots.NO3_3
Roots.Sym_cond_1
Roots.Sym_cond_2
Roots.Sym_cond_3
Roots.Urea_1
Roots.Urea_2
Roots.Urea_3
Roottip.Standard_1
Roottip.Standard_2
Roottip.Standard_3
Seed.Seed.S1_1
Seed.Seed.S1_2
Seed.Seed.S1_3
Seed.Seed.S3_1
Seed.Seed.S3_2
Seed.Seed.S3_3
Seed.Seed.S4_1
Seed.Seed.S4_2
Seed.Seed.S4_3
Seed.Seed.S5_1
Seed.Seed.S5_2
Seed.Seed.S5_3
Seed.Seed.S6_1
Seed.Seed.S6_2
Seed.Seed.S6_3
Seed.Seed.S7_2
Seed.Seed.S7_3
Seed.Seed.S8_1
Seed.Seed.S8_2
Seed.Seed.S9_1
Seed.Seed.S9_2
Seed.Seed.S9_3
Shoot_tip.Standard_1
Shoot_tip.Standard_2
Shoot_tip.Standard_3
Stem.Standard_1
Stem.Standard_2
Stem.Standard_3
Glyma.08G058400
Glyma.20G198000
Glyma.10G192400
Glyma.16G209800
Glyma.08G269700
Glyma.18G265800
Glyma.02G126800
Glyma.20G216000
Glyma.13G349600
Glyma.18G068800
Glyma.02G097200
Glyma.13G207200
Glyma.09G160400
Glyma.06G144100
Glyma.06G317600
Glyma.08G335200
Glyma.10G183100
Glyma.16G220400
Glyma.16G091200
Glyma.13G076400
Glyma.04G121300
Glyma.10G174500
Glyma.10G174600
Glyma.07G212500
Glyma.20G172000
Glyma.02G132400
Glyma.02G304100
Glyma.17G239700
Glyma.15G072800
Glyma.13G240700
Glyma.17G009000
Glyma.07G265100
Glyma.03G207000
Glyma.14G009700
Glyma.10G219700
Glyma.15G105400
Glyma.13G197000
Glyma.18G215700
Glyma.09G273400
Glyma.01G046500
Glyma.02G106700
Glyma.18G284300
Glyma.15G067800
Glyma.14G085100
Glyma.02G065400
Glyma.16G146500

Figure.S36

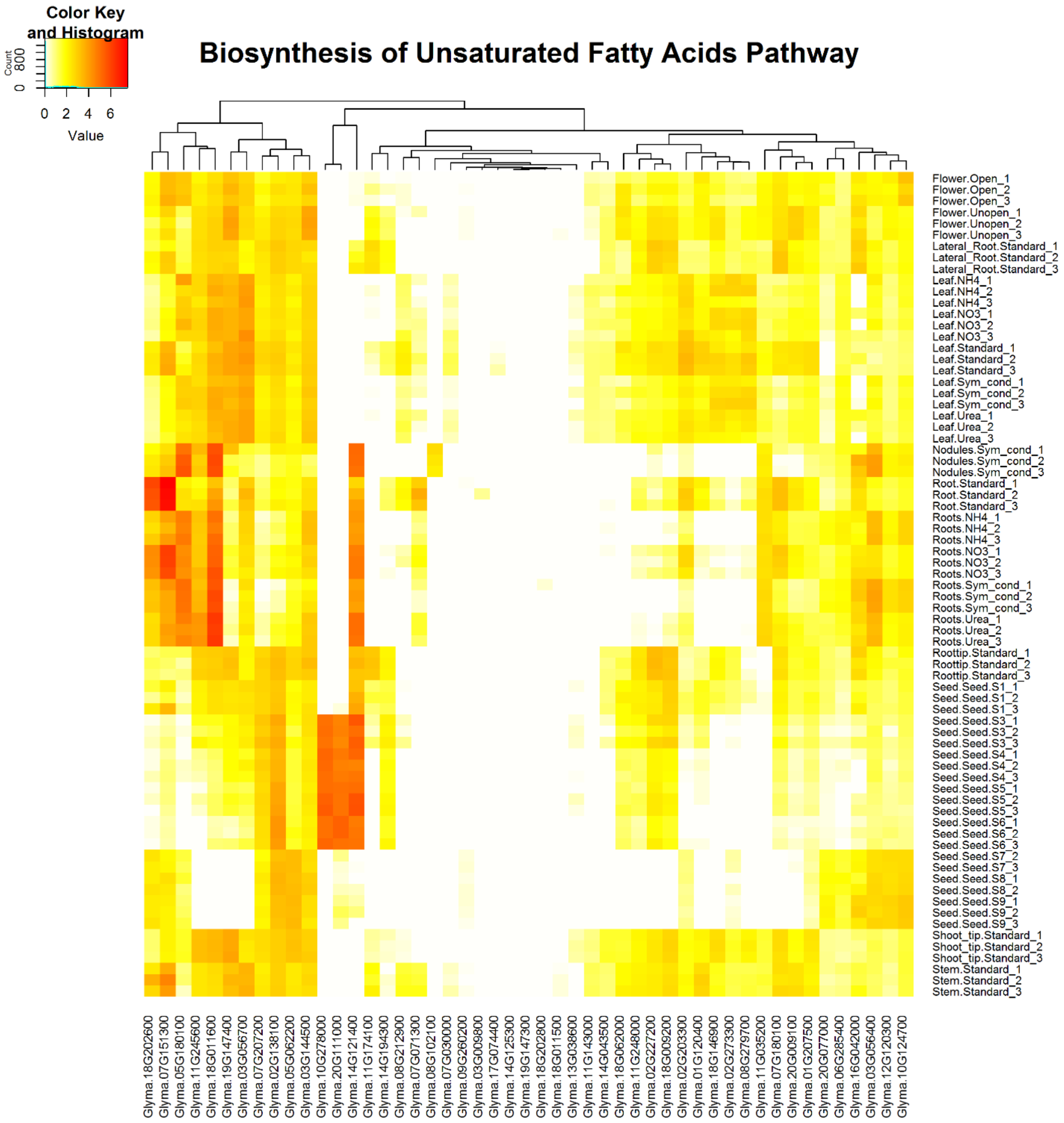
Color Key
and Histogram
Count
0
800
0 2 4 6
Value
Biosynthesis of Unsaturated Fatty Acids Pathway
Flower.Open_1
Flower.Open_2
Flower.Open_3
Flower.Unopen_1
Flower.Unopen_2
Flower.Unopen_3
Lateral_Root.Standard_1
Lateral_Root.Standard_2
Lateral_Root.Standard_3
Leaf.NH4_1
Leaf.NH4_2
Leaf.NH4_3
Leaf.NO3_1
Leaf.NO3_2
Leaf.NO3_3
Leaf.Standard_1
Leaf.Standard_2
Leaf.Standard_3
Leaf.Sym_cond_1
Leaf.Sym_cond_2
Leaf.Sym_cond_3
Leaf.Urea_1
Leaf.Urea_2
Leaf.Urea_3
Nodules.Sym_cond_1
Nodules.Sym_cond_2
Nodules.Sym_cond_3
Root.Standard_1
Root.Standard_2
Root.Standard_3
Roots.NH4_1
Roots.NH4_2
Roots.NH4_3
Roots.NO3_1
Roots.NO3_2
Roots.NO3_3
Roots.Sym_cond_1
Roots.Sym_cond_2
Roots.Sym_cond_3
Roots.Urea_1
Roots.Urea_2
Roots.Urea_3
Roottip.Standard_1
Roottip.Standard_2
Roottip.Standard_3
Seed.Seed.S1_1
Seed.Seed.S1_2
Seed.Seed.S1_3
Seed.Seed.S3_1
Seed.Seed.S3_2
Seed.Seed.S3_3
Seed.Seed.S4_1
Seed.Seed.S4_2
Seed.Seed.S4_3
Seed.Seed.S5_1
Seed.Seed.S5_2
Seed.Seed.S5_3
Seed.Seed.S6_1
Seed.Seed.S6_2
Seed.Seed.S6_3
Seed.Seed.S7_2
Seed.Seed.S7_3
Seed.Seed.S8_1
Seed.Seed.S8_2
Seed.Seed.S9_1
Seed.Seed.S9_2
Seed.Seed.S9_3
Shoot_tip.Standard_1
Shoot_tip.Standard_2
Shoot_tip.Standard_3
Stem.Standard_1
Stem.Standard_2
Stem.Standard_3
Glyma.18G202600
Glyma.07G151300
Glyma.05G180100
Glyma.11G245600
Glyma.18G011600
Glyma.19G147400
Glyma.03G056700
Glyma.07G207200
Glyma.02G138100
Glyma.05G062200
Glyma.03G144500
Glyma.10G278000
Glyma.20G111000
Glyma.14G121400
Glyma.11G174100
Glyma.14G194300
Glyma.08G212900
Glyma.07G071300
Glyma.08G102100
Glyma.07G030000
Glyma.09G260200
Glyma.03G009800
Glyma.17G074400
Glyma.14G125300
Glyma.19G147300
Glyma.18G202800
Glyma.18G011500
Glyma.13G038600
Glyma.11G143000
Glyma.14G043500
Glyma.18G062000
Glyma.11G248000
Glyma.02G227200
Glyma.18G009200
Glyma.02G203300
Glyma.01G120400
Glyma.18G146900
Glyma.02G273300
Glyma.08G279700
Glyma.11G035200
Glyma.07G180100
Glyma.20G009100
Glyma.01G207500
Glyma.20G077000
Glyma.06G285400
Glyma.16G042000
Glyma.03G056400
Glyma.12G120300
Glyma.10G124700

Figure.S37

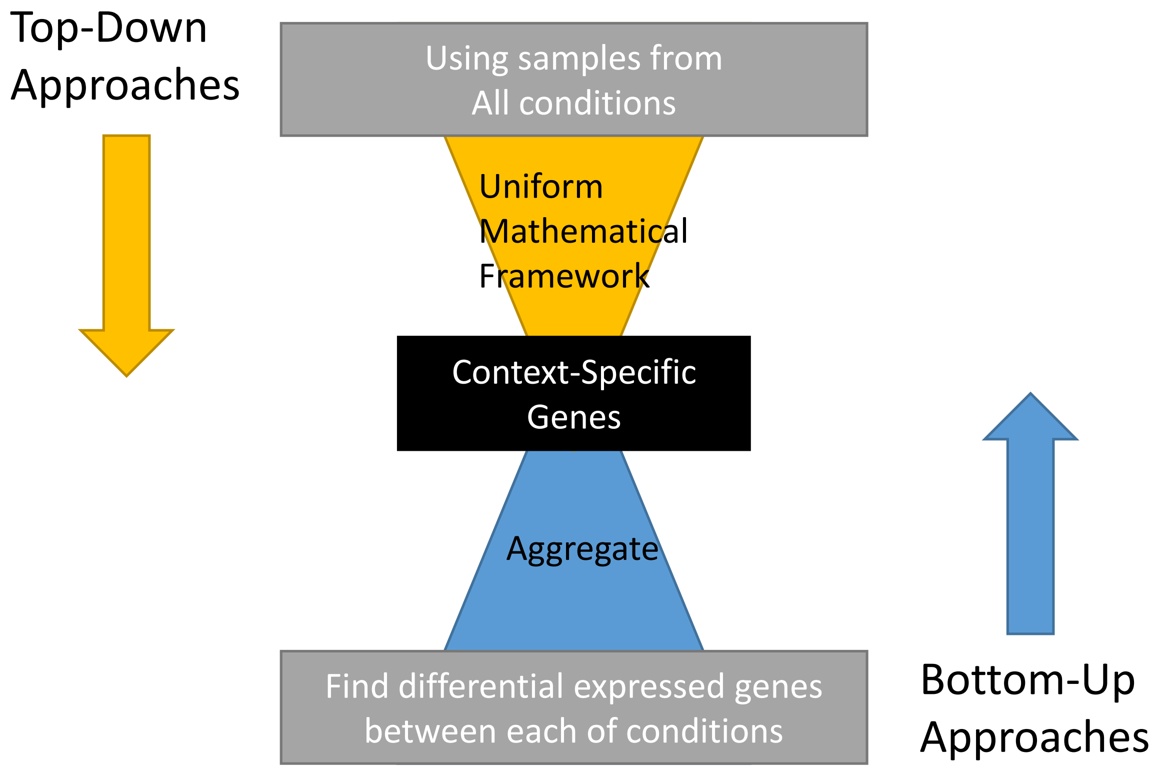

Figure S38:
Schematic analysis from top-down and bottom-up approaches
