# Supplementary material for: SoyCSN: Soybean context‐specific network analysis and prediction based on tissue‐specific transcriptome data
Source: Plant Direct. 2019 Sep 17;3(9):e00167. doi: 10.1002/pld3.167 (PMC6747016; doi:10.1002/pld3.167)
Supplement: Supplementary file 14 [file PLD3-3-e00167-s014.pdf]

Dear Editor:

Thank you for your letter and for the reviewer's comments concerning our manuscript entitled "SoyCSN: Soybean Context-Specific Network Analysis and Prediction Based on Tissue-Specific Transcriptome Data" (Manuscript ID: 2019-00300-T). We have studied every comment carefully and found the reviewer comments very helpful. We have made corrections accordingly. The revised manuscript and response for comments are enclosed as below. Revised portions are highlighted in blue in the main manuscript for ease of identification. We hope the revised version will satisfy all the reviewers comments.

Sincerely yours,

Trupti Joshi

Reviewer #1:

This manuscript present the pipeline for soybean context-specific network of analysis of gene expression at Soykb.
The manuscript is well written and thorough, the study provide significant insights on a method for identification of tissue specific genes and provide information for the case of the soybean tissue-specifics genes.

Majors comment that need to be addressed:
-I have tried to reproduce the figure 5 on the Soykb website and it keep on loading. This issue should be resolved before publication.

Response: Sorry for inconvenience. Our database services were a bit unstable during server updates last months. We have done thorough testing and it should be working fine now. Please check URL: http://soykb.org/SoyCSN/networkgraph.php?v=6&test%5B%5D=Tissue_1&test%5B%5D=Tissue_2&gene=Glyma.13G046200&number=10&method=C3D . You should get same results as shown in Figure 5.

-Description of the edges of the networks are never clearly explicitly describe, only as "context-specific connections". It should be mentioned that they corresponds to level of gene coexpression amongst the studied tissues.

Response: Thank you for your comments. We have added the description in two parts of the manuscript text when describing nodes and edges as explained further: a) In the Results Section, Context-specific network on the 6 combined tissues reconstructed by BicMix: "In each of the six CSNs, nodes are context-specific genes exclusively in this combined tissue, and the edges are context-specific connections corresponding to co-expression levels between these genes." b) In Legend of Figure 4. "Nodes are genes specific to leaf with rhizobium-legume symbiosis, and edges are tissue-specific connections corresponding to gene co-expression levels between these genes."

Minor comments:

-The last paragraph of the introduction is too descriptive of the analysis performed in the manuscript and should be rewritten in a presentation of the different tools instead of sounding like a material and method. The last two sentence are probably sufficient for introducing the present work.

Response: We have reorganized this paragraph accordingly to give readers a brief review and significances of our work in the last portion of the Background section. "To our knowledge, this paper is the first effort conducting a systematic exploration of expression specificities and regulatory specificities on all major soybean tissues using large-scale RNA-Seq data. After processing Gene Atlas RNA-Seq data and PPI information from the STRING database (Szklarczyk et al., 2015), Soybean CSNs on either 6 combined tissues or 14 specific tissues were reconstructed using a set of computational approaches such as BicMix (Gao et al., 2016), C3D (Xiao et al., 2014), ActPro, adaptive_ERW, PenPro (Mohammadi and Grama, 2016), NR (Bossi and Lehner, 2009) and ERW (Magger et al., 2012). These CSNs were systematically analyzed, and functionally annotated with GO enrichment analysis, pathway analysis and regulatory motif analysis. All the results can be effectively retrieved and visualized via a web-based tool in SoyKB, a comprehensive all-inclusive web resource for soybean (Joshi et al., 2017, Joshi et al., 2012, Joshi et al., 2014). In this paper, we mainly focused on analyzing and discussing context specific network results from transcriptome data, while the interactome data based results are available for access and analysis on the website. The whole workflow of this study is shown in Figure 1. This study provides a useful resource for researchers to study tissue specificities in soybean."

-If authors manage to obtain significant results, I would suggest to perform enrichment analysis of the hubs specific to each tissues. For example, it might help to identify important transcription factors controlling the expression of a large set of genes. Also, putative roles and functions of the largest hubs should be described.

Response: Thank you for your suggestion. Annotations in Pfam, Panther of genes including hub genes have already been conducted and details are available in Table S2 and Table S7. According to your suggestions, we have now also added GO enrichment analysis only on hub genes with degree equal or more than 30 as in Tables S6 and S11.

We have also revised text as follows: a) In Section "Context-specific network on the 6 combined tissues reconstructed by BicMix": The hub genes and their annotations are highlighted in Table S2. We added a paragraph in the end of this section: "Considering the importance of hub genes, we also performed Gene Ontology (GO) enrichment analysis only on hub genes with degree equal or more than 30 on each of the CSNs. The results are detailed in Table S6. Flower tissue show significant results as catalytic activity (FDR 0.016) and peptidase activity (FDR 0.016). Seed tissue has significant annotations of function structural molecule activity (FDR 3.30E-06) on ribonucleoprotein complex (FDR 1.00E-06) with translation process (FDR 0.00026)."

b) We also added a paragraph in the end of Section "Context-specific networks for refined 14 representative specific tissues reconstructed by BicMix": "Gene enrichment analysis results only on hub genes with equal or more than 30 degrees in each CSN are detailed in Table S11. Due to the limited

amount of genes, very few CSNs show significant GO annotations, as Root standard CSN with RNA biosynthetic process (FDR 0.0055), and Seed Stage9 with nucleic acid binding (FDR 0.0006). "

Reviewer #2:

This research presents a systematic exploration of several transcriptomic datasets from major soybean tissues under specific contexts. The results of this study eventually became a built-in web service on the Soykb database, which is meaningful and helpful to most soybean-related researchers.

I discovered one major problems in terms of experimental design and technical standard. The title, the abstract, introduction, discussion and methods part presents a substantial content about C3D and interactome network. However, it's not shown in the results part. Although it's practical for such a long descriptive manuscript to have a focus, it doesn't mean the other results can be ignored. It intrigues the reader including me to see the results.

Response: Thank you for your comments. The main reason why we only incorporated BicMix in the paper is due to the paper length limitation. We have also conducted other analysis using other methods, and those results are available on the website. Another reason is the methodology limitation, i.e., outputs of C3D are sets of genes shared by conditions, and different conditions have different weights for connections between these same genes according to their expressions. As mainstream annotations use genes as the input, it is hard to analyze C3D results; so we decided to only include BicMix in the paper.

To reflect this in the text of the paper, a) we have stated that in the end of the Background section: " In this paper, we mainly focused on analyzing and discussing context specific network results from transcriptome data, while the interactome data based results are available for analysis and access directly on the website". b) We also revised some text in Discussion. "Additionally, as only context specific relationships vary among the same shared genes in C3D, it is hard to use any classical gene based annotation methods to analyze their results. Only results from Bicmix are included and described in this manuscript, while other methods results are available on the website. "

Other minor issues:

• One experimental issue I am concerned about is the batch effect between different samples and treatments, which could heavily affect the construction of gene expression network, but this issue seems to be addressed at the very end of the discussion part by saying "homogeneous data source". I suggest this clarification be put at the beginning of the results part.

Response: Thank you for pointing this out. We added text to reflect this in the manuscript "As the data originates from homogeneous sources, we temporally ignore the batch effect between different samples and treatments in this study." in the first section of Results.

• One another question I have is that in the result for 6 combined tissue, the network constructed for shoot-stem combined data is not as well as the others, can they be analyzed separately?

Response: As we adopted the same computational strategy for all the input tissue and treatment, it is usually meaningful to analyze all the CSNs using the same tools and the same criteria. One reason why we combine shoot and stem is due to the gene expression pattern similarity from PCA. See Figure 2, SHT and STE are very close to each other in the context of all the samples. Actually, we have tried to explore different separations as 7 tissues, but it takes too long time to run BicMix, and all the results in other tissues will change as we did in 14 specific tissues. So changing to the separate analysis approach will lead to nearly re-analyzing everything. We think it is beyond this paper's scope. If the readers are interested, they can test any tissue/conditions using our CSN service to get pre-computed C3D and other methods.

• The result part for 14 specific tissues, we can see clearly that some networks have much more nodes and edges than the others? What causes these differences? I suggested it be discussed since it's not mentioned.

Response: Thank you for your suggestion. We added a paragraph to address this phenomenon in Context-specific networks for refined 14 representative specific tissues reconstructed by BicMix: " Different patterns could be observed when comparing constructed CSNs between 6 combined tissues in Table 2 and 14 specific tissues in Table 3. It is much more diverse in specificities from 14 specific tissues than the 6 combined tissues. The differences in mean degree and hub distribution may demonstrate the context-specific network's capability for capturing diverse specificities in one uniform background."

Issues I detected are about writing.

• As a manuscript potentially to be published on a plant science journal, instead of a bioinformatics journal, the writing should be adapted to readers mostly with a biological background rather than computational. For example, the second paragraph of the introduction part is about "top-down" and "bottom-up" strategies comparison but to a person who has no experience in network construction, honestly the introduction is not very intuitive. The following paragraph introduced several network building methods used in this study with abundant technical word, which I think is ok but I suggest adding some comments about what's the benefit of each method or which one to use under different situations.

Response: It is indeed hard for plant researchers to understand these concepts; so we added a schematic figure S38 describing the differences between top-down and bottom-up approaches. In the text, we also added a sentence "Figure S38 demonstrates a schematic of logical differences between top-down and bottom-up analysis approaches." in the background section.

• The font size of vertical labels are too small to see.

Response: We understand the reviewers concern and have improved Figure 3. Due to the limitation of the file size for the submission system, we have updated it to provide high quality graph.

Reviewer #3:

This manuscript describes a pipeline to reconstruct Soybean Context-Specific Network (SoyCSN) using selected transcriptomic data and visualization of the results via an interactive web at SoyKB. In addition, Authors analyze and annotate the networks. Overall, the study was designed appropriately and the results are supportive to claims. SoyCSN web should be valuable to the soybean community.

1. The authors states in Material and Methods: "All the raw RNA-Seq data were collected from the Joint Genome Institute in Gene Atlas project (phytozome.jgi.doe.gov). Aligned and annotated using G. max Gene Model Wm82.a2.v1 (Schmutz et al.,2010), the FPKM values of 56,044 genes were obtained and downloaded". I did not find it. Please provide original RNA seq data (better to depose the data in GEO), detailed description for each tissue and treatment condition, RNA seq depth, RNA accumulation level of each gene, and methods to determine the accumulation of each gene. If they are published previously or deposited in GEO and described in the other web site, please provide the links on soyKB for readers to easily access the data and info. It is important for users to repeat the data analysis, to use the network information with a confidence, and perform the follow-up experiments.

Response: Thank you for your suggestion. As an analysis focused paper, originally our main objective was to focus more on the data preparation steps and analysis steps, as the sequencing related details are in a separate Gene Atlas manuscript, which is currently under revision. According to your suggestion, (1) we added Table S1 with SRR and SRP number, number of raw reads, reads after QC and read pairs aligned to reference genome. (2) We also added some description about the data collection and preprocessing part: "All the raw RNA-Seq data were collected from the Joint Genome Institute in Gene Atlas project (phytozome.jgi.doe.gov). In this project, soybean seeds (G. max William 82) were surface-sterilized, transferred to pots containing 3:1 vermiculite perlite. 2/3 seedlings were planted in each pot and grown until plants were 4 weeks in a growth chamber under 16-h-light/8-h-dark conditions, 26-23°C temperature maintained at 250 µmol $m^{-2}s^{-1}$. Plants for nitrogen experiment were watered with nutrient solution containing either 10 mM KNO_3 (NO_3^- plants) or 10 mM $(NH_4)_3PO_4$ (NH_4^+ plants) or 5 mM urea (urea plants). Urea were selected as a control condition for the counter ions, potassium and phosphate, as the best compromise. The nutrient solutions were renewed every 3 days. After 4 weeks, different tissues (leaf, stem, root, shoot, shoot tip, root tip, lateral roots, etc.) for N regimes and the standard condition were harvested. Plants under the symbiotic conditions were watered with nutrient solution containing 0.5 mM NH_4NO_3 every other week. Subsequently, root nodules, roots, and trifoliate leaves under the symbiotic conditions were collected and tissues from flower open and un-open were harvested from field grown plants. The RNA-seq data that support the findings of this study are available from the NCBI Sequence Read Archive (SRA) under accessions provided as Table S1. More details of the experimental design, plants growth, treatments, library construction and sequencing are described in a separate paper of Gene Atlas project (Sreedasyam et al., Unpublished).

Aligned and annotated using G. max Gene Model Wm82.a2.v1 (Schmutz et al., 2010), the FPKM values of 56,044 genes were obtained through Phytozome interface Phytomine (https://phytozome.jgi.doe.gov/phytomine/template.do?name=Gene_Expression)."

2. Authors provide extensive and excellent description of biological knowledge that authors identified from annotating networks to demonstrate the effectiveness of the strategies. It will make the MS stronger if authors can compare with other methods in identifying related GO terms. However, it is optional for authors since it could be a big task.

Response: Thank you for your suggestion. Yes, comparing with other methods is a huge task which is beyond the scope for this paper, but we decided to add some extra analysis on hub genes in all the CSNs. We conducted GO enrichment analysis only on hub genes with degree equal or more than 30 as in Tables S6 and S11.

We revised text as follows: a) In Section “Context-specific network on the 6 combined tissues reconstructed by BicMix”: The hub genes and their annotations are highlighted in Table S2. We added a paragraph in the end of this section: “Considering the importance of hub genes, we also performed Gene Ontology (GO) enrichment analysis only on the hub genes with degree equal or more than 30 on each of the CSNs. The results are detailed in Table S6. The flower tissue shows significant results as catalytic activity (FDR 0.016) and peptidase activity (FDR 0.016). The seed tissue has significant annotations of function structural molecule activity (FDR 3.30E-06) on ribonucleoprotein complex (FDR 1.00E-06) with translation process (FDR 0.00026).”

b) We also added a paragraph in the end of Section “Context-specific networks for refined 14 representative specific tissues reconstructed by BicMix”: “Gene enrichment analysis results only on the hub genes with equal or more than 30 degrees in each CSN are detailed in Table S11. Due to the limited number of genes, very few CSNs show significant GO annotations, as Root standard CSN with RNA biosynthetic process (FDR 0.0055), and Seed Stage9 with nucleic acid binding (FDR 0.0006). “

3. There is a couple of typo such as _--------------transcriptome-based methods BicMix and C3D in the paragraph subtitled with "SoyCSN web service".

Response: Sorry for the inconvenience. We checked and revised all the text again and have addressed any typos.
